# Tal6b/AvrXa27A, a hidden TALE targeting the susceptibility gene *OsSWEET11a* and the resistance gene *Xa27* in rice

**DOI:** 10.1016/j.xplc.2023.100721

**Published:** 2023-09-20

**Authors:** Zhengyin Xu, Xiameng Xu, Ying Li, Linlin Liu, Qi Wang, Yijie Wang, Yong Wang, Jiali Yan, Guanyun Cheng, Lifang Zou, Bo Zhu, Gongyou Chen

**Affiliations:** 1 Shanghai Collaborative Innovation Center of Agri-Seeds, School of Agriculture and Biology, Shanghai Jiao Tong University, Shanghai 200240, China; 2 State Key Laboratory of Microbial Metabolism, Shanghai Jiao Tong University, Shanghai 200240, China

**Keywords:** *Xanthomonas oryzae* pv. *oryzae*, TALE, AvrXa27, tal6b, *Xa27*, *OsSWEET11a*

## Abstract

*Xanthomonas oryzae* pv. *oryzae* (*Xoo*) secretes transcription activator-like effectors (TALEs) to activate rice susceptibility (*S*) genes, causing bacterial blight (BB), as well as resistance (*R*) genes, leading to defense against BB. This activation follows a gene-for-gene paradigm that results in an arms race between the TALE of the pathogen and effector-binding elements (EBEs) in the promoters of host genes. In this study, we characterized a novel TALE, designated Tal6b/AvrXa27A, that activates the rice *S* gene *OsSWEET11a* and the rice *R* gene *Xa27*. Tal6b/AvrXa27A is a member of the AvrXa27/TalAO class and contains 16 repeat variable diresidues (RVDs); one RVD is altered and one is deleted in Tal6b/AvrXa27A compared with AvrXa27, a known avirulence (*avr*) effector of *Xa27*. Tal6b/AvrXa27A can transcriptionally activate the expression of *Xa27* and *OsSWEET11a* via EBEs in their corresponding promoters, leading to effector-triggered immunity and susceptibility, respectively. The 16 RVDs in Tal6b/AvrXa27A have no obvious similarity to the 24 RVDs in the effector PthXo1, but EBE_Tal6b_ and EBE_PthXo1_ are overlapped in the *OsSWEET11a* promoter. Tal6b/AvrXa27A is prevalent among Asian *Xoo* isolates, but PthXo1 has only been reported in the Philippine strain PXO99^A^. Genome editing of EBE_Tal6b_ in the *OsSWEET11a* promoter further confirmed the requirement for *OsSWEET11a* expression in Tal6b/AvrXa27A-dependent susceptibility to *Xoo*. Moreover, Tal6b/AvrXa27A resulted in higher transcription of *Xa27* than of *OsSWEET11a,* which led to a strong, rapid resistance response that blocked disease development. These findings suggest that Tal6b/AvrXa27A has a dual function: triggering resistance by activating *Xa27* gene expression as an avirulence factor and inducing transcription of the *S* gene *OsSWEET11a*, resulting in virulence. Intriguingly, Tal6b/AvrXa27A, but not AvrXa27, can bind to the promoter of *OsSWEET11a*. The underlying recognition mechanism for this binding remains unclear but appears to deviate from the currently accepted TALE code.

## Teaser

This study reports the identification of Tal6b/AvrXa27A, a novel TALE in *Xoo* that activates the *S* gene *OsSWEET11a* and the *R* gene *Xa27* in rice, performing a dual role in ETS and ETI. Further characterization shows its distinct RVD composition and promoter interactions as well as its prevalence among Asian isolates. Tal6b/AvrXa27A triggers resistance via *Xa27* activation while inducing *OsSWEET11a* for virulence, shaping the gene-for-gene arms race.

## Introduction

Effectors are proteins secreted by pathogens into or near host cells that increase pathogen virulence (Koseoglou et al., 2022). Effectors can interfere with pathogen-associated molecular pattern-triggered immunity, which is the first layer of the plant innate immune system; this interference can result in effector-triggered susceptibility (ETS) (Jones and Dangl, 2006). In some cases, effectors are intercepted by host resistance (*R*) genes that activate effector-triggered immunity (ETI) (Jones and Dangl, 2006; Zhou and Zhang, 2020). Transcription activator-like effectors (TALEs) are translocated into host plants via the type III secretion system in phytopathogenic bacteria of the genera *Xanthomonas* and *Ralstonia* (Boch and Bonas, 2010; Boch et al., 2014; Xu et al., 2017). After injection into plant cells, TALEs bind to EBEs (effector-binding elements), which are sequences in the promoters of host genes; activation of these genes can reprogram the host transcriptome (Boch et al., 2014). The specificity of EBE binding is determined by repeat-variable diresidues (RVDs) that are located at amino acid residues 12 and 13 in the central repeat regions (CRRs) of TALEs (Boch et al., 2009; Moscou and Bogdanove, 2009). Genes targeted and activated by TALEs include host susceptibility (*S*) and *R* genes (Yang and White, 2004; White and Yang, 2009; Zhang et al., 2015).

Activation of *S* genes can result in compatible host–pathogen interactions and disease symptoms (White and Yang, 2009; Hutin et al., 2015a; Koseoglou et al., 2022). The bacterial blight (BB) pathogen, *Xanthomonas oryzae* pv. *oryzae* (*Xoo*), is known to manipulate the rice *SWEET* gene family (Niño-Liu et al., 2006; Streubel et al., 2013). Three *SWEET* genes in clade III are targeted by nine major TALEs. These *SWEET* genes and their targets include *OsSWEET11a* (also known as *OsSWEET11/Xa13*), which is targeted by PthXo1 (Yang et al., 2006), and *OsSWEET13* (*Xa25/xa25*), which is induced by several PthXo2-like TALEs (PthXo2, PthXo2B/Tal7_PXO61_, PthXo2C/Tal5_LN18_, and Tal7_K74_) (Zhou et al., 2015; Xu et al., 2019; Oliva et al., 2019). *OsSWEET14* is induced by AvrXa7, PthXo3, TalC, and TalF/Tal5, which target the *OsSWEET14* promoter at different or overlapping EBEs (Antony et al., 2010; Yu et al., 2011; Streubel et al., 2013; Tran et al., 2018). Three other *SWEET* genes, *OsSWEET12*, *OsSWEET15*, and *OsSWEET11b*, are potential *S* genes for BB that lack corresponding TALEs (Streubel et al., 2013; Wu et al., 2022). It is important to note that unidentified TALEs likely exist in *Xoo* strains isolated from rice paddies.

Approximately 47 *R* genes that confer resistance to BB have been reported in rice, and at least 17 *R* genes have been cloned and characterized (Jiang et al., 2020; Xing et al., 2021; Xu et al., 2022; Zhang et al., 2022). The functions of eight *R* genes are directly related to the process of TALE-mediated host-gene induction (Jiang et al., 2020; Zhang et al., 2022); these include four recessive *R* genes (*xa5*, *xa13*, *xa25*, and *xa41*) (Hutin et al., 2015a; 2015b) and four dominant TALE-dependent *R* genes known as executor (*E*) genes (*Xa7*, *Xa10*, *Xa23*, and *Xa27*) (Zhang et al., 2015; Ji et al., 2022). *Xa27* was originally derived from wild rice (*Oryza minuta*) and confers dominant resistance to BB (Gu et al., 2004; 2005). *Xa27* harbors an EBE in its promoter that recognizes the cognate TALE AvrXa27 (Gu et al., 2005; Römer et al., 2009). The Xa27 protein contains a signal-anchor-like sequence in its N terminus that is essential for subcellular localization and resistance to *Xoo* (Gu et al., 2005; Wu et al., 2008).

Among the TALEs identified in *Xoo*, AvrXa7 and PthXo3 are unique because they have dual functions as virulence and avirulence factors; both induce the *S* gene *SWEET14* (leading to virulence and disease) and trigger resistance in *Xa7*-containing rice (Antony et al., 2010; Römer et al., 2010; Chen et al., 2021; Luo et al., 2021). The EBEs that recognize AvrXa7 and PthXo3 in the *Xa7* promoter exhibit partial overlap, which is also true for corresponding EBEs in the *OsSWEET14* promoter (Chen et al., 2021; Luo et al., 2021). *Xa7*-mediated BB resistance is considered to be broad and durable because ETI will be triggered by *Xoo* strains harboring either AvrXa7 or PthXo3; moreover, these two TALEs also function as major virulence determinants, and their loss penalizes the pathogen with reduced virulence and fitness (Zhao and Zhang, 2021). Consequently, *Xa7* is regarded as a valuable gene for breeding BB-resistant rice. It remains unclear whether other *R* genes, such as *Xa27*, can be activated by cognate avirulence factors that have dual functions and also induce *S* genes.

In the current study, we show that *Xoo* strains LN18 and LN4, which lack *avrXa27*, remain incompatible with the rice cultivar (cv.) 78-15, which harbors *Xa27*. The genomic sequences of LN18 and LN4 revealed that both encode a TALE from the AvrXa27/TalAO class (designated Tal6b/AvrXa27A) that triggers *Xa27-*mediated resistance. Four variants of TALEs in the AvrXa27/TalAO class are present in *Xoo* strains and function as avirulence factors that mediate *Xa27* resistance. Tal6b/AvrXa27A also activated *OsSWEET11a* expression and enhanced susceptibility to *Xoo* in rice. EBE prediction and gel shift assays revealed that Tal6b/AvrXa27A binds directly to the *Xa27* and *OsSWEET11a* promoters. An EBE_Tal6b_ mutation in the *OsSWEET11a* promoter rendered rice susceptible to *Xoo* strains harboring Tal6b/AvrXa27A. Our results indicate that Tal6b/AvrXa27A recognizes the *R* gene *Xa27* and the *S* gene *OsSWEET11a* and suggest that *Xa27* may mediate broad-spectrum durable resistance to BB in a manner analogous to *Xa7*.

## Results

### *Xa27* rice is resistant to *Xoo* strains LN18 and LN4, which do not contain *avrXa27*

**Figure 1:**
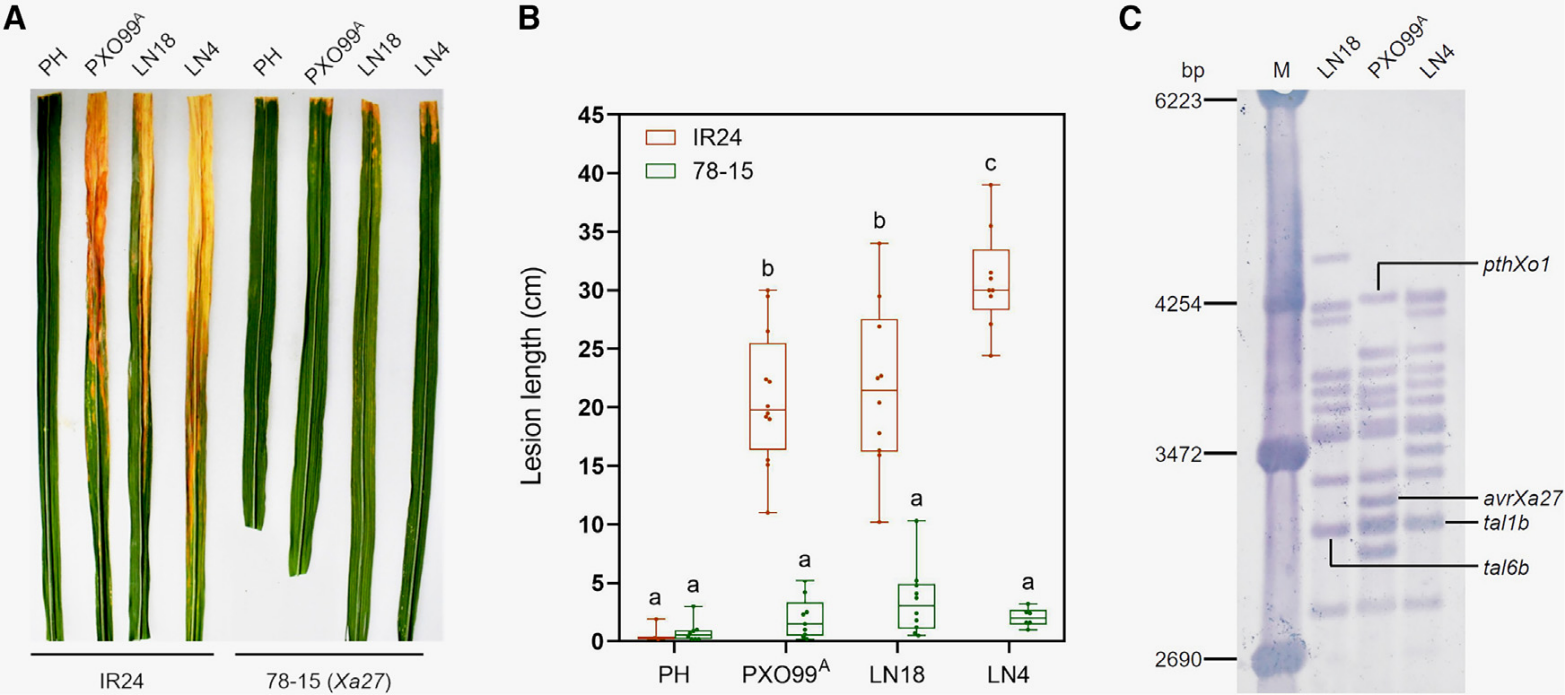
*Xoo* strains LN18 and LN4 do not cause disease on *Xa27*-containing rice. **(A)** Disease symptoms on rice cultivars IR24 and 78-15 after inoculation with *Xoo* strains PXO99^A^, PH, LN18, and LN4. The images were taken 14 days post-inoculation (dpi). **(B)** Boxplots of mean disease lesion lengths (cm) on cvs. IR24 and 78-15. Lesions were measured at 14 dpi; dots denote individual observations from at least 5 inoculated leaves, and whiskers display the first and third quartiles, split by the median. Values with the same lowercase letters do not differ significantly at *P* < 0.05 according to ANOVA. **(C)** Genomic digest of *tal* genes in *Xoo* strains PXO99^A^, LN18, and LN4 as determined by Southern blot analysis. Genomic DNA was digested with *Bam*HI, blotted onto nylon membranes, and hybridized with the *Sph*I fragment from *avrXa27*.

The *Xa27* gene is a broad-spectrum *R* gene that confers resistance to 27 of 35 *Xoo* strains collected from 11 countries (Gu et al., 2004; 2005). To determine whether *Xa27* confers resistance to the hypervirulent *Xoo* strains LN18 and LN4 (Xu et al., 2019; 2020), rice cvs. 78-15 (with *Xa27*) and IR24 (without *Xa27*) were inoculated with the two strains by the tip-cutting method. Lesion lengths and symptoms on 78-15 and IR24 leaves inoculated with *Xoo* LN18 and LN4 were similar to those elicited by *Xoo* PXO99^A^, which contains *avrXa27* (Figure 1A and 1B). Our results indicated that *Xoo* LN18 and LN4 are incompatible with rice cv. 78-15.

To determine whether *Xoo* LN18 and LN4 contain *avrXa27* (an *avr* gene that recognizes *Xa27*), restriction digests of *tal* genes in the two strains were hybridized with a probe that encompassed the CRR of *avrXa27* (GenBank: AY986494). The fragment containing *avrXa27* was absent from Southern blots of *Xoo* LN18 and LN4 (Figure 1C), suggesting that these two strains do not contain *avrXa27*.

**Figure 2:**
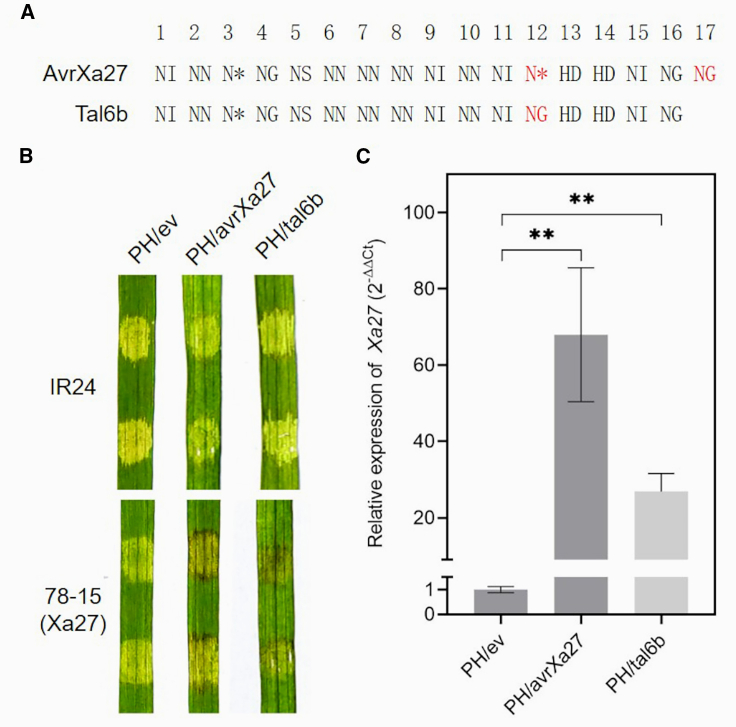
Tal6b, similar to AvrXa27, triggers *Xa27* resistance. **(A)** Repeat variable diresidues (RVDs) in AvrXa27 and Tal6b. Single letters represent amino acids at the 12th and 13th positions of individual repeats. The asterisk indicates a predicted missing 13th residue, and RVDs in red font differ between Tal6b and AvrXa27. **(B)** Reaction of IR24 and 78-15 rice leaves to *Xoo* strains PH/ev, PH/avrXa27, and PH/tal6b. Bacterial suspensions were infiltrated into rice leaves using needleless syringes, and leaves were photographed at 3 dpi. **(C)** Expression of *Xa27* in 78-15 rice leaves inoculated with *Xoo* strains. The expression of *Xa27* was measured in leaves infiltrated with *Xoo* PH/ev (control), PH/avrXa27, and PH/tal6b. RNA was extracted from leaves at 24 hpi and used for qRT–PCR with *Xa27*-specific primers. *Actin* expression was used as an internal control. Columns labeled with two asterisks are significantly different at *P* < 0.01 (Student’s *t*-test).

The incompatibility of *Xoo* LN18 and LN4 with rice cv. 78-15 (*Xa27*) (Figure 1A and 1B) and the lack of an *avrXa27*-hybridizing band in these two strains (Figure 1C) prompted us to speculate that LN18 and LN4 may harbor a functional equivalent of *avrXa27*. Using the LN18 and LN4 genome sequences (Xu et al., 2019; 2020), we identified two orthologs of *avrXa27*, designated *tal6b* and *tal1b* (Supplemental Figure 14), in LN18 and LN4, respectively (Figure 1C). Although the PXO99^A^ strain also contained a band similar to *tal6b* and *tal1b* in the Southern blot (Figure 1C), this band contained *Bam*HI fragments of two *tal* genes, *tal4* and *tal5a*, which are quite different from *avrXa27* (Salzberg et al., 2008). Sequence analysis of *tal6b* from *Xoo* LN18 indicated that the gene comprises 3312 bp with 95.8% nucleic acid identity to *avrXa27* from PXO99^A^, indicating that *tal6b* encodes an AvrXa27-like TALE (designated Tal6b) of 1103 amino acid residues with 16 RVD repeats (Figure 2A). Differences occurred at the 12th RVD, where N∗ in AvrXa27 is NG in Tal6b; furthermore, the 17th RVD is missing in Tal6b (Figure 2A). The RVD array of Tal1b in LN4 is identical to that of Tal6b (Xu et al., 2020). These results indicate that Tal6b is a functional equivalent of AvrXa27; hence, we refer to Tal6b as AvrXa27A in this study.

### Tal6b/AvrXa27A, similar to AvrXa27, triggers *Xa27* resistance

To verify that Tal6b is a variant of AvrXa27, the isolated *tal6b* was subcloned as plasmid pHZW-tal6b (Supplemental Table 1); this construct was transferred into *Xoo* strain PH, a *tal*-free derivative of PXO99^A^ (Ji et al., 2016), resulting in the *Xoo* strain PH/tal6b (Supplemental Table 1). The *Xoo* PH/avrXa27 strain was used as a control (Supplemental Table 1). Western blot analysis showed that Tal6b and AvrXa27 were detected as 120-kDa proteins in the corresponding *Xoo* strains (Supplemental Figure 1). *Xoo* strains PH/tal6b, PH/avrXa27, and PH/ev were infiltrated into seedling leaves of rice cv. 78-15 using needleless syringes and also inoculated by the tip-cutting method. PH/tal6b, which contains a derivative of *avrXa27*, elicited a hypersensitive response (HR) in rice cv. 78-15, as did AvrXa27 (Figure 2B). Lesion lengths on leaves of rice cv. 78-15 inoculated with PH/tal6b did not differ significantly from those on leaves inoculated with PH/avrXa27 (Supplemental Figure 2), implying that Tal6b is a functional equivalent of AvrXa27.

Gene expression assays were used to determine whether Tal6b (AvrXa27A) could activate *Xa27* expression in 78-15 rice. Expression of *Xa27* was significantly higher in 78-15 rice leaves inoculated with *Xoo* PH/avrXa27 and PH/tal6b than in leaves infiltrated with *Xoo* PH/ev (control) (Figure 2C). Collectively, these results suggest that *tal6b* functions as a derivative of *avrXa27*.

### Prevalence of the AvrXa27/TalAO class in *Xoo* strains

The AvrXa27/TalAO class has been categorized previously as a “core TALE” class that is present in over 80% of Asian *Xoo* strains (Mücke et al., 2019). We investigated the distribution of *avrXa27* and homologs by performing BLAST searches of the complete genome sequences of 80 *Xoo* strains in the NCBI database. These strains originated from Asia (47 strains), Africa (31), South America (1), and Oceania (1) and represent diverse geographic areas. Our results showed that 36 *Xoo* strains contained TALEs of the AvrXa27/TalAO class, whereas 44 strains lacked AvrXa27/TalAO orthologs, including all African strains and 13 Asian strains (Supplemental Table 2). AvrXa27/TalAO class TALEs in these 36 *Xoo* strains were grouped into 4 versions based on their RVD sequences (Table 1): (1) the AvrXa27 prototype in 12 Asian and 1 South American strain; (2) the AvrXa27A type in 18 Asian strains, including LN18 and LN4, in which the 12th RVD (NG) is altered and the 17th RVD is missing; (3) the AvrXa27B type, as in strain HuN37, in which the 9th (NN) and the 12th (NG) RVDs are altered and the 17th RVD is missing; and (4) the AvrXa27C type in 2 Indian, 1 Thai, and 1 Australian strain, in which the 12th (NG), 16th (HG), and 17th (N∗) RVDs are altered (Table 1). These RVD differences illustrate the diversity in the AvrXa27/TalAO class of TALEs, which is likely due to evolutionary pressure between the *avrXa27* locus and the cognate *Xa27 R* gene. Moreover, AvrXa27A appears to have a prominent role in the AvrXa27/TalAO class because it is present in 48.65% of 37 *Xoo* strains.

**Table 1:** Repeat variable diresidue (RVD) sequences in TALEs of the AvrXa27/TalAO class in selected *Xoo* strains.

TALE	RVDs^a^																	Strain
	1	2	3	4	5	6	7	8	9	10	11	12	13	14	15	16	17	
AvrXa27 (35.14%)	NI	NN	N∗	NG	NS	NN	NN	NN	NI	NN	NI	N∗	HD	HD	NI	NG	NG	PXO99^A^, PXO86, MAFF 311018 (T7174), PXO83, PXO145, PXO211, PXO236, ICMP3125, JP01, JL33, YC11, K3, CIAT
AvrXa27A/Tal6b (48.65%)	NI	NN	N∗	NG	NS	NN	NN	NN	NI	NN	NI	NG	HD	HD	NI	NG		LN18, LN4, PXO61, PXO71, PXO142, PXO282, PXO404, PXO421, PXO513, PXO524, PXO563, PXO602, KXO85, JW11089, K1, K2, XF89b, XM9
AvrXa27B (2.70%)	NI	NN	N∗	NG	NS	NN	NN	NN	NN	NN	NI	NG	HD	HD	NI	NG		HuN37
AvrXa27C (13.51%)	NI	NN	N∗	NG	NS	NN	NN	NN	NI	NN	NI	NG	HD	HD	NI	HG	N∗	IX-280, BXO1, SK2-3, AUST2013, IXO221^b^

∗ The RVDs in italic fontdiffer from those in AvrXa27.

b IXO221 is an Indian strain containing AvrXa27C; the whole genome sequence is not available.

*Xoo* strains PXO99^A^, HuN37, and IXO221, containing AvrXa27, Tal17/AvrXa27B, and Tal6c/AvrXa27C, respectively, were chosen for further evaluation of their interactions with *Xa27*. Inoculation assays showed that *Xoo* PXO99^A^, HuN37, and IXO221 were avirulent on rice cv. 78-15 containing *Xa27*, which indicates that Tal17/AvrXa27B and Tal6c/AvrXa27C may also trigger *Xa27* resistance (Supplemental Figure 3). To evaluate this possibility, *tal17* and *tal6c* were cloned from *Xoo* HuN37 and IXO221, respectively, and introduced into *Xoo* PH. The resulting strains, *Xoo* PH/tal17 and PH/tal6c, both elicited an HR on rice 78-15 (Supplemental Figure 4), which indicates that Tal17 and Tal6c are orthologs of AvrXa27 and interact with *Xa27*. In addition, qRT–PCR assays revealed a significant upregulation of *Xa27* gene expression in rice inoculated with both PH/tal17 and PH/tal6c strains compared with the control (Supplemental Figure 5), confirming that Tal17/AvrXa27B and Tal6c/AvrXa27C can activate the expression of the *Xa27* gene. Thus, all four members of the AvrXa27/TalAO class triggered ETI via *Xa27*, which helps explain the broad-spectrum resistance of the *Xa27* executor *R* gene.

### Tal6b/AvrXa27A is a novel virulence factor for BB

**Figure 3:**
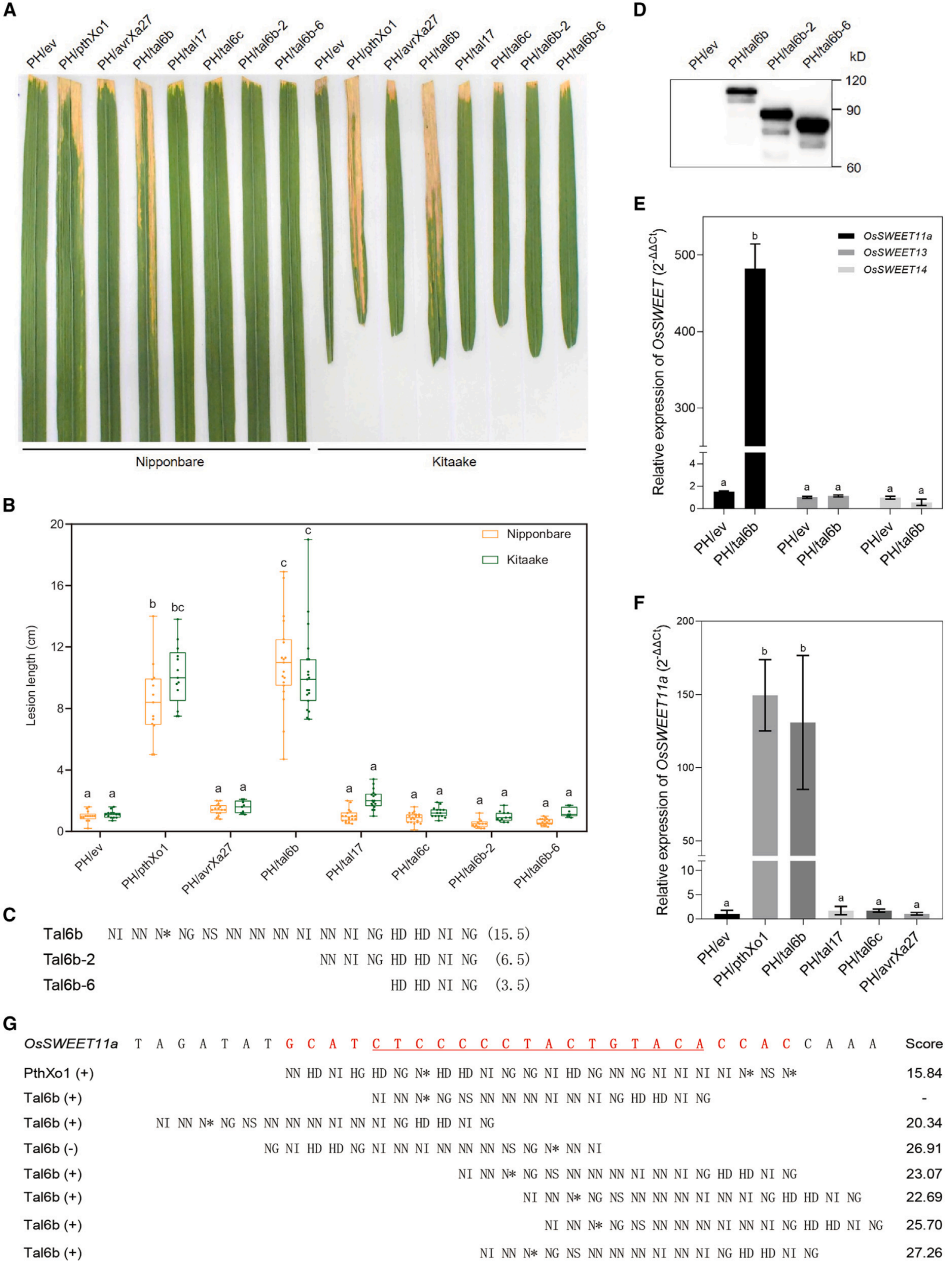
Tal6b/AvrXa27A is a novel major virulence factor that targets the *S* gene *OsSWEET11a*. **(A)** Disease symptoms on rice cultivars Nipponbare and Kitaake after inoculation with *Xoo* strains PH/ev, PH/pthXo1, PH/avrXa27, PH/tal6b, PH/tal17, PH/tal6c, PH/tal6b-2, and PH/tal6b-6. Photos were taken at 14 dpi. **(B)** Boxplots of mean disease lesion lengths (cm) on Nipponbare and Kitaake. Lesions were measured at 14 dpi; dots denote individual observations from at least 5 inoculated leaves, and whiskers display the first and third quartiles, split by the median. **(C)** Individual RVDs of Tal6b and its derivatives Tal6b-2 and Tal6b-6. Single letters denote amino acids at the 12th and 13th positions of individual repeats. An asterisk indicates a predicted missing 13th residue. **(D)** Western blot analysis of TALE proteins in *Xoo* PH/ev, PH/tal6b, PH/tal6b-2, and PH/tal6b-6. **(E)** Expression of *OsSWEET* genes in Kitaake rice leaves inoculated with *Xoo* strains. The expression levels of *OsSWEET11a*, *OsSWEET13*, and *OsSWEET14* were measured in rice leaves infiltrated with *Xoo* PH/ev and PH/tal6b*.* RNA was extracted from leaves 24 hpi and used for qRT–PCR with *OsSWEET11a-*, *OsSWEET13-*, and *OsSWEET14*-specific primers. The expression level of *Actin* was used as an internal control. **(F)** Expression of *OsSWEET11a* in Kitaake rice leaves inoculated with *Xoo* PH strains containing the empty vector (PH/ev) and AvrXa27/TalAO-class TALEs (*pthXo1*, *tal6b*/*avrXa27A*, *tal17/avrXa27B*, *tal6c/avrXa27C*, and *avrXa27*). **(G)** Individual RVDs in the CCR region of PthXo1 and Tal6b/AvrXa27A recognize individual nucleotides in the predicted EBE regions of the *OsSWEET11a* promoter. Single letters denote amino acids at the 12th and 13th positions of individual repeats, and the asterisk denotes a missing amino acid at the 13th position of a particular repeat. The scores show matches between DNA sequences in the *OsSWEET11a* EBE and amino acid residues in the RVDs of Tal6b/AvrXa27A and PthXo1 (positive control); scores were predicted with the TALE-NT program. Lower scores indicate higher binding affinity between the RVDs and the target sequence. EBE_PthXo1_ is shown in red font, and EBE_Tal6b_ is underlined. (+) refers to the forward strand, and (−) refers to the reverse strand. Values with the same lowercase letters do not differ significantly at *P* < 0.05 according to ANOVA.

Introduction of *tal6b* into *Xoo* PH resulted in a high level of virulence on rice cv. IR24 (Supplemental Figure 2), indicating that Tal6b/AvrXa27A may also be a virulence factor. To determine whether other TALEs in the AvrXa27/TalAO class contribute to virulence, PH strains expressing the four *avrXa27* variants were inoculated onto Nipponbare and Kitaake rice using the tip-cutting method. Only *talb6* (*avrXa27A* variant) rendered PH highly virulent on Nipponbare and Kitaake at 14 days post inoculation (dpi) (Figure 3A and 3B). PH/tal6b induced BB lesions exceeding 10.0 cm in length on Nipponbare and Kitaake rice; the other three forms of *avrXa27* induced small lesions less than 3.0 cm in length (Figure 3B). Thus, our results indicate that Tal6b/AvrXa27A functions as a virulence factor in *Xoo*.

The CRRs in TALEs are known to determine their specificity (Boch et al., 2009; Moscou and Bogdanove, 2009); we therefore constructed deletion derivatives of *tal6b* that lacked various repeats to further validate the function of Tal6b as a virulence factor. Derivatives Tal6b-2 and Tal6b-6 lacked repeats 1–9 and 1–12, respectively (Figure 3C and 3D). When these derivatives were introduced into *Xoo,* strains PH/tal6b-2 and PH/tal6b-6 lost their ability to induce disease symptoms in Nipponbare and Kitaake rice (Figure 3A and 3B). Overall, our data suggested that Tal6b/AvrXa27A was a novel virulence factor targeting an unknown *S* gene in rice.

### Tal6b/AvrXa27A targets the well-known *S* gene *OsSWEET11a*

We previously demonstrated that rice lines harboring triple mutations in the promoter regions of three *S* genes (*OsSWEET11a*, *OsSWEET13*, and *OsSWEET14*) exhibited broad-spectrum resistance against all tested *Xoo* strains, including LN18, which contains *tal6b* (Xu et al., 2019). To understand how Tal6b/AvrXa27A promotes disease, we sought to identify its *S* gene target. We speculated that Tal6b/AvrXa27A might target one or more of the three *OsSWEET* genes in rice. To test this hypothesis, rice Kitaake leaves were infiltrated with PH/tal6b and PH/ev, which carried *tal6b*/*avrXa27A* and an empty vector, respectively. The expression levels of *OsSWEET11a* were hundreds of times higher in rice plants infected with PH/tal6b than PH/ev, whereas *OsSWEET13* and *OsSWEET14* were not induced by *Xoo* PH/tal6b (Figure 3E); this suggested that Tal6b/AvrXa27 could transcriptionally activate the *S* gene *OsSWEET11a*. These findings suggested that Tal6b/AvrXa27A has a dual function; in other words, it triggers resistance by interacting with *Xa27* as an avirulence factor, and it induces transcription of the *S* gene *OsSWEET11a*, resulting in virulence.

To test whether other AvrXa27/TalAO-class TALEs also activate *OsSWEET11a*, we evaluated expression of *OsSWEET11* in Kitaake rice leaves inoculated with PH strains containing the empty vector (PH/ev), PthXo1 (PH/pthXo1), and selected AvrXa27/TalAO-class TALEs (*tal6b*/*avrXa27A*, *tal17/avrXa27B*, *tal6c/avrXa27C*, and *avrXa27*). Gene expression analysis showed that *pthXo1* and *tal6b* activated *OsSWEET11a* transcription, but *tal17/avrXa27B*, *tal6c/avrXa27C*, and *avrXa27* did not (Figure 3F).

### Tal6b/AvrXa27A binds to the promoters of *Xa27* and *OsSWEET11a*

To analyze the Tal6b**/**AvrXa27A inducibility of the *OsSWEET11a* and *Xa27* promoters, we cloned the promoter regions of *OsSWEET11a* and *Xa27* upstream of a *gusA* reporter gene in pCAMBIA1381. The two promoter::GUS fusions were individually co-expressed in *Nicotiana benthamiana* with pHB-tal6b by *Agrobacterium*-mediated transformation. GUS (β-glucuronidase) activities revealed that the promoters of *OsSWEET11a* and *Xa27* were responsive to Tal6b**/**AvrXa27A (Supplemental Figure 6).

Activation of target genes by TALEs is dependent on the presence of an EBE in the target gene promoter (Boch et al., 2009; Moscou and Bogdanove, 2009); we therefore searched for possible EBEs recognizing Tal6b**/**AvrXa27A in the promoters of *Xa27* (designated EBE_AvrXa27A_) and *OsSWEET11a* (designated EBE_Tal6b_) using the programs TALE-NT and TALgetter (Figure 3G and Supplemental Figure 7; Doyle et al., 2012; Grau et al., 2013). The putative EBE_AvrXa27A_ in Xa27 is a 16-bp sequence located 86–71 bp upstream of the *Xa27* ATG (Supplemental Figure 8). EBE_AvrXa27A_ overlaps with the sequence of EBE_AvrXa27_, and the predicted binding scores are similar (Supplemental Figure 8) (Römer et al., 2009). Six EBEs for Tal6b/AvrXa27A were predicted near EBE_PthXo1_; these are located −256 to −223 bp upstream of the transcriptional start site of *OsSWEET11a* and have similar TALE-NT scores (Figure 3G).

**Figure 4:**
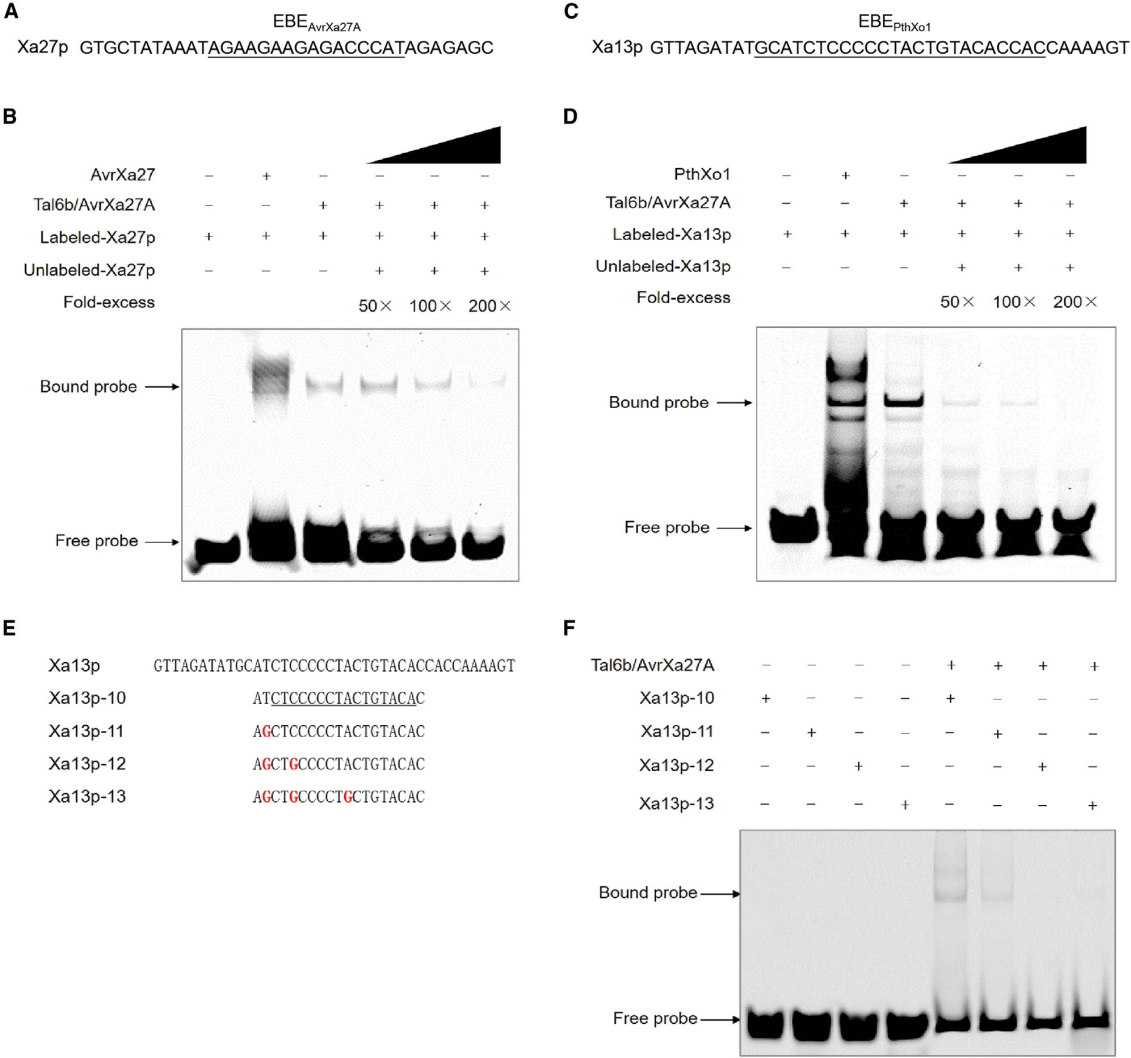
Tal6b/AvrXa27A binds to the EBEs of *Xa27* and *OsSWEET11a*. **(A)** Nucleotide sequence of the *Xa27* promoter fragment (probe); the underscored nucleotides show the EBE recognized by Tal6b/AvrXa27A. **(B)** His-tagged Tal6b/AvrXa27A fusion protein binds to the Xa27p probe derived from the *Xa27* promoter. Positions of the bound and free probe are indicated on the left. **(C)** Nucleotide sequence of the *OsSWEET11a* promoter fragment (probe); EBE_PthXo1_ is underscored. **(D)** His-tagged PthXo1 binds to the Xa13p probe of the *OsSWEET11a* promoter in gel-shift assays. Positions of the bound and free probe are indicated at the left. **(E)** Alignment of Xa13p, Xa13p-10, Xa13p-11, Xa13p-12, and Xa13p-13 sequences. EBE_Tal6b_ is underlined. Mutations in the *OsSWEET11a* promoter sequence are indicated in red font. **(F)** Binding of His-tagged Tal6b/AvrXa27A to EBE_Tal6b_ in the *OsSWEET11a* promoter.

We next investigated whether Tal6b/AvrXa27A binds to the predicted EBEs of *Xa27* and *OsSWEET11a* by electrophoretic mobility shift assays (EMSAs). The His-tagged fusion proteins His-Tal6b/AvrXa27A, His-AvrXa27, and His-PthXo1 were overproduced in *Escherichia coli* and purified. A 34-bp double-stranded DNA fragment containing EBE_AvrXa27A_ (Xa27p) was used to test the interaction of Xa27 with His-Tal6b/AvrXa27A (Figure 4A). EMSA results indicated that Tal6b/AvrXa27A binds to EBE_AvrXa27A_ (Figure 4B). Detection of labeled Xa27p was reduced by addition of unlabeled Xa27p (Figure 4B), demonstrating that this binding was specific. Furthermore, we also identified putative EBEs for the other two types of AvrXa27/TalAO-class TALEs in *Xa27* (Supplemental Figure 8) and verified them (Supplemental Figure 9).

Tal6b/AvrXa27A also bound to the 40-bp promoter fragment containing EBE_PthXo1_ (Xa13p) (Figure 4C and 4D). To determine the precise sequence recognized by Tal6b/AvrXa27A in the *OsSWEET11a* promoter, nine additional Cy5-labeled fragments (Xa13p-1–Xa13p-9) were synthesized based on the EBE prediction scores obtained with the TALE-NT program (Figure 3G, Supplemental Figure 10A). Surprisingly, only Xa13p-5 and Xa13p-9 bound the His-Tal6b/AvrXa27A protein (Supplemental Figure 10B–10F). Because no recognition score was generated by TALE-NT (Figure 3G), a 16-bp putative EBE (−246 to −231 bp) in the *OsSWEET11a* promoter was manually aligned with the 15.5 RVDs of Tal6b/AvrXa27A. To investigate whether the 16-bp sequence is the cognate EBE_Tal6b_ of *OsSWEET11a*, 4 additional 19-bp probes (Xa13p-10–Xa13p-13) were synthesized (Figure 4E). Xa13p-10 carried the aforementioned 16-bp EBE, and Xa13p-11, Xa13p-12, and Xa13p-13 contained 1-, 2-, and 3-bp mutations, respectively, compared with Xa13p-10 (Figure 4E). Interactions of the Xa13p-10 and Xa13p-11 probes with the His-Tal6b/AvrXa27A protein were observed in gel shift assays; however, binding was not observed with Xa13p-12 or Xa13p-13 (Figure 4F). These results indicated that the EBE_Tal6b_ interacts with Tal6b/AvrXa27A at the *OsSWEET11a* promoter.

EMSAs confirmed that other members of the AvrXa27/TalAO-class TALEs, including AvrXa27, Tal17/AvrXa27B, and Tal6c/AvrXa27C, do not bind to EBE_Tal6b_ in the *OsSWEET11a* promoter (Supplemental Figure 11). Given that there are only two RVD differences between AvrXa27 and Tal6b/AvrXa27A (Figure 2A), we wondered whether these two RVDs affect the binding of AvrXa27 to EBEtal6b in the *OsSWEET11a* promoter. We therefore mutated 2 nt in the *OsSWEET11a* promoter (Supplemental Figure 12A) to match the two polymorphic RVDs in AvrXa27 according to the TALE code (Boch et al., 2009; Moscou and Bogdanove, 2009) and used the resulting probe (Xa13p-14) for EMSA. The results indicated that AvrXa27 was still unable to bind the mutated promoter fragment of *OsSWEET11a* (Supplemental Figure 12B).

### *OsSWEET11a* promoter mutations confer Tal6b/AvrXa27A-dependent resistance to *Xoo*

**Figure 5:**
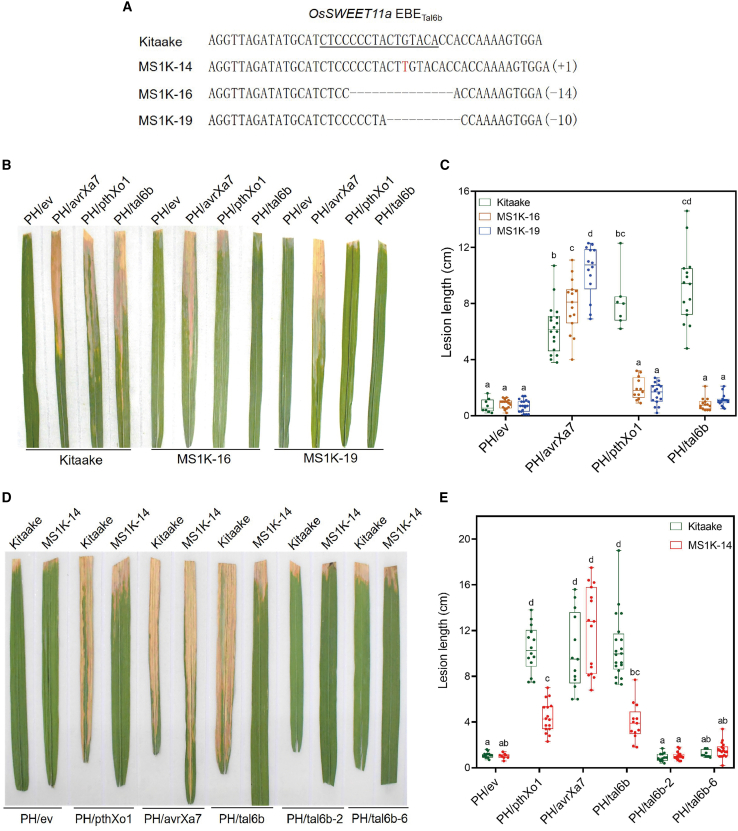
*OsSWEET11a* promoter mutations confer resistance to *Xoo*. **(A)** DNA sequence alignment of *OsSWEET11a* EBE_Tal6b_ alleles in wild-type Kitaake rice and derived mutants. **(B)** Disease symptoms on representative leaves of rice lines Kitaake, MS1K-16, and MS1K-19 after inoculation with *Xoo* strains PH/ev, PH/pthXo1, PH/avrXa7, and PH/tal6b. **(C)** Boxplots of mean lesion lengths (cm) on Kitaake, MS1K-16, and MS1K-19. **(D)** Disease symptoms on representative leaves of Kitaake and MS1K-14 rice after inoculation with *Xoo* strains PH/ev, PH/pthXo1, PH/avrXa7, PH/tal6b, PH/tal6b-2, and PH/tal6b-6. **(E)** Boxplots of mean lesion lengths (cm) on Kitaake and MS1K-14 rice. Lesions were measured at 14 dpi. Values with identical lowercase letters do not differ significantly at *P* < 0.05 according to ANOVA.

We further investigated the requirement for the *OsSWEET11a* EBE_Tal6b_ in disease susceptibility in the presence of Tal6b/AvrXa27A by generating mutations in the *OsSWEET11a* EBE using CRISPR technology. Three homozygous rice lines (T_2_ generation) were obtained in rice cv. Kitaake and named MS13K-14, MS13K-16, and MS13K-19. Rice line MS1K-14 carried a thymine insertion, whereas the other two lines carried 14- and 10-bp deletions, respectively (Figure 5A). The *Xoo* strains PH/ev, PH/avrXa7, PH/pthXo1, and PH/tal6b were used to evaluate EBE mutant lines for disease susceptibility. Based on lesion length, *Xoo* PH/ev was weakly virulent on Kitaake, MS13K-14, MS13K-16, and MS13K-19 rice (Figure 5B–5E). Inoculation with *Xoo* PH/pthXo1 and PH/tal6b resulted in long lesions on Kitaake but no disease lesions on MS13K-14, MS13K-16, and MS13K-19 (Figure 5B–5E), indicating that an intact EBE sequence in *OsSWEET11a* is required for Tal6b/AvrXa27A-mediated susceptibility.

## Discussion

In this study, we showed that Tal6b/AvrXa27A, an AvrXa27-like TALE in *Xoo*, functions as an avirulence factor to trigger *Xa27*-mediated resistance in rice. Tal6b/AvrXa27A was also shown to function as a virulence factor that targets the susceptibility gene *OsSWEET11a*. This new TALE targets *R* and *S* genes for BB by binding to the EBEs in the promoters of *Xa27* and *OsSWEET11a*, respectively.

Rice *Xa27* is an executor gene that is specifically activated by the cognate avirulence TALE AvrXa27; this TALE binds to the 16-bp EBE_AvrXa27_ sequence present in the *Xa27* promoter (Gu et al., 2005; Römer et al., 2009). In this study, we analyzed the molecular diversity of *avrXa27* and identified four variants of AvrXa27/TalAO-class TALEs in 37 different *Xoo* strains (Table 1). In addition to the parental TALE, AvrXa27, *Xoo* strains may produce AvrXa27A, AvrXa27B, or AvrXa27C; these also trigger *Xa27*-mediated ETI (Supplemental Figures 4 and 5) and contain several RVDs that differ from those in AvrXa27 (Table 1). These four AvrXa27/TalAO-class TALEs were not present in the genomes of 13 Asian *Xoo* strains or accessible African strains (Supplemental Table 2). The Asian strain AH28 was originally isolated from the Anhui province of China (Xu et al., 2022; Supplemental Table 2) and lacks TALEs of the AvrXa27/TalAO class (Supplemental Table 2). *Xoo* AH28 was virulent on rice cv. 78-15, which contains *Xa27* (Supplemental Figure 3), suggesting that *Xoo* can evolve to overcome *Xa27*-mediated resistance by disposing of the corresponding *avr* gene. Similarly, rice cv. IRBB27, which encodes *Xa27*, was susceptible to five *Xoo* strains (Gu et al., 2004) that may have also lost *avrXa27*-like genes.

*OsSWEET11a*, a member of the clade III sugar transporters in rice, is a known *S* gene targeted by the major TALE PthXo1 (Yang et al., 2006; Streubel et al., 2013). Our data showed that Tal6b/AvrXa27A is a novel major TALE that also activates *OsSWEET11a* expression (Figure 3E and 3F). Tal6b/AvrXa27A targets an EBE in *OsSWEET11a* that overlaps with the EBE of PthXo1 and harbors a completely different RVD array compared with that in PthXo1 (Figures 3G, 4E, and 4F). Similarly, the promoters of *OsSWEET14* and *OsTFX1* are targeted by several unrelated TALEs (Sugio et al., 2007; Antony et al., 2010; Yu et al., 2011; Streubel et al., 2013; Tran et al., 2018); thus, *OsSWEET11a* represents a new case of functional convergence in which two completely unrelated TALEs from different strains have evolved to bind overlapping EBEs.

It is widely accepted that plants mount a defense response against pathogens by tricking them into inducing resistance via *E* genes, which are dominant *R* genes (Hutin et al., 2015a; Timilsina et al., 2020). Four *E* genes, *Xa27*, *Xa10*, *Xa23*, and *Xa7*, have been cloned from rice (Gu et al., 2005; Tian et al., 2014; Wang et al., 2015; Chen et al., 2021; Luo et al., 2021). *Xa7* is activated by the TALEs AvrXa7 and PthXo3, which also target the rice susceptibility gene *OsSWEET14* (Chen et al., 2021; Luo et al., 2021). The *Xa7* EBE mimics the *OsSWEET14* EBE, which protects *OsSWEET14* against exploitation by *Xoo* by triggering cell death (Luo et al., 2021). In this study, we identified another TALE, Tal6b/AvrXa27A, that targets *E* and *S* genes in rice (Figure 4). Unlike those of AvrXa7 and PthXo3, the EBE of *Xa27* for Tal6b/AvrXa27A (EBE_AvrXa27A_) bears no obvious similarities to the corresponding EBE of *SWEET11a* (EBE_Tal6b_) (Figure 4), implying that the *Xa27* EBE may not be a mimic of the *OsSWEET11a* EBE. Expression analysis showed that transcription of *Xa27* is higher than expression of *OsSWEET11a* in rice cv. 78-15 when induced by Tal6b/AvrXa27A (Supplemental Figure 13). These results suggest that Tal6b/AvrXa27 has a greater capacity for inducing *Xa27* than *OsSWEET11a*, resulting in a disease-resistant phenotype. Because the co-evolution of AvrXa27/TalAO class TALE-*Xa27*-*OsSWEET11a* is not clear, further research is needed to confirm the hypothesis that *Xa27* evolved a promoter mimic that triggers a defense response and confounds the desired effects of the pathogen (Gu et al., 2005).

All four known types of AvrXa27/TalAO-class TALEs demonstrated binding to the respective EBEs in the *Xa27* promoter, triggering *Xa27*-mediated ETI, regardless of differences in their RVDs (Figures 2 and 5 and Supplemental Figures 4 and 9). However, among these TALEs, only Tal6b/AvrXa27A exhibited binding to EBE_Tal6b_ in the *OsSWEET11a* promoter and induced *OsSWEET11a-*mediated ETS (Figures 3 and 5 and Supplemental Figure 11). Tal6b/AvrXa27A shares only one to two RVD differences with other AvrXa27/TalAO-class TALEs (Table 1). The distinct functions displayed by Tal6b/AvrXa27A in activating both an *R* gene and an *S* gene indicate the critical role of these differences in determining specific target recognition and activation abilities. Interestingly, despite mutations of the nucleotides that correspond to the polymorphic RVDs between AvrXa27 and Tal6b/AvrXa27A, AvrXa27 still failed to bind to the mutated promoter fragment of *OsSWEET11a* (Supplemental Figure 12). This result suggests that observed differences in activity between AvrXa27 and Tal6b/AvrXa27A cannot be attributed solely to these specific nucleotide changes. Notably, the matches between the RVDs of Tal6b/AvrXa27A and EBE_Tal6b_ identified in our study do not strictly adhere to the TALE code, because only two RVD–nucleotide pairs exhibit the best match (Supplemental Figure 7). This finding implies the limitations of the current version of the TALE code. To gain a deeper understanding of these functional differences, further investigations, such as structural analyses and mutagenesis studies, are warranted to reveal the molecular basis for the unique properties of Tal6b/AvrXa27A. These studies will contribute to a comprehensive understanding of TALE-mediated host–pathogen interactions and the mechanisms that govern target recognition and activation.

**Figure 6:**
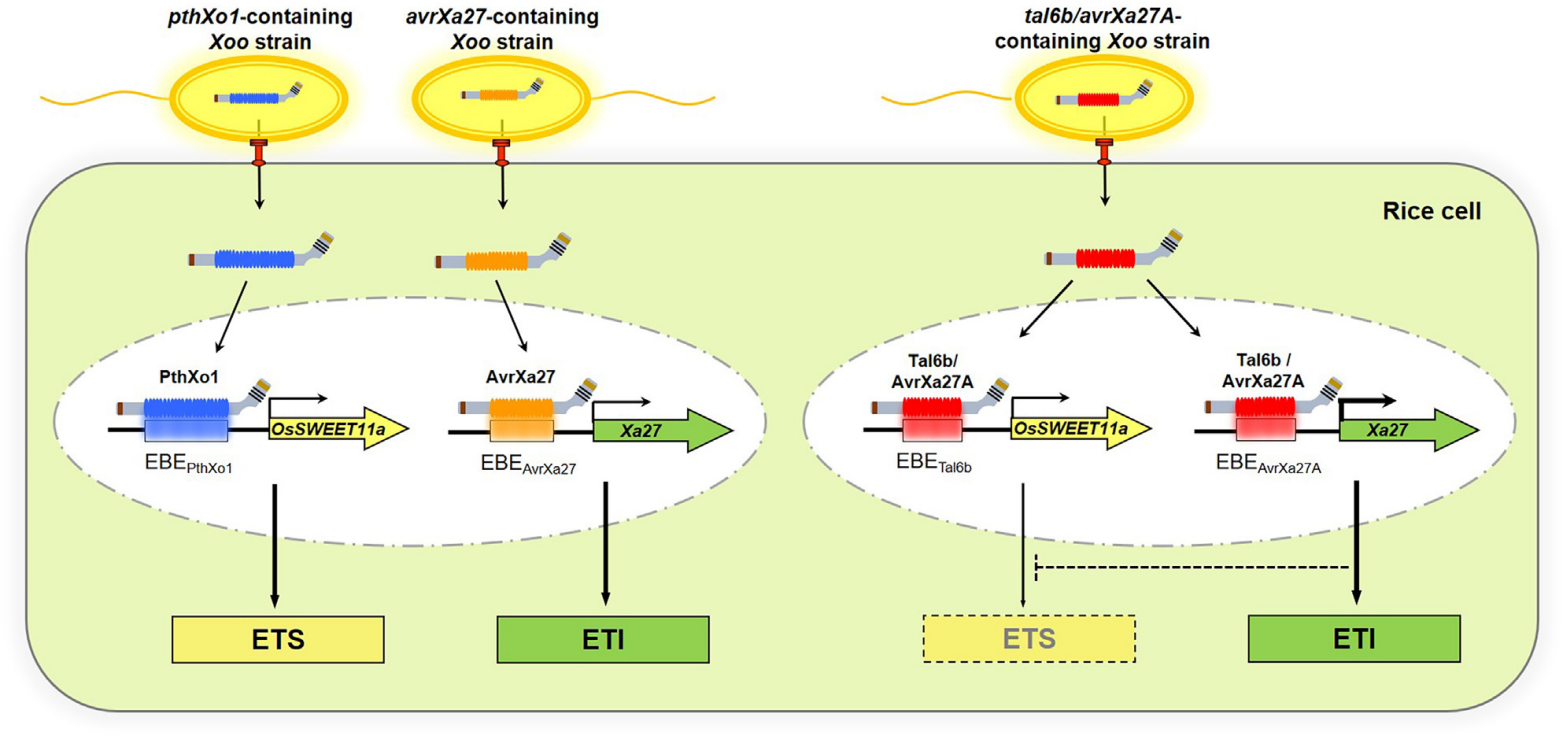
A working model of the role of Tal6b/AvrXa27A in the interaction between *Xoo* and rice. *pthXo1*-containing *Xoo* strains employ the major TALE PthXo1 to specifically target the *S* gene *OsSWEET11a*, leading to ETS in rice. *avrXa27*-containing *Xoo* strains induce *Xa27*-mediated resistance via AvrXa27 in rice cultivars that harbor *Xa27*. Conversely, *tal6b/avrXa27A*-containing *Xoo* strains secrete Tal6b/AvrXa27A, which targets *OsSWEET11a* and *Xa27.* This results in *Xa27*-mediated ETI that suppresses *OsSWEET11a*-mediated ETS.

Combining our data with those reported previously (Gu et al., 2005; Yang et al., 2006), we propose a working model for the roles of Tal6b/AvrXa27A in *Xoo* and rice (Figure 6). In this model, *Xoo* strains such as PXO99^A^ utilize the major TALE PthXo1 to target the *S* gene *OsSWEET11a*, which leads to ETS and virulence in rice. *Xoo* strains that harbor *avrXa27* (e.g., strain PXO86) induce *Xa27*-mediated resistance via AvrXa27 in rice cultivars that harbor *Xa27*. Finally, *tal6b/avrXa27A*-containing *Xoo* strains (e.g., strain LN18) secrete the Tal6b/AvrXa27A effector, which induces ETS by targeting *OsSWEET11a* in rice lines that lack *Xa27*. However, in rice lines that contain *Xa27*, Tal6b/AvrXa27A is recognized by *Xa27* and activates a high level of *Xa27* expression. This results in *Xa27*-mediated immunity that suppresses *OsSWEET11a*-mediated susceptibility (Figure 6). However, the mechanistic basis of *Xa27*-mediated ETI, *OsSWEET11a*-mediated ETS, and their crosstalk remains unclear and warrants further research.

This study and our previous findings (Xu et al., 2019) indicate that *Xoo* LN18 encodes three major TALEs (Tal5_LN18_, AvrXa7, and Tal6b/AvrXa27A), which target the *S* genes *OsSWEET13*, *OsSWEET14*, and *OsSWEET11a*, respectively. To our knowledge, this is the first example of a *Xoo* strain encoding three major TALEs that each target a known *SWEET* gene and may constitute a robust adaptation to overcome host loss-of-susceptibility recessive resistance due to *xa13*, *xa25*, and *xa41* (Yang et al., 2006; Hutin et al., 2015b; Zhou et al., 2015; Xu et al., 2019). Thus, *Xoo* LN18 may represent an emerging, highly virulent *Xoo* population that should be monitored by genome sequencing and TALome analysis. It is important to mention that rice lines with three edited *OsSWEET* EBEs showed resistance to *Xoo* LN18 (Xu et al., 2019), demonstrating that engineering resistance by exploiting EBEs in *OsSWEET* genes may be a promising approach for dealing with this rapidly evolving pathogen.

## Methods

### Plant materials, bacterial strains, and growth conditions

Rice cultivars IR24, Nipponbare, Kitaake, and 78-15 were grown in field plots and greenhouses at Shanghai Jiao Tong University (Shanghai, China). The bacterial strains used in this study are listed in Supplemental Table 1. *E. coli* strains were grown in Luria–Bertani medium at 37°C. *Xoo* strains were grown in nutrient broth (NB) or NB supplemented with 1.5% agar at 28°C. Antibiotics were used at the following concentrations when required: ampicillin (100 μg/ml), kanamycin (25 μg/ml), and spectinomycin (50 μg/ml).

### Pathogen inoculation assays

*Xoo* strains were cultured in NB supplemented with appropriate antibiotics at 28°C for 20 h. Bacterial suspensions (optical density 600 [OD_600_] = 0.8) were used to inoculate 2-month-old rice plants by the tip-cutting method. Disease symptoms were recorded at 14 dpi, and lesion lengths (cm) were measured. For observation of water soaking and HR, strains (OD_600_ = 0.6) were infiltrated into 2-week-old rice seedlings with needleless syringes, and symptoms were recorded 3 days after infiltration. Five leaves were inoculated with each *Xoo* strain, and experiments were repeated three times.

### Southern blotting

For *tal* gene detection, *Xoo* genomic DNA was extracted, digested with *Bam*HI, separated on agarose gels, and transferred to membranes for blotting as reported previously (Xu et al., 2019). The probe was made from a digoxigenin-labeled DNA fragment that contained the repetitive region of *avrXa27*. Bacterial genomic DNA was isolated with the Axygen Bacterial Genomic DNA Miniprep Kit. Restriction endonucleases and DNA molecular weight markers were provided by TaKaRa Bio (Japan). Digoxigenin-labeled Southern blotting kits were purchased from Roche (Switzerland), and Immobilon-Ny^+^ membranes were supplied by Millipore (USA).

### *tal* gene cloning and plasmid construction

*tal17* of *Xoo* HuN37 and *tal6c* of *Xoo* IXO221 were isolated as described previously (Xu et al., 2019). In brief, genomic DNA (2–3 μg) of HuN37 and IXO221 was digested with *Bam*HI (New England Biolabs, USA) and separated on 1.3% agarose gels. DNA fragments were excised, ligated into *Bam*HI-digested pBluescript II KS(−), and transferred into *E. coli* DH5α. The resulting plasmid library was screened for TALE-containing clones by *in situ* colony blot hybridization using the 3.2-kb *Sph*I fragment of *avrXa27* as a probe. Hybridizing colonies were further evaluated by PCR with the *tal*-specific primers TALN18-F and TALN18-R (Supplemental Table 3). Putative *tal*-containing clones were confirmed by restriction enzyme digestion and Sanger sequencing. *tal17* and *tal6c* were cloned into the pZW vector in frame with C-terminal FLAG tag epitopes, resulting in pZW-tal17 and pZW-tal6c (Supplemental Table 1). These plasmids were ligated into the broad-host-range vector pHM1 at the *Hin*dIII site, generating pHZW-tal17 and pHZW-tal6c (Supplemental Table 1), which were then transferred into a *tal*-free PH strain (derived from PXO99^A^) (Ji et al., 2016) to obtain PH/tal17 and PH/tal6c (Supplemental Table 1).

To construct deletions in the CRR, pZW-tal6b was first completely digested with *Aat*II and then partially digested with *Msc*I; fragments from 250–1600 bp were inserted into *Aat*II-*Msc*I-digested pZW-tal6b. Single colonies were selected and sequenced to confirm the size of repeat regions, and this resulted in pZW-tal6b-2 and pZW-tal6b-6 (Supplemental Table 1). The plasmids were also inserted into pHM1 to generate pHZW-tal6b-2 and pHZW-tal6b-6 (Supplemental Table 1) and transferred into the PH strain to obtain PH/tal6b-2 and PH/tal6b-6 (Supplemental Table 1).

### qRT–PCR

Total RNA was isolated from inoculated plants 24 h post-inoculation using RNAiso Plus reagent (TaKaRa Bio). Trace amounts of genomic DNA were removed with RNase-free DNase I (TransGen, China) prior to synthesis of cDNA. First-strand cDNA was diluted to a final volume of 20 μL, and SYBR Green-labeled PCR fragments were amplified using the *Xa27* gene-specific primers Xa27-qF and Xa27-qR (Supplemental Table 3) and the 7500 Real-Time PCR System (Applied Biosystems, USA). The rice *Actin* gene was used as an internal control (primers Actin-F and Actin-R; Supplemental Table 3). The comparative threshold (2^−ΔΔCt^) method was used to calculate relative mRNA levels. qRT–PCR experiments were performed in triplicate.

### Western blots

TALE proteins were detected in *Xoo* strains by immunoblotting as described previously (Xu et al., 2019). In brief, *Xoo* strains were cultured in NB to the logarithmic phase and harvested by centrifugation. Bacterial cells were washed twice, and the concentration was adjusted to OD_600_ = 2.0 with sterile distilled water. SDS loading buffer (5×) was added to the bacterial suspensions; these were then boiled in a water bath for 10 min, separated on SDS–PAGE gels, and transferred to polyvinylidene difluoride membranes for immunoblotting with anti-FLAG (TransGen) as the primary antibody. Goat anti-rabbit immunoglobulin G (TransGen) was used for detection of primary antibodies with the EasySee Western Kit supplied by TransGen.

### GUS assay

GUS measurements were carried out as described previously (Haq et al., 2022). Promoter fragments of *OsSWEET11a* and *Xa27* were cloned into the binary GUS reporter construct pCAMBIA1381 using primers listed in Supplemental Table 3. The binary vector pHB was used to clone and express TALEs PthXo1 and Tal6b in *N. benthamiana*. The effector and reporter constructs (Supplemental Table 1) were transformed into *Agrobacterium* strain EHA105 by the freeze–thaw method and then co-expressed (OD_600_ = 1.0 for each strain) in 5- to 7-week-old *N. benthamiana* leaves via *Agrobacterium*-mediated transformation. Three leaf discs (1 cm diameter) were collected 48 h post inoculation, and GUS activity was measured using 4-methylumbelliferyl-β-glucuronide. Proteins were quantified using a Bradford Protein Quantification Kit (Yeasen, Shanghai).

### EMSAs

*avrXa27*, *tal6b*, *tal17*, and *tal6c* were cloned into the pET-30a vector with a His tag to construct the plasmids pET30a-avrXa27, pET30a-tal6b/avrXa27A, pET30a-tal17/avrXa27B, and pET30a-tal6c/avrXa27C (Supplemental Table 1). After induction with isopropyl-β-D-thiogalactopyranoside (IPTG), the fusion proteins His-AvrXa27, His-Tal6b/AvrXa27A, His-AvrXa27B, and His-AvrXa27C were produced in *E. coli* BL21(DE3) cells containing pET30a-avrXa27, pET30a-tal6b/avrXa27A, pET30a-avrXa27B, and pET30a-avrXa27C, respectively. Ni-NTA HisBind Resin (Novagen, USA) was used to purify the proteins according to the manufacturer’s manual. Purified His-AvrXa27, His-Tal6b/AvrXa27A, His-AvrXa27B, and His-AvrXa27C were mixed with Cy5-labeled *Xa27* or *OsSWEET11a* promoter fragments (probes synthesized by Shanghai DNA Bioscience) and loaded onto 4.5% nondenaturing polyacrylamide gels for electrophoretic separation. An Amersham Typhoon RGB Biomolecular Imager (Cytiva, Sweden) was used to scan the fluorescence patterns in the gels to detect the Cy5 fluorophore. Three independent experiments were performed with similar results.

### Generation of transgenic constructs and transgenic plants

Kitaake rice was genetically modified with CRISPR–Cas9 technology as described previously (Xu et al., 2019). In brief, target sequences were selected within the promoter regions of *OsSWEET11a*, and single-guide RNAs (sgRNAs) were designed with the CRISPR MultiTargeter program (http://www.multicrispr.net/index.html) and then synthesized. BLAST searches (http://blast.ncbi.nlm.nih.gov/Blast.cgi) of the target sequences were performed against rice genome sequences to confirm target specificity. The sgRNA and Cas9 constructs were transferred into calli of rice cv. Kitaake by *Agrobacterium*-mediated transformation (Biorun, Wuhan, China). Genomic DNA was isolated from leaves of transgenic rice using the cetyltrimethyl ammonium bromide (CTAB) method and used for PCR amplification of the target regions with specific primers (Supplemental Table 3). The resulting amplicons were subjected to Sanger sequencing.

### Data analysis

The newly isolated *tal* gene sequences from *Xoo* strains in this report were used as queries in BLAST searches against the NCBI database (https://www.ncbi.nlm.nih.gov/) to identify homologous sequences. *tal* gene sequences were aligned with BioEdit software (Alzohairy, 2011). The amino acid and RVD sequences of TALEs were analyzed using SnapGene software (www.snapgene.com). The EBEs recognized by AvrXa27/TalAO-class TALEs were predicted in the promoters of *Xa27* and *OsSWEET11a* using the TALgetter tool (Grau et al., 2013) and TALE-NT (Doyle et al., 2012).

## Funding

This work was supported by the 10.13039/501100001809National Natural Science Foundation of China (31830072 to G.Chen, 32102147 to Z.X., and 32202243 to X.X.), the 10.13039/501100002858China Postdoctoral Science Foundation (2020M681309 to Z.X. and 2021M702156 to X.X.), the Shanghai Postdoctoral Excellence Program (2020277 to Z.X. and 2021229 to X.X.), and the International Postdoctoral Exchange Fellowship Program (PC2021043).

## Author contributions

Z.X., X.X., Y.L., L.L., Q.W., and Yijie Wang performed the experiments. Z.X. analyzed the data. Yong Wang, J.Y., and G.Cheng contributed materials. Z.X., B.Z., L.Z., and G.Chen planned and designed the research. Z.X. and G.Chen wrote an initial version of the manuscript, which was read and revised by all authors.

## Supplemental information

Document S1. Supplemental Figures 1–14 and Supplemental Tables 1–3

Document S2. Article plus supplemental information

## References

[bib1] Alzohairy A.M. (2011). Bioedit: an important software for molecular biology. GERF Bull. Biosci.

[bib2] Antony G., Zhou J., Huang S., Li T., Liu B., White F., Yang B. (2010). Rice *xa13* recessive resistance to bacterial blight is defeated by induction of the disease susceptibility gene Os-*11N3*. Plant Cell.

[bib4] Boch J., Scholze H., Schornack S., Landgraf A., Hahn S., Kay S., Lahaye T., Nickstadt A., Bonas U. (2009). Breaking the code of DNA binding specificity of TAL-type III effectors. Science.

[bib5] Boch J., Bonas U. (2010). *Xanthomonas* AvrBs3 family-type III effectors: discovery and function. Annu. Rev. Phytopathol..

[bib6] Boch J., Bonas U., Lahaye T. (2014). TAL effectors-pathogen strategies and plant resistance engineering. New Phytol..

[bib8] Chen X., Liu P., Mei L., He X., Chen L., Liu H., Shen S., Ji Z., Zheng X., Zhang Y. (2021). *Xa7*, a new executor R gene that confers durable and broad-spectrum resistance to bacterial blight disease in rice. Plant Comm..

[bib9] Doyle E.L., Booher N.J., Standage D.S. (2012). TAL Effector-Nucleotide Targeter (TALE-NT) 2.0: tools for TAL effector design and target prediction. Nucleic Acids Res..

[bib10] Gu K., Tian D., Yang F. (2004). High-resolution genetic mapping of *Xa27(t)*, a new bacterial blight resistance gene in rice, *Oryza sativa* L. Theor. Appl. Genet..

[bib11] Gu K., Yang B., Tian D., Wu L., Wang D., Sreekala C., Yang F., Chu Z., Wang G.L., White F.F. (2005). *R* gene expression induced by a type-III effector triggers disease resistance in rice. Nature.

[bib12] Grau J., Wolf A., Reschke M., Bonas U., Posch S., Boch J. (2013). Computational predictions provide insights into the biology of TAL effector target sites. PLoS Comput. Biol..

[bib13] Haq F., Xu X., Ma W., Shah S.M.A., Liu L., Zhu B., Zou L., Chen G. (2022). A *Xanthomonas* transcription activator-like effector is trapped in nonhost plants for immunity. Plant Comm..

[bib14] Hutin M., Pérez-Quintero A.L., Lopez C., Szurek B. (2015). MorTAL Kombat: the story of defense against TAL effectors through loss-of-susceptibility. Front. Plant Sci..

[bib15] Hutin M., Sabot F., Ghesquière A., Koebnik R., Szurek B. (2015). A knowledge-based molecular screen uncovers a broad-spectrum *OsSWEET14* resistance allele to bacterial blight from wild rice. Plant J..

[bib16] Ji Z., Ji C., Liu B., Zou L., Chen G., Yang B. (2016). Interfering TAL effectors of *Xanthomonas oryzae* neutralize *R*-gene-mediated plant disease resistance. Nat. Commun..

[bib17] Ji Z., Guo W., Chen X., Wang C., Zhao K. (2022). Plant executor genes. Int. J. Mol. Sci..

[bib18] Jiang N., Yan J., Liang Y., Shi Y., He Z., Wu Y., Zeng Q., Liu X., Peng J. (2020). Resistance genes and their interactions with bacterial blight/leaf streak pathogens (*Xanthomonas oryzae*) in rice (*Oryza sativa* L.)-an updated review. Rice.

[bib19] Jones J.D.G., Dangl J.L. (2006). The plant immune system. Nature.

[bib20] Koseoglou E., van der Wolf J.M., Visser R.G.F., Bai Y. (2022). Susceptibility reversed: modified plant susceptibility genes for resistance to bacteria. Trends Plant Sci..

[bib21] Luo D., Huguet-Tapia J.C., Raborn R.T., White F.F., Brendel V.P., Yang B. (2021). The *Xa7* resistance gene guards the rice susceptibility gene *SWEET14* against exploitation by the bacterial blight pathogen. Plant Comm..

[bib22] Mücke S., Reschke M., Erkes A. (2019). Transcriptional reprogramming of rice cells by *Xanthomonas oryzae* TALEs. Front. Plant Sci..

[bib23] Moscou M.J., Bogdanove A.J. (2009). A simple cipher governs DNA recognition by TAL effectors. Science.

[bib24] Niño-Liu D.O., Ronald P.C., Bogdanove A.J. (2006). *Xanthomonas oryzae* pathovars: model pathogens of a model crop. Mol. Plant Pathol..

[bib52] Oliva R., Ji C., Atienza-Grande G. (2019). Broad-spectrum resistance to bacterial blight in rice using genome editing. Nat. Biotechnol..

[bib25] Römer P., Recht S., Lahaye T. (2009). A single plant resistance gene promoter engineered to recognize multiple TAL effectors from disparate pathogens. Proc. Natl. Acad. Sci. USA.

[bib26] Römer P., Recht S., Strauß T. (2010). Promoter elements of rice susceptibility genes are bound and activated by specific TAL effectors from the bacterial blight pathogen, *Xanthomonas oryzae* pv*. oryzae*. New Phytol..

[bib27] Salzberg S.L., Sommer D.D., Schatz M.C. (2008). Genome sequence and rapid evolution of the rice pathogen *Xanthomonas oryzae* pv. *oryzae* PXO99^A^. BMC Genom..

[bib28] Streubel J., Pesce C., Hutin M., Koebnik R., Boch J., Szurek B. (2013). Five phylogenetically close rice *SWEET* genes confer TAL effector-mediated susceptibility to *Xanthomonas oryzae* pv. *oryzae*. New Phytol..

[bib50] Sugio A., Yang B., Zhu T., White F.F. (2007). Two type III effector genes of *Xanthomonas oryzae* pv. *oryzae* control the induction of the host genes *OsTFIIAγ1* and *OsTFX1* during bacterial blight of rice. Proc. Natl. Acad. Sci. USA.

[bib29] Tian D., Wang J., Zeng X., Gu K., Qiu C., Yang X., Zhou Z., Goh M., Luo Y., Murata-Hori M. (2014). The rice TAL effector-dependent resistance protein XA10 triggers cell death and calcium depletion in the endoplasmic reticulum. Plant Cell.

[bib30] Tran T.T., Pérez-Quintero A.L., Wonni I. (2018). Functional analysis of African *Xanthomonas oryzae* pv. *oryzae* TALomes reveals a new susceptibility gene in bacterial leaf blight of rice. PLoS Pathog..

[bib31] Timilsina S., Potnis N., Newberry E.A. (2020). *Xanthomonas* diversity, virulence and plant–pathogen interactions. Nat. Rev. Microbiol..

[bib32] Wang C., Zhang X., Fan Y., Gao Y., Zhu Q., Zheng C., Qin T., Li Y., Che J., Zhang M. (2015). XA23 is an executor R protein and confers broad-spectrum disease resistance in rice. Mol. Plant.

[bib33] White F.F., Yang B. (2009). Host and pathogen factors controlling the rice-*Xanthomonas oryzae* interaction. Plant Physiol..

[bib34] Wu L.B., Eom J.S., Isoda R. (2022). *OsSWEET11b*, a potential sixth leaf blight susceptibility gene involved in sugar transport-dependent male fertility. New Phytol..

[bib51] Wu L., Goh M.L., Sreekala C., Yin Z. (2008). XA27 depends on an amino-terminal signal-anchor-like sequence to localize to the apoplast for resistance to *Xanthomonas oryzae* pv *oryzae*. Plant Physiol..

[bib35] Xing J., Zhang D., Yin F. (2021). Identification and fine-mapping of a new bacterial blight resistance gene, *Xa47(t)*, in G252, an introgression line of Yuanjiang common wild rice (*Oryza rufipogon*). Plant Dis..

[bib37] Xu Z., Xu X., Gong Q., Li Z., Li Y., Wang S., Yang Y., Ma W., Liu L., Zhu B. (2019). Engineering broad-spectrum bacterial blight resistance by simultaneously disrupting variable TALE-binding elements of multiple susceptibility genes in rice. Mol. Plant.

[bib38] Xu Z., Wang S., Liu L. (2020). Genome resource of a hypervirulent strain LN4 of *Xanthomonas oryzae* pv. *oryzae* causing bacterial blight of rice. Plant Dis..

[bib40] Xu Z., Xu X., Wang Y. (2022). A varied AvrXa23-like TALE enables the bacterial blight pathogen to avoid being trapped by *Xa23* resistance gene in rice. J. Adv. Res..

[bib36] Xu Z., Zou L., Ma W., Cai L., Yang Y., Chen G. (2017). Action modes of transcription activator-like effectors (TALEs) of *Xanthomonas* in plants. J. Integr. Agric..

[bib41] Yang B., White F.F. (2004). Diverse members of the AvrBs3/PthA family of type III effectors are major virulence determinants in bacterial blight disease of rice. Mol. Plant Microbe Interact.

[bib42] Yang B., Sugio A., White F.F. (2006). *Os8N3* is a host disease-susceptibility gene for bacterial blight of rice. Proc. Natl. Acad. Sci. USA.

[bib43] Yu Y., Streubel J., Balzergue S. (2011). Colonization of rice leaf blades by an African strain of *Xanthomonas oryzae* pv. *oryzae* depends on a new TAL effector that induces the rice Nodulin-3 *Os11N3* gene. Mol. Plant Microbe Interact.

[bib44] Zhang J., Yin Z., White F. (2015). TAL effectors and the executor *R* genes. Front. Plant Sci..

[bib45] Zhang B., Han X., Yuan W., Zhang H. (2022). TALEs as double-edged swords in plant–pathogen interactions: Progress, challenges, and perspectives. Plant Comm..

[bib46] Zhao K., Zhang Q. (2021). A climate-resilient *R* gene in rice traps two pathogen effectors for broad and durable resistance to bacterial blight. Mol. Plant.

[bib47] Zhou J., Peng Z., Long J. (2015). Gene targeting by the TAL effector PthXo2 reveals cryptic resistance gene for bacterial blight of rice. Plant J..

[bib48] Zhou J.M., Zhang Y. (2020). Plant immunity: danger perception and signaling. Cell.

