# Supplementary material for: Tal6b/AvrXa27A, a hidden TALE targeting the susceptibility gene OsSWEET11a and the resistance gene Xa27 in rice
Source: Plant Commun. 2023 Sep 20;5(2):100721. doi: 10.1016/j.xplc.2023.100721 (PMC10873877; doi:10.1016/j.xplc.2023.100721)
Supplement: Document S1. Supplemental Figures 1–14 and Supplemental Tables 1–3 [file mmc1.pdf]

Plant Communications, Volume *5*

Supplemental information

Tal6b/AvrXa27A, a hidden TALE targeting the susceptibility gene *OsSWEET11a* and the resistance gene *Xa27* in rice

Zhengyin Xu, Xiameng Xu, Ying Li, Linlin Liu, Qi Wang, Yijie Wang, Yong Wang, Jiali Yan, Guanyun Cheng, Lifang Zou, Bo Zhu, and Gongyou Chen

Supplemental Information

Tal6b/AvrXa27A, a Hidden TALE Targeting both the Susceptibility Gene *OsSWEET11a* and the Resistance Gene *Xa27* in Rice

Zhengyin Xu[1†], Xiameng Xu[1†], Ying Li[1], Linlin Liu[1], Qi Wang[1], Yijie Wang[1], Yong Wang[1], Jiali Yan[1], Guanyun Cheng[1], Lifang Zou[1], Bo Zhu[1], and Gongyou Chen[1,2*]

[1]Shanghai Collaborative Innovation Center of Agri-Seeds, School of Agriculture and Biology, Shanghai Jiao Tong University, Shanghai, 200240, China

[2]State Key Laboratory of Microbial Metabolism, Shanghai Jiao Tong University, Shanghai, 200240, China

[*] Correspondence: Gongyou Chen (gyouchen@sjtu.edu.cn).

[†] These authors contributed equally to this work.

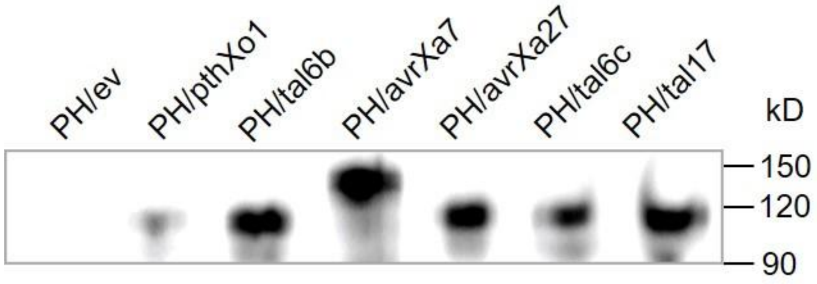

Figure S1 Western blot analysis of TALE proteins in *Xoo* PH derivatives, PH/ev, PH/pthXo1, PH/tal6b, PH/avrXa7, PH/avrXa27, PH/tal6c and PH/tal17. TALE proteins were detected using anti-FLAG as the primary antibody.

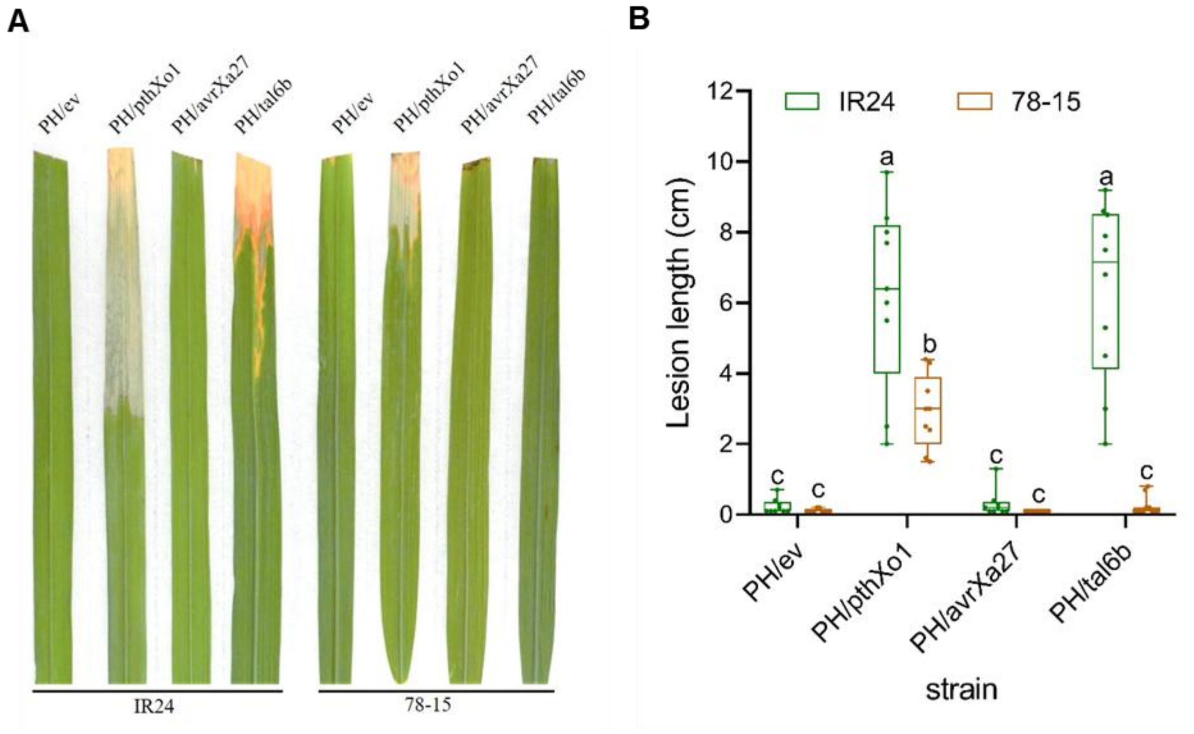

Figure S2 Disease symptoms on rice cultivars IR24 and 78-15. (A) Symptoms on rice leaves inoculated with *Xoo* PH derivatives containing the empty vector (ev), *pthXo1*, *avrXa27*, and *tal6b*. The images were taken at 14 days post-inoculation (dpi). (B) Box plots of mean disease lesion lengths (cm) on cvs. IR24 and 78-15. Lesions were measured 14 dpi; dots denote individual observations from at least five inoculated leaves, and whiskers display the first and third quartiles, split by the median. Values with the same lowercase letters do not differ significantly at $P < 0.05$ based on ANOVA.

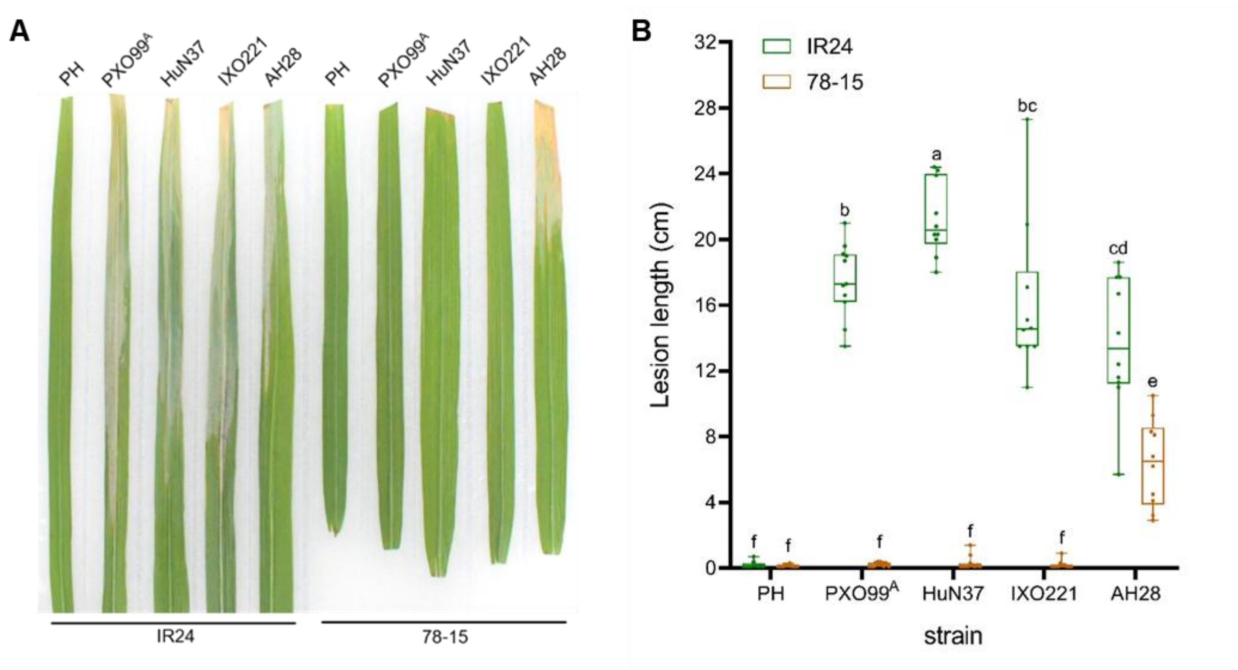

Figure S3 Symptoms on rice cultivars IR24 and 78-15 after inoculation with *Xoo* strains containing AvrXa27 orthologues. (A) Symptoms on rice leaves inoculated with *Xoo* PH (*tal*-free strain), PXO99^{A} (encodes *avrXa27*), HuN37 (*avrXa27B*), IXO221(*avrXa27C*), and AH28. (B) Box plots of mean disease lesion lengths (cm) on cvs. IR24 and 78-15. Values with the same lowercase letters do not differ significantly at $P < 0.05$ based on ANOVA.

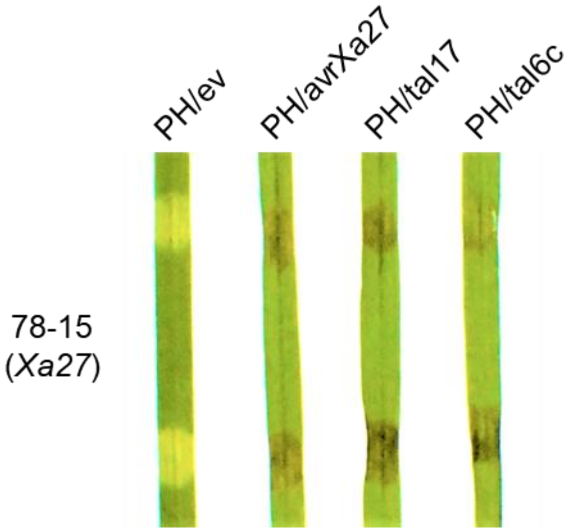

Figure S4 Rice leaves of cv. 78-15 (*Xa27*) inoculated with *Xoo* PH derivatives, PH/ev, PH/avrXa27, PH/tal17, and PH/tal6c.

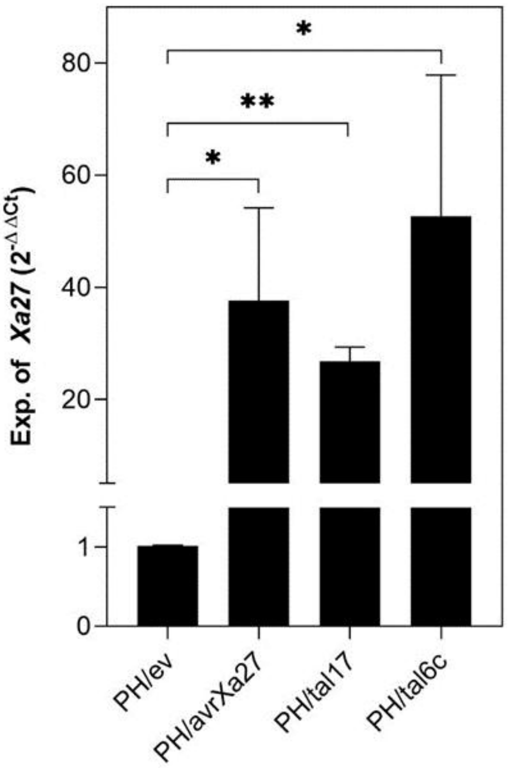

Figure S5 Expression of *Xa27* in 78-15 rice leaves inoculated *Xoo* PH derivatives, PH/ev, PH/avrXa27, PH/tal17, and PH/tal6c. Asterisks represent significant difference determined by Student's t-test (*P < 0.05, **P < 0.01).

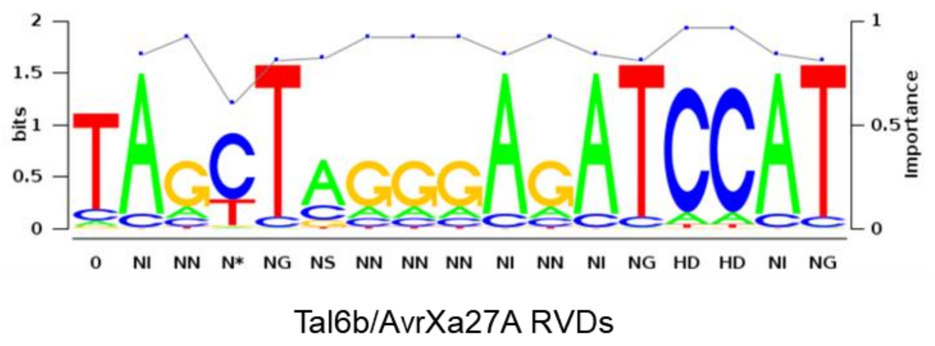

Figure S6 The predicted theoretical EBE of Tal6b/AvrXa27A RVDs. The Tal6b/AvrXa27A RVDs and their associated nucleotides are shown. The logo was produced using TALgetter (Galaxy v.1.1 http://galaxy.informatik.uni-halle.de/).

	0	1	2	3	4	5	6	7	8	9	10	11	12	13	14	15	16	17	Score
Xa27	T	A	G	A	A	G	A	A	G	A	G	A	C	C	C	A	T	A	
AvrXa27		NI	NN	N*	NG	NS	NN	NN	NN	NI	NN	NI	N*	HD	HD	NI	NG	NG	15.64
AvrXa27A		NI	NN	N*	NG	NS	NN	NN	NN	NI	NN	NI	NG	HD	HD	NI	NG		14.99
AvrXa27B		NI	NN	N*	NG	NS	NN	NN	NN	NN	NN	NI	NG	HD	HD	NI	NG		15.85
AvrXa27C		NI	NN	N*	NG	NS	NN	NN	NN	NI	NN	NI	NG	HD	HD	NI	HG	N*	17.62

Figure S7 AvrXa27/TalAO class TALEs are predicted to target EBEs in *Xa27*. EBE prediction scores were generated by TALE-NT, and lower scores indicate higher binding affinity between the RVDs and the target sequence.

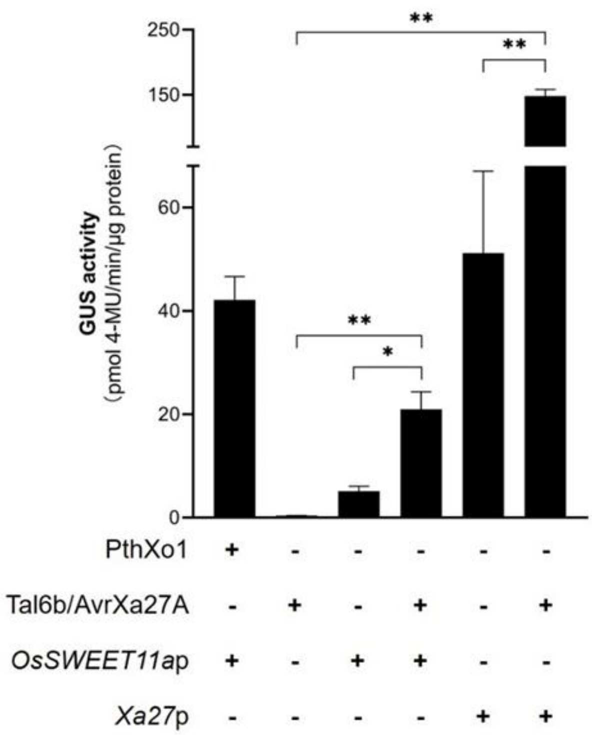

Figure S8 GUS assays show Tal6b/AvrXa27A inducibility of the *OsSWEET11a* (*OsSWEET11a*p) and *Xa27* promoter (*Xa27*p) in *Nicotiana benthamiana*. The TALE PthXo1 and the *OsSWEET11a* promoter were used as a positive control. Samples were collected at 48 hpi, and GUS activity was calculated. Error bars indicate means ±SD (n = 3). Asterisks represent significant difference determined by Student's t-test (*P < 0.05, **P < 0.01). 4-MU, 4-methyl-umbelliferone.

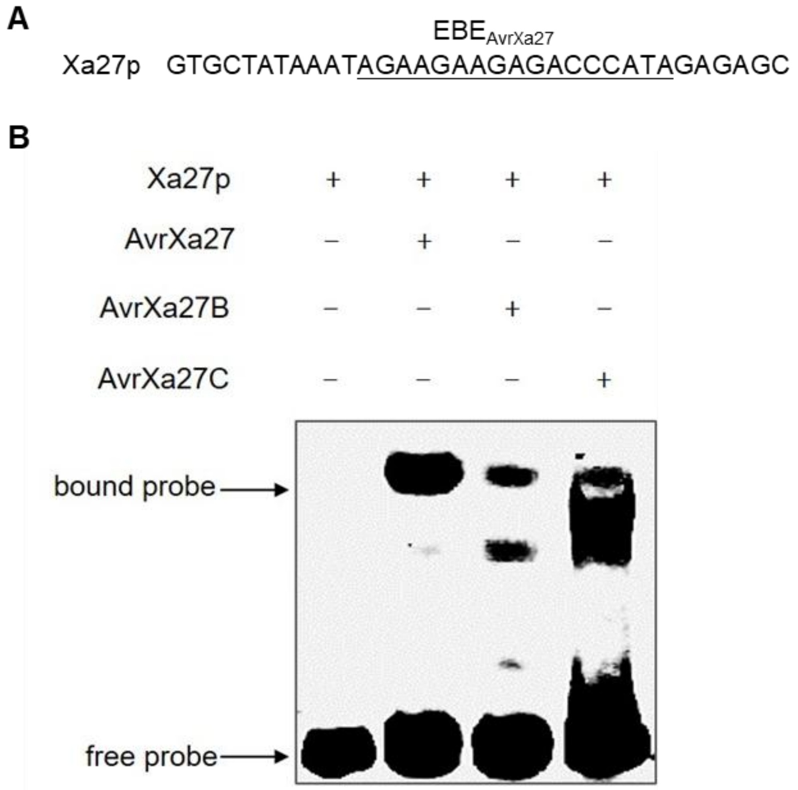

Figure S9 AvrXa27/TalAO class TALEs bind the EBEs of *Xa27*. (**A**) Nucleotide sequence of the *Xa27* promoter fragment (probe); $EBE_{AvrXa27}$ is underscored. (**B**) EMSA with His-AvrXa27, His-Tal17/AvrXa27B, and His-Tal6c/AvrXa27C fusion proteins and a Cy5-labeled *Xa27* promoter fragment (Xa27p). Positions of the bound and free probe are indicated on the left.

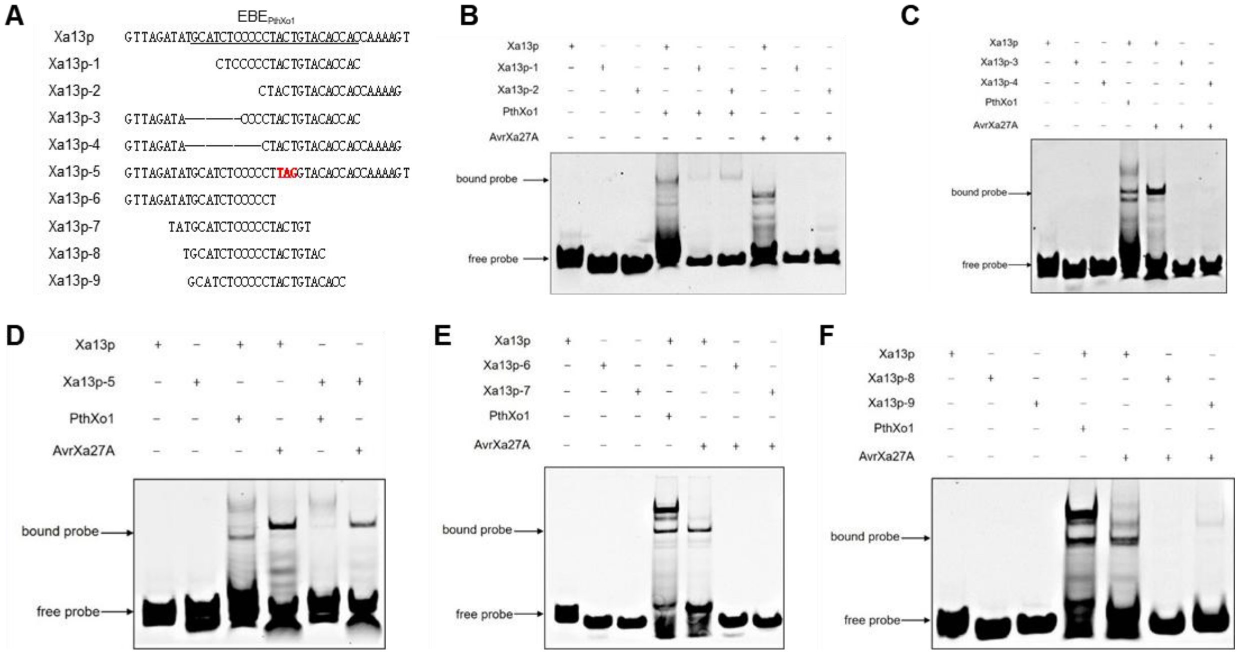

Figure S10. Tal6b/AvrXa27A does not bind to the six predicted EBEs of the *OsSWEET11a* promoter in gel shift assays. (**A**) Alignment of Xa13p, Xa13p-1, Xa13p-2, Xa13p-3, Xa13p-4, Xa13p-5, Xa13p-6, Xa13p-7, Xa13p-8, and Xa13p-9 sequences; EBE_{PthXo1} is underscored. Mutations in the *OsSWEET11a* promoter sequence are indicated in red font. (**B-F**) His-tagged PthXo1 and Tal6b/AvrXa27A interact with ten probes of the *OsSWEET11a* promoter in gel shift assays. Positions of the bound and free probe are indicated at the left.

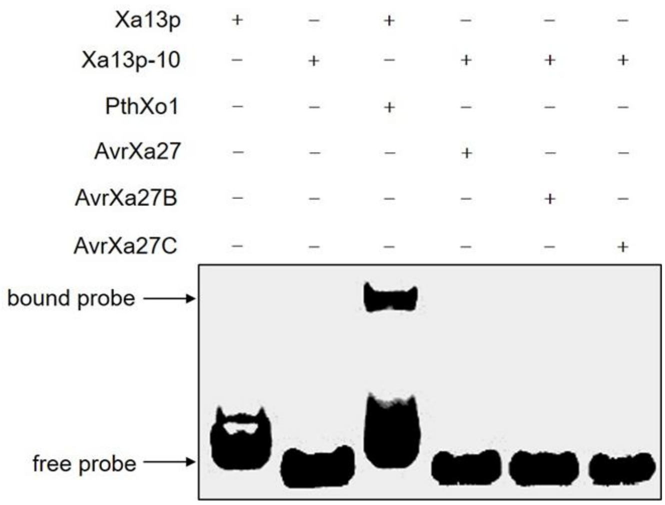

Figure S11 Other types of AvrXa27/TalAO class TALEs do not bind to the EBE_{Tal6b} of *OsSWEET11a*. EMSA with His-AvrXa27, His-Tal17/AvrXa27B, and His-Tal6c/AvrXa27C fusion proteins and a Cy5-labeled *OsSWEET11a* promoter fragment (Xa13p-10). Positions of the bound and free probe are indicated on the left.

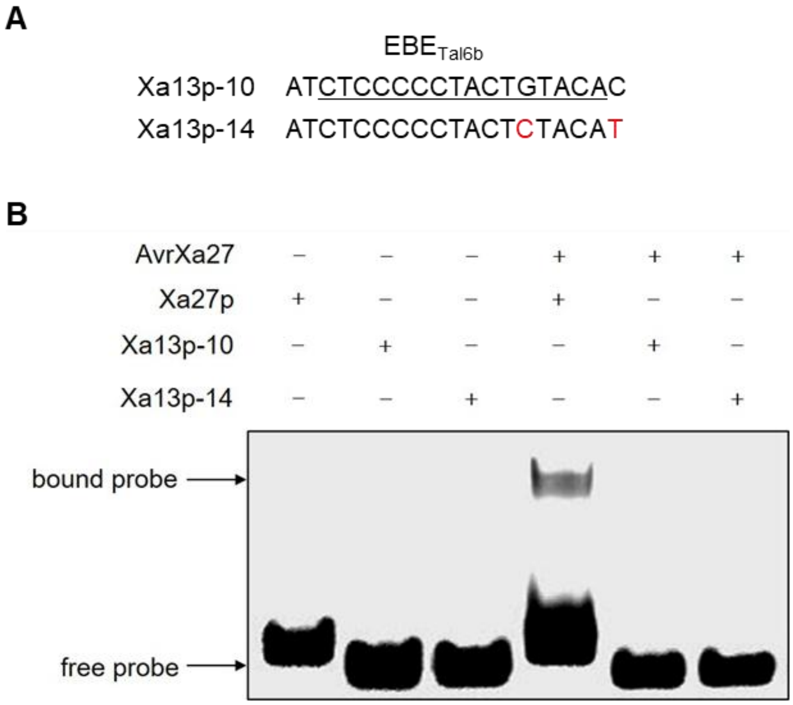

Figure S12 AvrXa27 does not bind to the mutated EBE_{Tal6b} of *OsSWEET11a*. (**A**) Alignment of Xa13p-10 and Xa13p-14 sequences. The EBE_{Tal6b} is underlined. Mutations in the *OsSWEET11a* promoter sequence are indicated in red font. (**B**) EMSA with His-AvrXa27 fusion protein and Xa13p-10 or Xa13p-14. Positions of the bound and free probe are indicated on the left.

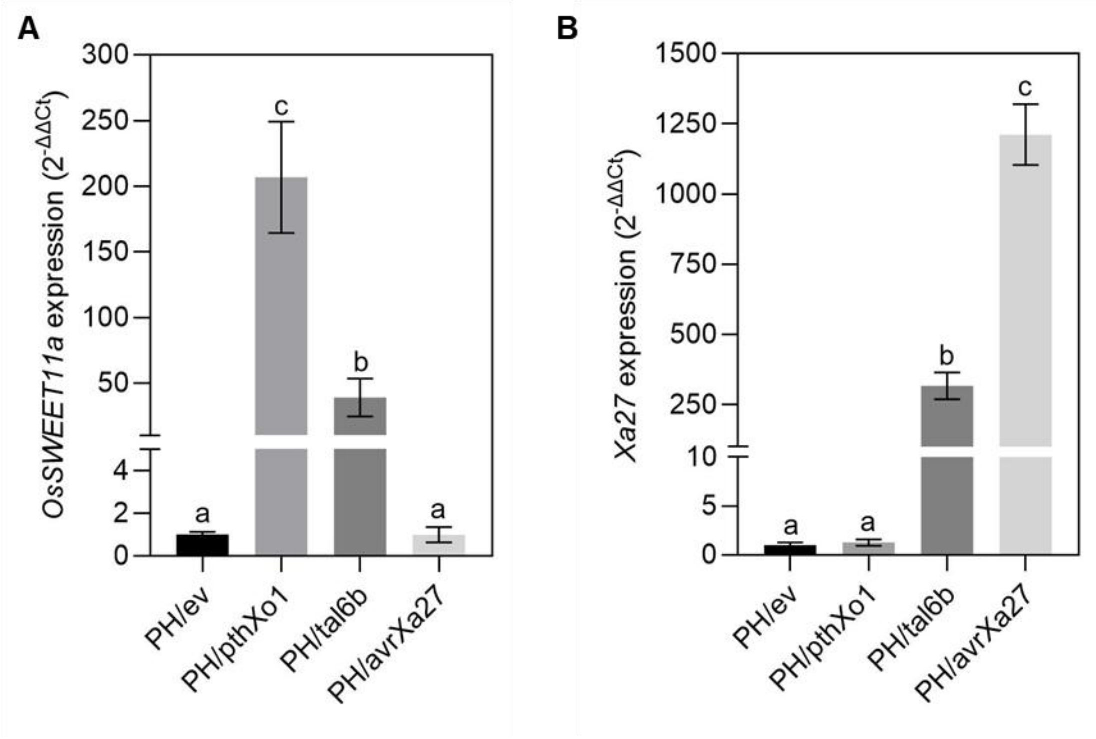

Figure S13. Expression of *OsSWEET11a* and *Xa27* in 78-15 rice leaves inoculated with *Xoo* strains. The expression of *OsSWEET11a* **(A)** and *Xa27* **(B)** was measured in rice leaves infiltrated with *Xoo* PH/ev, PH/pthXo1, PH/tal6b or PH/avrXa27. RNA was extracted from leaves 24 hpi and used for qRT-PCR with *OsSWEET11a* and *Xa27*-specific primers. The expression level of *Actin* was used as an internal control. Values with the same lowercase letters do not differ significantly at $P < 0.05$ based on ANOVA.

>*tal6b* (3312 bp)

ATGGATCCCATTCGTTCGCGCACGCCAAGTCCTGCCCGCGAGCTTCTGCCCGGACCCCAACCGGATAGGGTT
CAGCCGACTGCAGATCGGGGGggggCTCCGCCTGCTGGCGGCCCCcTGGATGGCTTGCCCGCTCGGCGGACGA
TGTCCCGGACCCGGCTGCCATCTCCCCcTGCGCCCTCGCCTGCGTTCTCGGCGGGCAGCTTCAACGATCTGCT
CCGTCAGTTCGATCCGTCGCTTCTTGATACATCGCTTCTTGATTCGATGCCTGCCGTCGGCACGCCGCATACA
GCGGCTGCCCCAGCAGAGTGGGATGAGGTGCAATCGGGTCTGCGTGCAGCCGATGACCCGCCACCCACCGT
GCGTGTCGCTGTCACTGCCGCGCGGCCGCCGCGCGCAAAGCCGGCCCCGCGACGGCGTGCGGCGCAACCC
TCCGACGCTTCGCCGGCCGCGCAGGTGGATCTACGCACGCTCGGCTACAGTCAGCAGCAGCAAGAGAAGAT
CAAACCGAAGGTGCGTTCGACAGTGGCGCAGCACCACGAGGCACTGGTGGGCCATGGTTTTACACACGCGC
ACATCGTTGCGCTCAGCCAACACCCGGCAGCGTTAGGGACCGTCGCTGTCAAGTATCAGCACATAATCACGG
CGTTGCCAGAGGCGACACACGAAGACATCGTTGGCGTCGGCAAACAGTGGTCCGGCGCACGCGCCCTGGA
GGCCTTGCTCACGAAGGCGGGGgAGTTGAGAGGTCCGCCGTTACAGTTGGACACAGGCCAACTTCTCAAGA
TTGCAAAACGTGGCGGCGTGACCGCAGTGGAGGCAGTGCATGCATCGCGCAATGCACTGACGGGTGCCCCc
cTGAACCTGACCCCGGACCAAGTGGTGGCCATCGCCAGCAATATTGGCGGCAACCAGGCGCTGGAGACGGT
GCAGCGGCTGTTGCCGGTGCTGTGCCAGGACCATGGCCTGACCCCGGACCAAGTGGTGGCCATCGCCAaCA
ATAACGGCGGCAAGCAGGCGCTGGAGACGGTGCAGCGGCTGTTGCCGGTGCTGTGCCAGGCCCATGGCCTG
ACCCCGGACCAAGTGGTGGCCATCGCCAGCAATGGCGGCAAGCAGGCGCTGGAGACGGTGCAGCGGCTGT
TGCCGGTGCTGTGCCAGGCCCATGGCCTGACCCCGGACCAGGTCGTGGCCATCGCCAGCAATGGCGGCGGC
AAGCAGGCGCTGGAGACGGTGCAGCGGCTGTTGCCGGTGCTGTGCCAGGCCCATGGCCTGACCCCGGCCCA
GGTGGTGGCCATCGCCAGCAATAGTGGCGGCAAGCAGGCGCTGGAGACGGTGCAGCGGCTGTTGCCGGTG
CTGTGCCAGGACCATGGCCTGACCCCGGCCCAAGTGGTGGCCATCGCCAACAATAACGGCGGCAAGCAGGC
GCTGGAGACGGTGCAGCGGCTGTTTCCGGTGCTGTGCCAGGACCATGGCCTGACCCCGGACCAGGTGGTGA
CCATCGCCAACAATAACGGCGGCAAGCAGGCGCTGGAGACGGTGCAGCGGCTGTTGCCGGTGCTGTGCCAG
GCCCATGGCTTGATCCCGGACCAGGTGGTGGCCATCGCCAACAATAACGGCGGCAAGCAGGCGCTGGAGAC
GGTGCAGCGGCTGTTGCCGGTGCTGTGCCAGGCCCATGGCCTGACCCCGGCCCAAGTGGTGGCCATCGCCA
GCAATATTGGCGGCAAGCAGGCGCTGGAGACGGTGCAGCGGCTGTTGCCGGTGCTGTGCCAGGCCCATGGC
CTGACCCCGGCCCAAGTGGTGGCCATCGCCAACAATAACGGCGGCAAGCAGGCGCTGGAGACGGTGCAGC
GGCTGTTGCCGGTGCTGTGCCAGGACCATGGCCTGACCCCGGATCAAGTGGTGGCCATCGCCAGCAATATTG
GCGGCAAGCAGGCGCTGGAGACGGTGCAGCGCCTGTTGCCGGTGCTGTGCCAGGCCCATGGCCTGACCCCG
GACCAGGTCGTGGCCATCGCCAGCAATGGCGGCGGCAAGCAGGCGCTGGAGACGGTGCAGCGGCTGTTGC
CGGTGCTGTGCCAGGACCATGGCCTGACCCcGGACCAGGTCGTGGCCATCGCCGGCCACGATGGCGGCAAG
CAGGCGCTGGAGACGGTGCAGCGGCTGTTGCCGGTGCTGTGCCAGGACCATGGCCTGACCCCGGACCAGGT
CGTGGCCATCGCCAGCCACGATGGCGGCAAGCAGGCGCTGGAGACGGTGCAGCGGCTGTTGCCGGTGCTGT
GCCAGGACCATGGCCTGACCCTGGACCAGGTGGTGGCCATCGCCAGCAATATTGGCGGCAAGCAGGCGCTG
GAGACGGTGCAGCGGCTGTTGCCGGTGCTGTGCCAGGACCATGGCCTGACCCCGGACCAGGTCGTGGCCAT
CGCCAGCAATGGCGGCGGCAAGCAGGCGCTGGAGAGCATTGTTGCCCAGTTATCTCGCCCTGATCCGGCGTT
GGCCGCGTTGACCAACGACCACCTCGTCGCCTTGGCCTGCCTCGGCGGACGTCCTGCCCTGGATGCAGTGA
AAAaGGGATTGCCGCACGCGCCGGAATTGATCAGAAGAATCAATCGCCGTATTCCCGAACGCACGTCCCATC
GCGTTGCCGACTACGCGCAAGTGGTTCGCGTGCTGGAGTTTTtCCAGTGCCACTCCCACCCAGCGTACGCAT
TTGATGAGGCCATGACGCAGTTCGGGATGAGCAGGAACGGGTTGGTACAGCTCTTTCGCAGAGTGGGCGTC

ACCGAACTCGAAGCCCGCGGTGGAACGCTCCCCccAGCCTCGCAGCGTTGGGACCGTATCCTCCAGGCATCA
GGGATGAAAaGGGCCAAACCGTCCCCTACTTCAGCTCAAACACCGGATCAGGCGTCTTTGCATGCATTCGCC
GATTCGCTGGAGCGTGACCTTGATGCGCCTAGCCCAATGCACGAGGGAGATCAGACaGGGGCAAGCAGCCG
TAAACGGTCCCGATCGGATCGTGCTGTCACCGGCCCCTCCGCACAGCAATCTTTCGAGGTGCGCGTTCCCGA
ACAGCGCGATGCGCTGCATTTGCCCCTCAGCTGGAGGGTAAAACGCCCGCGTACCAGGATCGGGGgCGGCC
TCCCGGATCCTGGTACGCCCATCGCTGCCGACCTGGCAGCGTCCAGCACCGTGATGTGGGAACAAGATGCG
GCCCCcTTCGCAGGGGCAGCGGATGATTTCCcGGCATTCAACGAAGAGGAGCTCGCATGGTTGATGGAGCTA
TTGCCTCAGTCAGGCTCAGTCGGAGGGACGATCTGA

>*tal1b* (3312 bp)
ATGGATCCCATTCGTTCGCGCACGCCAAGTCCTGCCCGCGAGCTTCTGCCCGGACCCCAACCGGATAGGGTT
CAGCCGACTGCAGATCGGGGGGGGGCTCCGCCTGCTGGCGGCCCCCTGGATGGCTTGCCCGCTCGGCGGAC
GATGTCCCGGACCCGGCTGCCATCTCCCCCTGCGCCCTCGCCTGCGTTCTCGGCGGGCAGCTTCAACGATCT
GCTCCGTCAGTTCGATCCGTCGCTTCTTGATACATCGCTTCTTGATTCGATGCCTGCCGTCGGCACGCCGCAT
ACAGCGGCTGCCCCAGCAGAGTGGGATGAGGTGCAATCGGGTCTGCGTGCAGCCGATGACCCGCCACCCAC
CGTGCGTGTCGCTGTCACTGCCGCGCGGCCGCCGCGCGCAAAGCCGGCCCCGCGACGGCGTGCGGCGCAA
CCCTCCGACGCTTCGCCGGCCGCGCAGGTGGATCTACGCACGCTCGGCTACAGTCAGCAGCAGCAAGAGAA
GATCAAACCGAAGGTGCGTTCGACAGTGGCGCAGCACCACGAGGCACTGGTGGGCCATGGTTTTACACACG
CGCACATCGTTGCGCTCAGCCAACACCCGGCAGCGTTAGGGACCGTCGCTGTCAAGTATCAGCACATAATCA
CGGCGTTGCCAGAGGCGACACACGAAGACATCGTTGGCGTCGGCAAACAGTGGTCCGGCGCACGCGCCCT
GGAGGCCTTGCTCACGAAGGCGGGGGAGTTGAGAGGTCCGCCGTTACAGTTGGACACAGGCCAACTTCTC
AAGATTGCAAAACGTGGCGGCGTGACCGCAGTGGAGGCAGTGCATGCATCGCGCAATGCACTGACGGGTGC
CCCCCTGAACCTGACCCCGGACCAAGTGGTGGCCATCGCCAGCAATATTGGCGGCAACCAGGCGCTGGAGA
CGGTGCAGCGGCTGTTGCCGGTGCTGTGCCAGGACCATGGCCTGACCCCGGACCAAGTGGTGGCCATCGCC
AACAATAACGGCGGCAAGCAGGCGCTGGAGACGGTGCAGCGGCTGTTGCCGGTGCTGTGCCAGGCCCATG
GCCTGACCCCGGACCAAGTGGTGGCCATCGCCAGCAATGGCGGCAAGCAGGCGCTGGAGACGGTGCAGCG
GCTGTTGCCGGTGCTGTGCCAGGCCCATGGCCTGACCCCGGACCAGGTCGTGGCCATCGCCAGCAATGGCG
GCGGCAAGCAGGCGCTGGAGACGGTGCAGCGGCTGTTGCCGGTGCTGTGCCAGGCCCATGGCCTGACCCC
GGCCCAGGTGGTGGCCATCGCCAGCAATAGTGGCGGCAAGCAGGCGCTGGAGACGGTGCAGCGGCTGTTG
CCGGTGCTGTGCCAGGACCATGGCCTGACCCCGGCCCAAGTGGTGGCCATCGCCAACAATAACGGCGGCAA
GCAGGCGCTGGAGACGGTGCAGCGGCTGTTTCCGGTGCTGTGCCAGGACCATGGCCTGACCCCGGACCAGG
TGGTGACCATCGCCAACAATAACGGCGGCAAGCAGGCGCTGGAGACGGTGCAGCGGCTGTTGCCGGTGCTG
TGCCAGGCCCATGGCTTGATCCCGGACCAGGTGGTGGCCATCGCCAACAATAACGGCGGCAAGCAGGCGCT
GGAGACGGTGCAGCGGCTGTTGCCGGTGCTGTGCCAGGCCCATGGCCTGACCCCGGCCCAAGTGGTGGCCA
TCGCCAGCAATATTGGCGGCAAGCAGGCGCTGGAGACGGTGCAGCGGCTGTTGCCGGTGCTGTGCCGGGCC
CATGGCCTGACCCCGGCCCAAGTGGTGGCCATCGCCAACAATAACGGCGGCAAGCAGGCGCTGGAGACGGT
GCAGCGGCTGTTGCCGGTGCTGTGCCAGGACCATGGCCTGACCCCGGATCAAGTGGTGGCCATCGCCAGCA
ATATTGGCGGCAAGCAGGCGCTGGAGACGGTGCAGCGCCTGTTGCCGGTGCTGTGCCAGGCCCATGGCCTG
ACCCCGGACCAGGTCGTGGCCATCGCCAGCAATGGCGGCGGCAAGCAGGCGCTGGAGACGGTGCAGCGGC
TGTTGCCGGTGCTGTGCCAGGACCATGGCCTGACCCCGGACCAGGTCGTGGCCATCGCCGGCCACGATGGC

GGCAAGCAGGCGCTGGAGACGGTGCAGCGGCTGTTGCCGGTGCTGTGCCAGGACCATGGCCTGACCCCGG
ACCAGGTCGTGGCCATCGCCAGCCACGATGGCGGCAAGCAGGCGCTGGAGACGGTGCAGCGGCTGTTGCC
GGTGCTGTGCCAGGACCATGGCCTGACCCTGGACCAGGTGGTGGCCATCGCCAGCAATATTGGCGGCAAGC
AGGCGCTGGAGACGGTGCAGCGGCTGTTGCCGGTGCTGTGCCAGGACCATGGCCTGACCCCGGACCAGGTC
GTGGCCATCGCCAGCAATGGCGGCGGCAAGCAGGCGCTGGAGAGCATTGTTGCCCAGTTATCTCGCCCTGAT
CCGGCGTTGGCCGCGTTGACCAACGACCACCTCGTCGCCTTGGCCTGCCTCGGCGGACGTCCTGCCCTGGAT
GCAGTGAAAAAGGGATTGCCGCACGCGCCGGAATTGATCAGAAGAATCAATCGCCGTATTCCCGAACGCAC
GTCCCATCGCGTTGCCGACTACGCGCAAGTGGTTCGCGTGCTGGAGTTTTTCCAGTGCCACTCCCACCCAGC
GTACGCATTTGATGAGGCCATGACGCAGTTCGGGATGAGCAGGAACGGGTTGGTACAGCTCTTTCGCAGAGT
GGGCGTCACCGAACTCGAAGCCCGCGGTGGAACGCTCCCCCCAGCCTCGCAGCGTTGGGACCGTATCCTCC
AGGCATCAGGGATGAAAAGGGCCAAACCGTCCCCTACTTCAGCTCAAACACCGGATCAGGCGTCTTTGCAT
GCATTCGCCGATTCGCTGGAGCGTGACCTTGATGCGCCTAGCCCAATGCACGAGGGAGATCAGACAGGGGC
AAGCAGCCGTAAACGGTCCCGATCGGATCGTGCTGTCACCGGCCCCTCCGCACAGCAATCTTTCGAGGTGC
GCGTTCCCGAACAGCGCGATGCGCTGCATTTGCCCCTCAGCTGGAGGGTAAAACGCCCGCGTACCAGGATC
GGGGGCGGCCTCCCGGATCCTGGTACGCCCATCGCTGCCGACCTGGCAGCGTCCAGCACCGTGATGTGGGA
ACAAGATGCGGCCCCCTTCGCAGGGGCAGCGGATGATTTCCCGGCATTCAACGAAGAGGAGCTCGCATGGT
TGATGGAGCTATTGCCTCAGTCAGGCTCAGTCGGAGGGACGATCTGA

Figure S14. Nucleotide sequences of the *tal* genes tal6b from LN18 and *tal1b* from LN4.

Table S1 Strains and plasmids used in this study.

Designation	Description	Reference/Source
**Bacterial Strains**		
Escherichia coli		
DH5α	F^-endA1, *thi-1*, *recA1*, Φ80, *lacZ*, ΔM15	TransGen Biotech
BL21(DE3)	F^- *ompT*, *hsdS20*, *gal*	TransGen Biotech
X. oryzae pv. *oryzae*		
PXO99^{A}	Wild-type, Philippine race 6; harbors *avrXa27*	Hopkins et al., 1992
LN18	Wild-type, Chinese isolate, harbors *tal6b* (*avrXa27A*)	This lab
LN4	Wild-type, Chinese isolate, harbors *tal1b* (*avrXa27A*)	This lab
AH28	Wild-type, Chinese isolate	This lab
HuN37	Wild-type, Chinese isolate; harbors *tal17* (*avrXa27B*)	Lifang Ruan, Huazhong Agricultural University.
IXO221	Wild-type, Indian isolate; harbors *tal6c* (*avrXa27C*)	This lab
PH	Derived from PXO99^{A}, *tal*-free strain	Ji et al., 2016
PH/ev	PH with empty vector pHM1	This lab
PH/avrXa27	PH with pHZW-avrXa27	This lab
PH/pthXo1	PH with pHZW-pthXo1	This lab
PH/avrXa7	PH with pHZW-avrXa7	This lab
PH/tal6b	PH with pHZW-tal6b; *tal6b* is also known as *avrXa27A*	This lab
PH/tal17	PH with pHZW-tal17; *tal17* is also known as *avrXa27B*	This study
PH/tal6c	PH with pHZW-tal6c; tal6c is also known as *avrXa27C*	This study
PH/tal6b-2	PH with pHZW-tal6b-2 (mutated variant of *tal6b*)	This study
PH/tal6b-6	PH with pHZW-tal6b-6 (mutated variant of *tal6b*)	This study

**Plasmids**		
pHM1	Broad-spectrum cosmid vector, Sp^r	Hopkins et al., 1992
pZW-avrXa7	*avrXa7* in pBluescript II KS+, contains a FLAG epitope immediately downstream of the second *Sph*I site in the C-terminus of AvrXa7, Ap^r	Yang *et al.*, 2000
pHZW-avrXa7	pHM1 fused with pZW-avrXa7 at *Hin*dIII site; contains *lacZ* promoter upstream of *avrXa7*, Ap^r, Sp^r	Yang *et al.*, 2000
pZW-avrXa27	*Sph*I fragment of *avrXa27* in pZW-avrXa7; contains a FLAG epitope immediately downstream of the second *Sph*I site in the C-terminus of AvrXa27,Ap^r	Ma et al., 2018
pHZW-avrXa27	pHM1 fused with pZW-avrXa27 at *Hin*dIII, *lacZ* promoter upstream of *avrXa27*, Ap^r, Sp^r	Ma et al., 2018
pHZW-pthXo1	pHM1 fused with pZW-pthXo1 at *Hin*dIII site; *lacZ* promoter upstream of *pthXo1*, which originated from *Xoo* $PXO99^A$; Ap^r, Sp^r	Ma et al., 2018
pZW-tal6b	*Sph*I fragment of *tal6b* in pZW-avrXa7; contains a FLAG epitope immediately downstream of the second *Sph*I site in the C-terminus of *tal6b*, which originated	This lab
pHZW-tal6b	pHM1 fused with pZW-tal6b at *Hin*dIII, *lacZ* promoter upstream of *tal6b*, Ap^r, Sp^r	This lab
pZW-tal17	*Sph*I fragment of *tal17* in pZW-avrXa7; contains a FLAG epitope immediately downstream of the second *Sph*I site in the C-terminus of *tal17*, which originated	This study
pHZW-tal17	pHM1 fused with pZW-tal17 at *Hin*dIII, *lacZ* promoter upstream of *tal17*, Apr, Spr	This study
pZW-tal6c	*Sph*I fragment of *tal6c* in pZW-avrXa7; contains a FLAG epitope immediately downstream of the second *Sph*I site in the C-terminus of *tal6c*, which originated	This study
pHZW-tal6c	pHM1 fused with pZW-tal6c at *Hin*dIII, contains *lacZ* promoter upstream of *tal6c*, Ap^r, Sp^r	This study
pZW-tal6b-2	*Sph*I fragment of *tal6b-2* in pZW-avrXa7; contains a FLAG epitope immediately downstream of the second *Sph*I site in the C-terminus of *tal6b-2*, which is a	This study
pHZW-tal6b-2	pHM1 fused with pZW-tal6b-2 at HindIII, contains *lacZ* promoter upstream of *tal6b-2*, Ap^r, Sp^r	This study

pZW-tal6b-6	*Sph*I fragment of *tal6b-6* in pZW-avrXa7; contains a FLAG epitope immediately downstream of the second *Sph*I site in the C-terminus of *tal6b-6*, which is a	This study
pHZW-tal6b-6	pHM1 fused with pZW-tal6b-6 at *Hin*dIII, contains *lacZ* promoter upstream of *tal6b-6*, Ap^{r}, Sp^{r}	This study
pET30a	pBR322 origin, F1 origin, *lacI*, His-Tag, S-Tag, Km^{r}	Novagen
pET30a-avrXa27	pET30a containing *avrXa27* with N-terminal His-tag, Km^{r}	This study
pET30a-tal6b	pET30a containing *tal6b* with N-terminal His-tag, Km^{r}	This study
pET30a-avrXa27B	pET30a containing *avrXa27B* with N-terminal His-tag, Km^{r}	This study
pET30a-avrXa27C	pET30a containing *avrXa27C* with N-terminal His-tag, Km^{r}	This study
pET30a-pthXo1	pET30a containing *pthXo1* with N-terminal His-tag, Km^{r}	This lab
pHB	Binary vector, double 35S promoter, 3×N-terminal FLAG tag, Km^{r}	Mao et al., 2005
pCAMBIA1381	Binary vector containing promoterless *gusA*; used for GUS assays, Km^{r}	This lab
pHB-pthXo1	*pthXo1* cloned with N-terminal flag-tag in pHB, Km^{r}	This lab
pHB-tal6b	*Tal6b* cloned with N-terminal flag-tag in pHB, Km^{r}	This study
OsSWEET11ap∷GUS	97bp promoter region of *OsSWEET11a* cloned upstream of *gusA* in pCAMBIA1381	This study
Xa27p∷GUS	160bp promoter region of *Xa27* cloned upstream of *gusA* in pCAMBIA1381	This study

Table S2 ***Xoo*** **strains used for detection of AvrXa27/TalAO** ***tal*** **genes.**

**Strain**	**origin**	**Accession number**	**Presence (+) and absence (-) of AvrXa27/TalAO *tal* genes**
AH28	Anhui, China	NZ_CP074076	-
HuN37	Hunan, China	NZ_CP031456	+
JL25	Jilin, China	NZ_CP031457	-
JL28	Jilin, China	NZ_CP031458	-
JL33	Jilin, China	NZ_CP031459	+
LN18	Liaoning, China	CP045238	+
LN4	Liaoning, China	CP045452	+
ScYc-b	Sichuan, China	NZ_CP018087	-
XF89b	Taiwan, China	NZ_CP011532	+
XM9	Taiwan, China	NZ_CP020334	+
YC11	Jiangsu, China	NZ_CP031464	+
YN24	Yunan, China	CP018089	-
BXO1	India	CP033201	+
ICMP3125	India	NZ_CP031697	+
ITCCBB0002	India	NZ_CP046148	-
IX-280	India	NZ_CP019226	+
IXO1088	India	NZ_CP040687	-
IXO704	India	NZ_CP040604	-
JP01	Japan	NZ_CP031460	+
JW11089	South Korea	NZ_CP033193	+
K1	South Korea	NZ_CP049205	+
K2	South Korea	NZ_CP050113	+
K3	South Korea	NZ_CP050114	+
K3a	South Korea	NZ_CP050115	-
KACC 10331	South Korea	NC_006834	-
KXO85	South Korea	NZ_CP033197	+
MAFF 311018	Japan	NC_007705	+
NX0260	Nepal	NZ_CP033192	-
PXO142	Philippines	NZ_CP031698	+
PXO145	Philippines	NZ_CP013961	+
PXO211	Philippines	NZ_CP013674	+
PXO236	Philippines	NZ_CP013675	+
PXO282	Philippines	NZ_CP013676	+
PXO364	Philippines	NZ_CP033191	-
PXO404	Philippines	NZ_CP033190	+
PXO421	Philippines	NZ_CP033189	+
PXO513	Philippines	NZ_CP033188	+
PXO524	Philippines	NZ_CP013677	+

PXO563	Philippines	NZ_CP013678	+
PXO602	Philippines	NZ_CP013679	+
PXO61	Philippines	NZ_CP033187	+
PXO71	Philippines	NZ_CP013670	+
PXO79	Philippines	NZ_CP031462	-
PXO83	Philippines	NZ_CP012947	+
PXO86	Philippines	NZ_CP007166	+
PXO99[A]	Philippines	NC_010717	+
SK2-3	Thailand	NZ_CP019515	+
AXO1947	Cameroon	NZ_CP013666	-
BAI3	Burkina Faso	NZ_CP025610	-
CFBP1948	Cameroon	NZ_CP033185	-
CFBP1949	Mali	NZ_CP033184	-
CFBP1951	Mali	NZ_CP033183	-
CFBP1952	Mali	NZ_CP033182	-
CFBP7319	Burkina Faso	NZ_CP033181	-
CFBP7320	Burkina Faso	NZ_CP033186	-
CFBP7322	Burkina Faso	NZ_CP033179	-
CFBP7323	Niger	NZ_CP033178	-
CFBP7324	Niger	NZ_CP033177	-
CFBP7337	Mali	NZ_CP033175	-
CFBP7340	Niger	NZ_CP033174	-
CFBP8172	Benin	NZ_CP033173	-
CIX2374	Senegal	NZ_CP036377	-
Dak16	Tanzania	NZ_CP033172	-
MAI1	Mali	NZ_CP025609	-
MAI106	Mali	NZ_CP019089	-
MAI129	Mali	NZ_CP019090	-
MAI134	Mali	NZ_CP019091	-
MAI145	Mali	NZ_CP019092	-
MAI68	Mali	NZ_CP019085	-
MAI73	Mali	NZ_CP019086	-
MAI95	Mali	NZ_CP019087	-
MAI99	Mali	NZ_CP019088	-
T19	Tanzania	NZ_CP033171	-
Ug11	Uganda	NZ_CP033170	-
BAI23	Burkina Faso	NZ_CP036256	-
CFBP7321	Burkina Faso	NZ_CP033180	-
CFBP7325	Mali	NZ_CP033176	-
CIX298	Burkina Faso	NZ_CP036378	-
CIAT	Colombia	NZ_CP033194	+
AUST2013	Australia	NZ_CP033196	+

Table S3 Primers used in this study

Primer name	Sequence (5′-3′)	Purpose
Actin-F	GTTCCTGCTGTTTGTTCTGTTG	Amplifies *Actin* as a 137-bp fragment; used for qRT-PCR
Actin-R	ATCTCACGCATTACCCTACCTT	
OsSWEET11a-qF	AGTCGACGGGAGGGTACAG	Amplifies *OsSWEET11* as a 111-bp fragment; used for qRT-PCR
OsSWEET11a-qR	TGATGGTCAGCAGCGGC	
OsSWEET13-qF	GCCTGTCCCTGCAGCATC	Amplifies *OsSWEET13* as a 122-bp fragment; used for qRT-PCR
OsSWEET13-qR	CTCCGTCGACTTGCTCTTGTAG	
OsSWEET14-qF	TCTACGCCCCCAAGAAGGCCA	Amplifies *OsSWEET14* as a 136-bp fragment; used for qRT-PCR
OsSWEET14-qR	ACCCAACCAAGAACCACGATGC	
Xa27-qF	CTCGCCATGCTGTCGCTCGT	Amplifies *Xa27* as a 101-bp fragment; used for qRT-PCR
Xa27-qR	TAGAGAGACCAGAGACCACCAAGCA	
TALN18-F	GCCGATGACCCGCCACCCAC	Amplifies the N-terminal region of *tal* genes; used for PCR
TALN18-R	TGTCTTCGTGTGTCGCCTCT	
Xa27p-F	GTGCTATAAATAGAAGAAGAGACCCATAGAGAGC	Amplifies probe Xa27p; used for EMSA
Xa27p-R	GCTCTCTATGGGTCTCTTCTTCTATTTATAGCAC	
Xa13p-F	GTTAGATATGCATCTCCCCCTACTGTACACCACCAAAAGT	Synthesis of probe Xa13p for EMSA
Xa13p-R	ACTTTTGGTGGTGTACAGTAGGGGGAGATGCATATCTAAC	
Xa13p-1F	CTCCCCCTACTGTACACCAC	Synthesis of probe Xa13p-1 for EMSA
Xa13p-1R	GTGGTGTACAGTAGGGGGAG	
Xa13p-2F	CTACTGTACACCACCAAAAG	Synthesis of probe Xa13p-2 for EMSA
Xa13p-2R	CTTTTGGTGGTGTACAGTAG	
Xa13p-3F	GTTAGATACCCCTACTGTACACCAC	Synthesis of probe Xa13p-3 for EMSA
Xa13p-3R	GTGGTGTACAGTAGGGGTATCTAAC	
Xa13p-4F	GTTAGATACTACTGTACACCACCAAAAG	Synthesis of probe

Xa13p-4R	CTTTTGGTGGTGTACAGTAGTATCTAAC	Xa13p-4 for EMSA
Xa13p-5F	GTTAGATATGCATCTCCCCCTtagGTACACCACCAAAAGT	Synthesis of probe Xa13p-5 for EMSA
Xa13p-5R	ACTTTTGGTGGTGTACctaAGGGGGAGATGCATATCTAAC	
Xa13p-6F	GTTAGATATGCATCTCCCCCT	Synthesis of probe Xa13p-6 for EMSA
Xa13p-6R	AGGGGGAGATGCATATCTAAC	
Xa13p-7F	ACAGTAGGGGGAGATGCATA	Synthesis of probe Xa13p-7 for EMSA
Xa13p-7R	TATGCATCTCCCCCTACTGT	
Xa13p-8F	TGCATCTCCCCCTACTGTAC	Synthesis of probe Xa13p-8 for EMSA
Xa13p-8R	GTACAGTAGGGGGAGATGCA	
Xa13p-9F	GCATCTCCCCCTACTGTACACC	Synthesis of probe Xa13p-9 for EMSA
Xa13p-9R	GGTGTACAGTAGGGGGAGATGC	
Xa13p-10F	ATCTCCCCCTACTGTACAC	Synthesis of probe Xa13p-10 for EMSA
Xa13p-10R	GTGTACAGTAGGGGGAGAT	
Xa13p-11F	AGCTCCCCCTACTGTACAC	Synthesis of probe Xa13p-11 for EMSA
Xa13p-11R	GTGTACAGTAGGGGGAGCT	
Xa13p-12F	AGCTGCCCCTACTGTACAC	Synthesis of probe Xa13p-12 for EMSA
Xa13p-12R	GTGTACAGTAGGGGCAGCT	
Xa13p-13F	AGCTGCCCCTGCTGTACAC	Synthesis of probe Xa13p-13 for EMSA
Xa13p-13R	GTGTACAGCAGGGGCAGCT	
Xa13p-14F	ATCTCCCCCTACTCTACAT	Synthesis of probe Xa13p-14 for EMSA
Xa13p-14R	ATGTAGAGTAGGGGGAGAT	
Xa27p-F1	cagagtgaaaaagaaGGATCCAAACACCACCTAAGGTT	Amplifies the promoter region of *Xa27* for cloning in pCAMBIA1381
Xa27p-R1	ggtggactcctcttaaagcttTGGAGGCAGCTTCTTGGGT	

Supplemental References

Hopkins, C. M., White, F. F., Choi, S. H., Guo, A. and Leach, J. E. (1992) Identification of a family of avirulence genes from *Xanthomonas oryzae* pv. *oryzae*. Molecular Plant-Microbe Interaction 5, 451-459.

Ji, Z., Ji, C., Liu, B., Zou, L., Chen, G. and Yang, B. (2016) Interfering TAL effectors of *Xanthomonas oryzae* neutralize R-gene-mediated plant disease resistance. Nature Communications, 7, 13435.

Ma, W., Zou, L., Zhiyuan, J.I., Xiameng, X.U., Zhengyin, X.U., Yang, Y., Alfano, J.R., and Chen, G. (2018). Xanthomonas oryzae pv. oryzae TALE proteins recruit OsTFIIA γ 1 to compensate for the absence of OsTFIIA γ 5 in bacterial blight in rice. Molecular Plant Pathology 19, 2248 - 2262.

Yang, B., Zhu, W., Johnson, L. B. and White, F. F. (2000) The virulence factor AvrXa7 of *Xanthomonas oryzae* pv. *oryzae* is a type III secretion pathway-dependent nuclear-localized double-stranded DNA-binding protein. Proceedings of the National Academy of Sciences of the United States of America, 97, 9807-9812.

Mao, J., Zhang, Y., Sang, Y., Li, Q., and Yang, H. (2005). A role for *Arabidopsis* cryptochromes and COP1 in the regulation of stomatal opening. Proc. Natl. Acad. Sci. USA 102:12270-12275.
