# Supplementary material for: Tal6b/AvrXa27A, a hidden TALE targeting the susceptibility gene OsSWEET11a and the resistance gene Xa27 in rice
Source: Plant Commun. 2023 Sep 20;5(2):100721. doi: 10.1016/j.xplc.2023.100721 (PMC10873877; doi:10.1016/j.xplc.2023.100721)
Supplement: Document S2. Article plus supplemental information [file mmc2.pdf]

Plant Communications
Research article

CellPress Partner Journal

Tal6b/AvrXa27A, a hidden TALE targeting the susceptibility gene *OsSWEET11a* and the resistance gene *Xa27* in rice

Zhengyin Xu[1,3], Xiameng Xu[1,3], Ying Li[1], Linlin Liu[1], Qi Wang[1], Yijie Wang[1], Yong Wang[1], Jiali Yan[1], Guanyun Cheng[1], Lifang Zou[1], Bo Zhu[1] and Gongyou Chen[1,2,*]

[1]Shanghai Collaborative Innovation Center of Agri-Seeds, School of Agriculture and Biology, Shanghai Jiao Tong University, Shanghai 200240, China

[2]State Key Laboratory of Microbial Metabolism, Shanghai Jiao Tong University, Shanghai 200240, China

[3]These authors contributed equally to this article.

***Correspondence: Gongyou Chen (gyouchen@sjtu.edu.cn)**

https://doi.org/10.1016/j.xplc.2023.100721

ABSTRACT

Xanthomonas oryzae pv. _oryzae_ (_Xoo_) secretes transcription activator-like effectors (TALEs) to activate rice susceptibility (_S_) genes, causing bacterial blight (BB), as well as resistance (_R_) genes, leading to defense against BB. This activation follows a gene-for-gene paradigm that results in an arms race between the TALE of the pathogen and effector-binding elements (EBEs) in the promoters of host genes. In this study, we characterized a novel TALE, designated Tal6b/AvrXa27A, that activates the rice _S_ gene _OsSWEET11a_ and the rice _R_ gene _Xa27_. Tal6b/AvrXa27A is a member of the AvrXa27/TalAO class and contains 16 repeat variable diresidues (RVDs); one RVD is altered and one is deleted in Tal6b/AvrXa27A compared with AvrXa27, a known avirulence (_avr_) effector of _Xa27_. Tal6b/AvrXa27A can transcriptionally activate the expression of _Xa27_ and _OsSWEET11a_ via EBEs in their corresponding promoters, leading to effector-triggered immunity and susceptibility, respectively. The 16 RVDs in Tal6b/AvrXa27A have no obvious similarity to the 24 RVDs in the effector PthXo1, but EBE_{Tal6b} and EBE_{PthXo1} are overlapped in the _OsSWEET11a_ promoter. Tal6b/AvrXa27A is prevalent among Asian _Xoo_ isolates, but PthXo1 has only been reported in the Philippine strain $PXO99^{A}$. Genome editing of EBE_{Tal6b} in the _OsSWEET11a_ promoter further confirmed the requirement for _OsSWEET11a_ expression in Tal6b/AvrXa27A-dependent susceptibility to _Xoo_. Moreover, Tal6b/AvrXa27A resulted in higher transcription of _Xa27_ than of _OsSWEET11a,_ which led to a strong, rapid resistance response that blocked disease development. These findings suggest that Tal6b/AvrXa27A has a dual function: triggering resistance by activating _Xa27_ gene expression as an avirulence factor and inducing transcription of the _S_ gene _OsSWEET11a_, resulting in virulence. Intriguingly, Tal6b/AvrXa27A, but not AvrXa27, can bind to the promoter of _OsSWEET11a_. The underlying recognition mechanism for this binding remains unclear but appears to deviate from the currently accepted TALE code.

Key words: *Xanthomonas oryzae* pv. *oryzae*, TALE, AvrXa27, tal6b, *Xa27*, *OsSWEET11a*

Xu Z., Xu X., Li Y., Liu L., Wang Q., Wang Y., Wang Y., Yan J., Cheng G., Zou L., Zhu B., and Chen G. (2024). Tal6b/AvrXa27A, a hidden TALE targeting the susceptibility gene *OsSWEET11a* and the resistance gene *Xa27* in rice. Plant Comm. **5**, 100721.

INTRODUCTION

Effectors are proteins secreted by pathogens into or near host cells that increase pathogen virulence (Koseoglou et al., 2022). Effectors can interfere with pathogen-associated molecular pattern-triggered immunity, which is the first layer of the plant innate immune system; this interference can result in effector-triggered susceptibility (ETS) (Jones and Dangl, 2006). In some cases, effectors are intercepted by host resistance (*R*) genes that activate effector-triggered immunity (ETI) (Jones and Dangl, 2006; Zhou and Zhang, 2020). Transcription activator-like effectors

Published by the Plant Communications Shanghai Editorial Office in association with Cell Press, an imprint of Elsevier Inc., on behalf of CSPB and CEMPS, CAS.

© 2023 Shanghai Jiao Tong University.
This is an open access article under the CC BY license (http://creativecommons.org/licenses/by/4.0/).

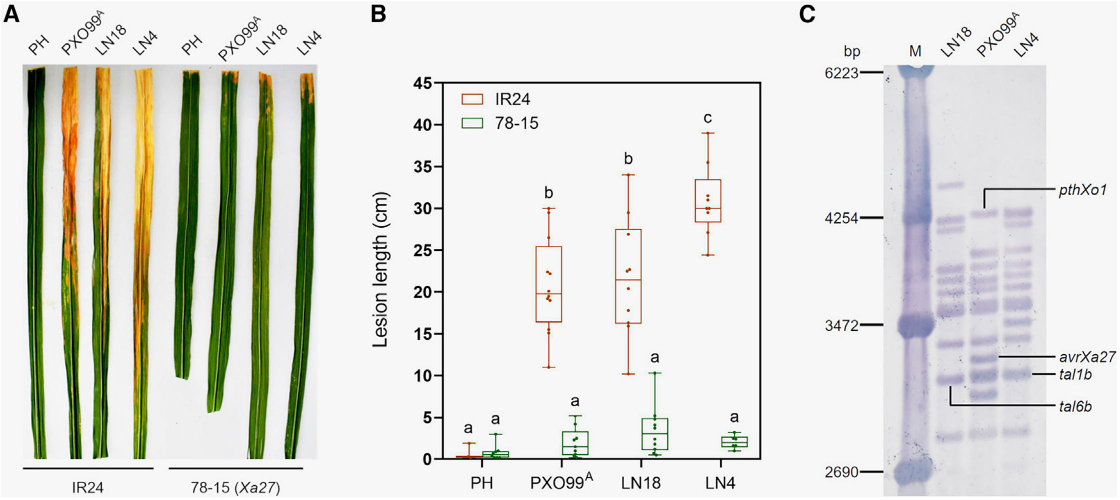

Figure 1. *Xoo* strains LN18 and LN4 do not cause disease on *Xa27*-containing rice.
(A) Disease symptoms on rice cultivars IR24 and 78-15 after inoculation with *Xoo* strains PXO99^A, PH, LN18, and LN4. The images were taken 14 days post-inoculation (dpi).
(B) Boxplots of mean disease lesion lengths (cm) on cvs. IR24 and 78-15. Lesions were measured at 14 dpi; dots denote individual observations from at least 5 inoculated leaves, and whiskers display the first and third quartiles, split by the median. Values with the same lowercase letters do not differ significantly at $P < 0.05$ according to ANOVA.
(C) Genomic digest of *tal* genes in *Xoo* strains PXO99^A, LN18, and LN4 as determined by Southern blot analysis. Genomic DNA was digested with *Bam*HI, blotted onto nylon membranes, and hybridized with the *Sph*I fragment from *avrXa27*.

(TALEs) are translocated into host plants via the type III secretion system in phytopathogenic bacteria of the genera *Xanthomonas* and *Ralstonia* (Boch and Bonas, 2010; Boch et al., 2014; Xu et al., 2017). After injection into plant cells, TALEs bind to EBEs (effector-binding elements), which are sequences in the promoters of host genes; activation of these genes can reprogram the host transcriptome (Boch et al., 2014). The specificity of EBE binding is determined by repeat-variable diresidues (RVDs) that are located at amino acid residues 12 and 13 in the central repeat regions (CRRs) of TALEs (Boch et al., 2009; Moscou and Bogdanove, 2009). Genes targeted and activated by TALEs include host susceptibility (*S*) and *R* genes (Yang and White, 2004; White and Yang, 2009; Zhang et al., 2015).

Activation of *S* genes can result in compatible host–pathogen interactions and disease symptoms (White and Yang, 2009; Hutin et al., 2015a; Koseoglou et al., 2022). The bacterial blight (BB) pathogen, *Xanthomonas oryzae* pv. *oryzae* (*Xoo*), is known to manipulate the rice *SWEET* gene family (Niño-Liu et al., 2006; Streubel et al., 2013). Three *SWEET* genes in clade III are targeted by nine major TALEs. These *SWEET* genes and their targets include *OsSWEET11a* (also known as *OsSWEET11*/*Xa13*), which is targeted by PthXo1 (Yang et al., 2006), and *OsSWEET13* (*Xa25*/*xa25*), which is induced by several PthXo2-like TALEs (PthXo2, PthXo2B/Tal7$_{PXO61}$, PthXo2C/Tal5$_{LN18}$, and Tal7$_{K74}$) (Zhou et al., 2015; Xu et al., 2019; Oliva et al., 2019). *OsSWEET14* is induced by AvrXa7, PthXo3, TalC, and TalF/Tal5, which target the *OsSWEET14* promoter at different or overlapping EBEs (Antony et al., 2010; Yu et al., 2011; Streubel et al., 2013; Tran et al., 2018). Three other *SWEET* genes, *OsSWEET12*, *OsSWEET15*, and *OsSWEET11b*, are potential *S* genes for BB that lack corresponding TALEs (Streubel et al., 2013; Wu et al., 2022). It is important to note that unidentified TALEs likely exist in *Xoo* strains isolated from rice paddies.

Approximately 47 *R* genes that confer resistance to BB have been reported in rice, and at least 17 *R* genes have been cloned and characterized (Jiang et al., 2020; Xing et al., 2021; Xu et al., 2022; Zhang et al., 2022). The functions of eight *R* genes are directly related to the process of TALE-mediated host-gene induction (Jiang et al., 2020; Zhang et al., 2022); these include four recessive *R* genes (*xa5*, *xa13*, *xa25*, and *xa41*) (Hutin et al., 2015a; 2015b) and four dominant TALE-dependent *R* genes known as executor (*E*) genes (*Xa7*, *Xa10*, *Xa23*, and *Xa27*) (Zhang et al., 2015; Ji et al., 2022). *Xa27* was originally derived from wild rice (*Oryza minuta*) and confers dominant resistance to BB (Gu et al., 2004; 2005). *Xa27* harbors an EBE in its promoter that recognizes the cognate TALE AvrXa27 (Gu et al., 2005; Römer et al., 2009). The Xa27 protein contains a signal-anchor-like sequence in its N terminus that is essential for subcellular localization and resistance to *Xoo* (Gu et al., 2005; Wu et al., 2008).

Among the TALEs identified in *Xoo*, AvrXa7 and PthXo3 are unique because they have dual functions as virulence and avirulence factors; both induce the *S* gene *SWEET14* (leading to virulence and disease) and trigger resistance in *Xa7*-containing rice (Antony et al., 2010; Römer et al., 2010; Chen et al., 2021; Luo et al., 2021). The EBEs that recognize AvrXa7 and PthXo3 in the *Xa7* promoter exhibit partial overlap, which is also true for corresponding EBEs in the *OsSWEET14* promoter (Chen et al., 2021; Luo et al., 2021). *Xa7*-mediated BB resistance is considered to be broad and durable because ETI will be triggered by *Xoo* strains harboring either AvrXa7 or PthXo3; moreover, these two TALEs also function as major virulence determinants, and their loss penalizes the pathogen with reduced virulence and fitness (Zhao and Zhang, 2021). Consequently, *Xa7* is regarded as a valuable gene for breeding BB-resistant rice. It remains unclear whether other *R* genes, such as *Xa27*, can be activated by

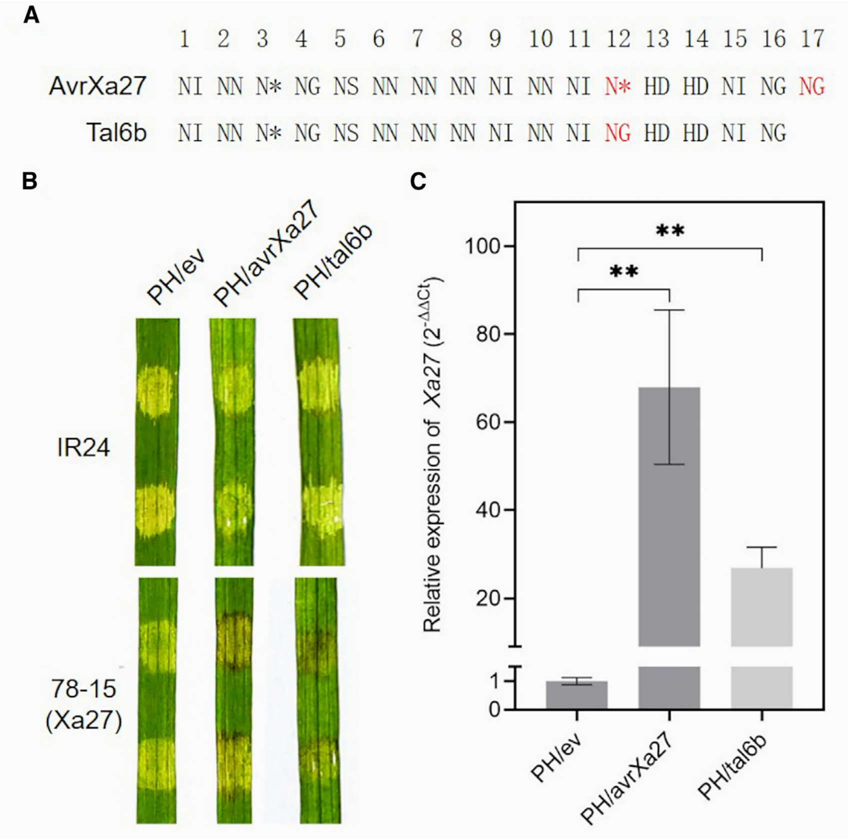

Figure 2. Tal6b, similar to AvrXa27, triggers *Xa27* resistance.

(A) Repeat variable diresidues (RVDs) in AvrXa27 and Tal6b. Single letters represent amino acids at the 12th and 13th positions of individual repeats. The asterisk indicates a predicted missing 13th residue, and RVDs in red font differ between Tal6b and AvrXa27.

(B) Reaction of IR24 and 78-15 rice leaves to *Xoo* strains PH/ev, PH/avrXa27, and PH/tal6b. Bacterial suspensions were infiltrated into rice leaves using needleless syringes, and leaves were photographed at 3 dpi.

(C) Expression of *Xa27* in 78-15 rice leaves inoculated with *Xoo* strains. The expression of *Xa27* was measured in leaves infiltrated with *Xoo* PH/ev (control), PH/avrXa27, and PH/tal6b. RNA was extracted from leaves at 24 hpi and used for qRT–PCR with *Xa27*-specific primers. *Actin* expression was used as an internal control. Columns labeled with two asterisks are significantly different at $P < 0.01$ (Student's *t*-test).

cognate avirulence factors that have dual functions and also induce *S* genes.

In the current study, we show that *Xoo* strains LN18 and LN4, which lack *avrXa27*, remain incompatible with the rice cultivar (cv.) 78-15, which harbors *Xa27*. The genomic sequences of LN18 and LN4 revealed that both encode a TALE from the AvrXa27/TalAO class (designated Tal6b/AvrXa27A) that triggers *Xa27*-mediated resistance. Four variants of TALEs in the AvrXa27/TalAO class are present in *Xoo* strains and function as avirulence factors that mediate *Xa27* resistance. Tal6b/AvrXa27A also activated *OsSWEET11a* expression and enhanced susceptibility to *Xoo* in rice. EBE prediction and gel shift assays revealed that Tal6b/AvrXa27A binds directly to the *Xa27* and *OsSWEET11a* promoters. An EBE_{Tal6b} mutation in the *OsSWEET11a* promoter rendered rice susceptible to *Xoo* strains harboring Tal6b/AvrXa27A. Our results indicate that Tal6b/AvrXa27A recognizes the *R* gene *Xa27* and the *S* gene *OsSWEET11a* and suggest that *Xa27* may mediate broad-spectrum durable resistance to BB in a manner analogous to *Xa7*.

RESULTS

Xa27 rice is resistant to *Xoo* strains LN18 and LN4, which do not contain *avrXa27*

The *Xa27* gene is a broad-spectrum *R* gene that confers resistance to 27 of 35 *Xoo* strains collected from 11 countries (Gu et al., 2004; 2005). To determine whether *Xa27* confers resistance to the hypervirulent *Xoo* strains LN18 and LN4 (Xu et al., 2019; 2020), rice cvs. 78-15 (with *Xa27*) and IR24 (without *Xa27*) were inoculated with the two strains by the tip-cutting method. Lesion lengths and symptoms on 78-15 and IR24 leaves inoculated with *Xoo* LN18 and LN4 were similar to those elicited by *Xoo* PXO99[A], which contains *avrXa27* (Figure 1A and 1B). Our results indicated that *Xoo* LN18 and LN4 are incompatible with rice cv. 78-15.

To determine whether *Xoo* LN18 and LN4 contain *avrXa27* (an *avr* gene that recognizes *Xa27*), restriction digests of *tal* genes in the two strains were hybridized with a probe that encompassed the CRR of *avrXa27* (GenBank: AY986494). The fragment containing *avrXa27* was absent from Southern blots of *Xoo* LN18 and LN4 (Figure 1C), suggesting that these two strains do not contain *avrXa27*.

The incompatibility of *Xoo* LN18 and LN4 with rice cv. 78-15 (*Xa27*) (Figure 1A and 1B) and the lack of an *avrXa27*-hybridizing band in these two strains (Figure 1C) prompted us to speculate that LN18 and LN4 may harbor a functional equivalent of *avrXa27*. Using the LN18 and LN4 genome sequences (Xu et al., 2019; 2020), we identified two orthologs of *avrXa27*, designated *tal6b* and *tal1b* (Supplemental Figure 14), in LN18 and LN4, respectively (Figure 1C). Although the PXO99[A] strain also contained a band similar to *tal6b* and *tal1b* in the Southern blot (Figure 1C), this band contained *Bam*HI fragments of two *tal* genes, *tal4* and *tal5a*, which are quite different from *avrXa27* (Salzberg et al., 2008). Sequence analysis of *tal6b* from *Xoo* LN18 indicated that the gene comprises 3312 bp with 95.8% nucleic acid identity to *avrXa27* from PXO99[A], indicating that *tal6b* encodes an AvrXa27-like TALE (designated Tal6b) of 1103 amino acid residues with 16 RVD repeats (Figure 2A). Differences occurred at the 12th RVD, where N* in AvrXa27 is NG in Tal6b; furthermore, the 17th RVD is missing in Tal6b (Figure 2A). The RVD array of Tal1b in LN4 is

TALE	RVDs[a]																	Strain
	1	2	3	4	5	6	7	8	9	10	11	12	13	14	15	16	17	
AvrXa27 (35.14%)	NI	NN	N*	NG	NS	NN	NN	NN	NI	NN	NI	N*	HD	HD	NI	NG	NG	PXO99[A], PXO86, MAFF 311018 (T7174), PXO83, PXO145, PXO211, PXO236, ICMP3125, JP01, JL33, YC11, K3, CIAT
AvrXa27A/Tal6b (48.65%)	NI	NN	N*	NG	NS	NN	NN	NN	NI	NN	NI	NG	HD	HD	NI	NG		LN18, LN4, PXO61, PXO71, PXO142, PXO282, PXO404, PXO421, PXO513, PXO524, PXO563, PXO602, KXO85, JW11089, K1, K2, XF89b, XM9
AvrXa27B (2.70%)	NI	NN	N*	NG	NS	NN	NN	NN	NN	NN	NI	NG	HD	HD	NI	NG		HuN37
AvrXa27C (13.51%)	NI	NN	N*	NG	NS	NN	NN	NN	NI	NN	NI	NG	HD	HD	NI	HG	N*	IX-280, BXO1, SK2-3, AUST2013, IXO221[b]

Table 1. Repeat variable diresidue (RVD) sequences in TALEs of the AvrXa27/TalAO class in selected *Xoo* strains.
*The RVDs in italic fontdiffer from those in AvrXa27.
[b]IXO221 is an Indian strain containing AvrXa27C; the whole genome sequence is not available.

identical to that of Tal6b (Xu et al., 2020). These results indicate that Tal6b is a functional equivalent of AvrXa27; hence, we refer to Tal6b as AvrXa27A in this study.

Tal6b/AvrXa27A, similar to AvrXa27, triggers *Xa27* resistance

To verify that Tal6b is a variant of AvrXa27, the isolated *tal6b* was subcloned as plasmid pHZW-tal6b (Supplemental Table 1); this construct was transferred into *Xoo* strain PH, a *tal*-free derivative of PXO99[A] (Ji et al., 2016), resulting in the *Xoo* strain PH/tal6b (Supplemental Table 1). The *Xoo* PH/avrXa27 strain was used as a control (Supplemental Table 1). Western blot analysis showed that Tal6b and AvrXa27 were detected as 120-kDa proteins in the corresponding *Xoo* strains (Supplemental Figure 1). *Xoo* strains PH/tal6b, PH/avrXa27, and PH/ev were infiltrated into seedling leaves of rice cv. 78-15 using needleless syringes and also inoculated by the tip-cutting method. PH/tal6b, which contains a derivative of *avrXa27*, elicited a hypersensitive response (HR) in rice cv. 78-15, as did AvrXa27 (Figure 2B). Lesion lengths on leaves of rice cv. 78-15 inoculated with PH/tal6b did not differ significantly from those on leaves inoculated with PH/avrXa27 (Supplemental Figure 2), implying that Tal6b is a functional equivalent of AvrXa27.

Gene expression assays were used to determine whether Tal6b (AvrXa27A) could activate *Xa27* expression in 78-15 rice. Expression of *Xa27* was significantly higher in 78-15 rice leaves inoculated with *Xoo* PH/avrXa27 and PH/tal6b than in leaves infiltrated with *Xoo* PH/ev (control) (Figure 2C). Collectively, these results suggest that *tal6b* functions as a derivative of *avrXa27*.

Prevalence of the AvrXa27/TalAO class in *Xoo* strains

The AvrXa27/TalAO class has been categorized previously as a "core TALE" class that is present in over 80% of Asian *Xoo* strains (Mücke et al., 2019). We investigated the distribution of *avrXa27* and homologs by performing BLAST searches of the complete genome sequences of 80 *Xoo* strains in the NCBI database. These strains originated from Asia (47 strains), Africa (31), South America (1), and Oceania (1) and represent diverse geographic areas. Our results showed that 36 *Xoo* strains contained TALEs of the AvrXa27/TalAO class, whereas 44 strains lacked AvrXa27/TalAO orthologs, including all African strains and 13 Asian strains (Supplemental Table 2). AvrXa27/TalAO class TALEs in these 36 *Xoo* strains were grouped into 4 versions based on their RVD sequences (Table 1): (1) the AvrXa27 prototype in 12 Asian and 1 South American strain; (2) the AvrXa27A type in 18 Asian strains, including LN18 and LN4, in which the 12th RVD (NG) is altered and the 17th RVD is missing; (3) the AvrXa27B type, as in strain HuN37, in which the 9th (NN) and the 12th (NG) RVDs are altered and the 17th RVD is missing; and (4) the AvrXa27C type in 2 Indian, 1 Thai, and 1 Australian strain, in which the 12th (NG), 16th (HG), and 17th (N*) RVDs are altered (Table 1). These RVD differences illustrate the diversity in the AvrXa27/TalAO class of TALEs, which is likely due to evolutionary pressure between the *avrXa27* locus and the cognate *Xa27 R* gene. Moreover, AvrXa27A appears to have a prominent role in the AvrXa27/TalAO class because it is present in 48.65% of 37 *Xoo* strains.

Xoo strains PXO99[A], HuN37, and IXO221, containing AvrXa27, Tal17/AvrXa27B, and Tal6c/AvrXa27C, respectively, were chosen for further evaluation of their interactions with *Xa27*. Inoculation assays showed that *Xoo* PXO99[A], HuN37, and IXO221 were avirulent on rice cv. 78-15 containing *Xa27*, which indicates that Tal17/AvrXa27B and Tal6c/AvrXa27C may also trigger *Xa27* resistance (Supplemental Figure 3). To evaluate this possibility, *tal17* and *tal6c* were cloned from *Xoo* HuN37 and IXO221, respectively, and introduced into *Xoo* PH. The resulting strains, *Xoo* PH/tal17 and PH/tal6c, both elicited an HR on rice 78-15 (Supplemental Figure 4), which indicates that Tal17 and Tal6c are orthologs of AvrXa27 and interact with *Xa27*. In addition, qRT–PCR assays revealed a significant upregulation of *Xa27* gene expression in rice inoculated with both PH/tal17 and PH/tal6c strains compared with the control (Supplemental Figure 5), confirming that Tal17/AvrXa27B and Tal6c/AvrXa27C can activate the expression of the *Xa27* gene. Thus, all four members of the AvrXa27/TalAO class triggered ETI via *Xa27*, which helps explain the broad-spectrum resistance of the *Xa27* executor *R* gene.

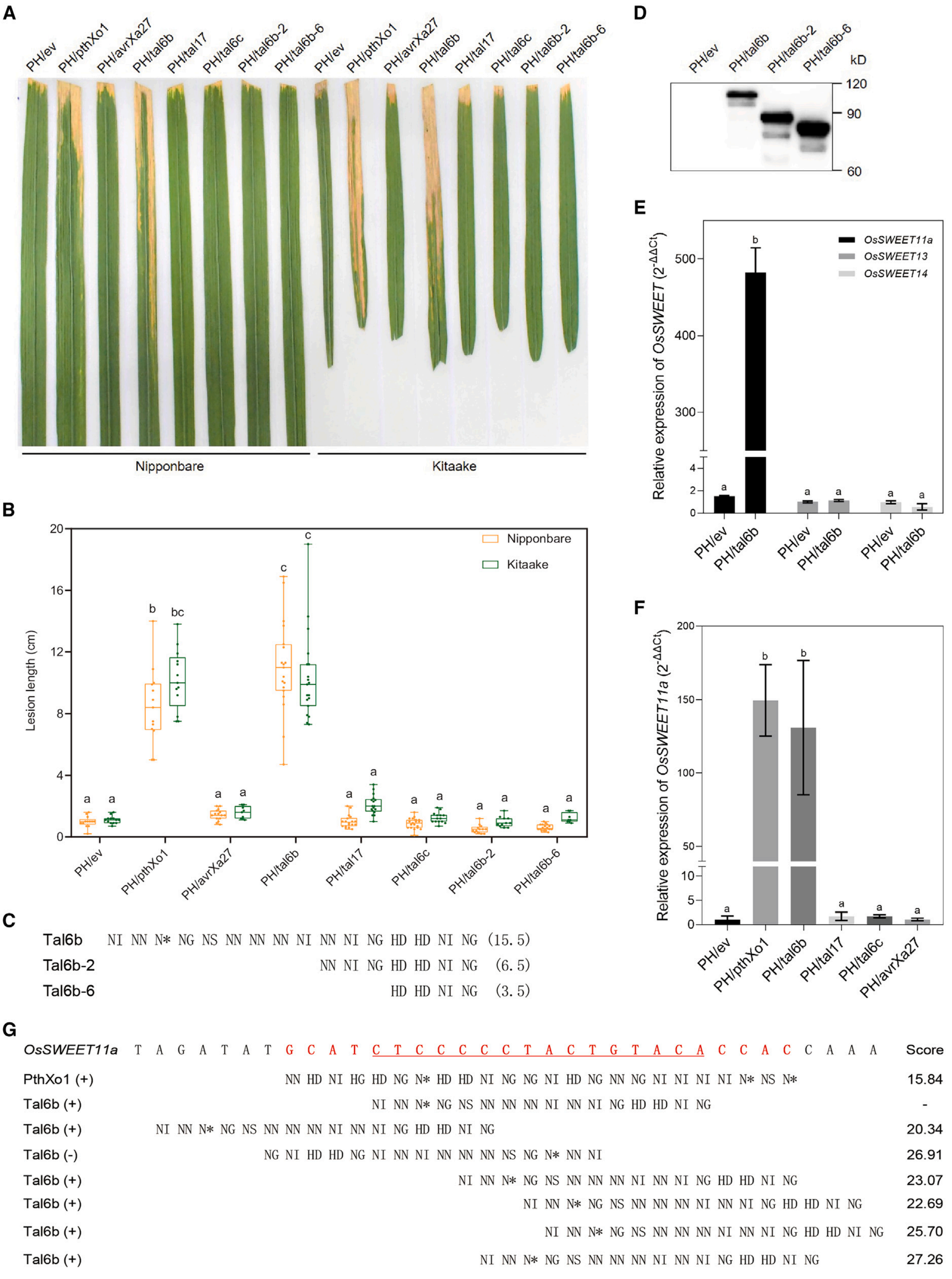

Figure 3. Tal6b/AvrXa27A is a novel major virulence factor that targets the *S* gene *OsSWEET11a*.
(A) Disease symptoms on rice cultivars Nipponbare and Kitaake after inoculation with *Xoo* strains PH/ev, PH/pthXo1, PH/avrXa27, PH/tal6b, PH/tal17, PH/tal6c, PH/tal6b-2, and PH/tal6b-6. Photos were taken at 14 dpi.

(legend continued on next page)

Tal6b/AvrXa27A is a novel virulence factor for BB

Introduction of *tal6b* into *Xoo* PH resulted in a high level of virulence on rice cv. IR24 (Supplemental Figure 2), indicating that Tal6b/AvrXa27A may also be a virulence factor. To determine whether other TALEs in the AvrXa27/TalAO class contribute to virulence, PH strains expressing the four *avrXa27* variants were inoculated onto Nipponbare and Kitaake rice using the tip-cutting method. Only *talb6* (*avrXa27A* variant) rendered PH highly virulent on Nipponbare and Kitaake at 14 days post inoculation (dpi) (Figure 3A and 3B). PH/tal6b induced BB lesions exceeding 10.0 cm in length on Nipponbare and Kitaake rice; the other three forms of *avrXa27* induced small lesions less than 3.0 cm in length (Figure 3B). Thus, our results indicate that Tal6b/AvrXa27A functions as a virulence factor in *Xoo*.

The CRRs in TALEs are known to determine their specificity (Boch et al., 2009; Moscou and Bogdanove, 2009); we therefore constructed deletion derivatives of *tal6b* that lacked various repeats to further validate the function of Tal6b as a virulence factor. Derivatives Tal6b-2 and Tal6b-6 lacked repeats 1–9 and 1–12, respectively (Figure 3C and 3D). When these derivatives were introduced into *Xoo,* strains PH/tal6b-2 and PH/tal6b-6 lost their ability to induce disease symptoms in Nipponbare and Kitaake rice (Figure 3A and 3B). Overall, our data suggested that Tal6b/AvrXa27A was a novel virulence factor targeting an unknown *S* gene in rice.

Tal6b/AvrXa27A targets the well-known *S* gene *OsSWEET11a*

We previously demonstrated that rice lines harboring triple mutations in the promoter regions of three *S* genes (*OsSWEET11a*, *OsSWEET13*, and *OsSWEET14*) exhibited broad-spectrum resistance against all tested *Xoo* strains, including LN18, which contains *tal6b* (Xu et al., 2019). To understand how Tal6b/AvrXa27A promotes disease, we sought to identify its *S* gene target. We speculated that Tal6b/AvrXa27A might target one or more of the three *OsSWEET* genes in rice. To test this hypothesis, rice Kitaake leaves were infiltrated with PH/tal6b and PH/ev, which carried *tal6b/avrXa27A* and an empty vector, respectively. The expression levels of *OsSWEET11a* were hundreds of times higher in rice plants infected with PH/tal6b than PH/ev, whereas *OsSWEET13* and *OsSWEET14* were not induced by *Xoo* PH/tal6b (Figure 3E); this suggested that Tal6b/AvrXa27 could transcriptionally activate the *S* gene *OsSWEET11a*. These findings suggested that Tal6b/AvrXa27A has a dual function; in other words, it triggers resistance by interacting with *Xa27* as an avirulence factor, and it induces transcription of the *S* gene *OsSWEET11a*, resulting in virulence.

To test whether other AvrXa27/TalAO-class TALEs also activate *OsSWEET11a*, we evaluated expression of *OsSWEET11* in Kitaake rice leaves inoculated with PH strains containing the empty vector (PH/ev), PthXo1 (PH/pthXo1), and selected AvrXa27/TalAO-class TALEs (*tal6b/avrXa27A*, *tal17/avrXa27B*, *tal6c/avrXa27C*, and *avrXa27*). Gene expression analysis showed that *pthXo1* and *tal6b* activated *OsSWEET11a* transcription, but *tal17/avrXa27B*, *tal6c/avrXa27C*, and *avrXa27* did not (Figure 3F).

Tal6b/AvrXa27A binds to the promoters of *Xa27* and *OsSWEET11a*

To analyze the Tal6b/AvrXa27A inducibility of the *OsSWEET11a* and *Xa27* promoters, we cloned the promoter regions of *OsSWEET11a* and *Xa27* upstream of a *gusA* reporter gene in pCAMBIA1381. The two promoter::GUS fusions were individually co-expressed in *Nicotiana benthamiana* with pHB-tal6b by *Agrobacterium*-mediated transformation. GUS (β-glucuronidase) activities revealed that the promoters of *OsSWEET11a* and *Xa27* were responsive to Tal6b/AvrXa27A (Supplemental Figure 6).

Activation of target genes by TALEs is dependent on the presence of an EBE in the target gene promoter (Boch et al., 2009; Moscou and Bogdanove, 2009); we therefore searched for possible EBEs recognizing Tal6b/AvrXa27A in the promoters of *Xa27* (designated $EBE_{AvrXa27A}$) and *OsSWEET11a* (designated EBE_{Tal6b}) using the programs TALE-NT and TALgetter (Figure 3G and Supplemental Figure 7; Doyle et al., 2012; Grau et al., 2013). The putative $EBE_{AvrXa27A}$ in Xa27 is a 16-bp sequence located 86–71 bp upstream of the *Xa27* ATG (Supplemental Figure 8). $EBE_{AvrXa27A}$ overlaps with the sequence of $EBE_{AvrXa27}$, and the predicted binding scores are similar (Supplemental Figure 8) (Römer et al., 2009). Six EBEs for Tal6b/AvrXa27A were predicted near EBE_{PthXo1}; these are located −256 to −223 bp upstream of the transcriptional start site of *OsSWEET11a* and have similar TALE-NT scores (Figure 3G).

We next investigated whether Tal6b/AvrXa27A binds to the predicted EBEs of *Xa27* and *OsSWEET11a* by electrophoretic

(B) Boxplots of mean disease lesion lengths (cm) on Nipponbare and Kitaake. Lesions were measured at 14 dpi; dots denote individual observations from at least 5 inoculated leaves, and whiskers display the first and third quartiles, split by the median.
(C) Individual RVDs of Tal6b and its derivatives Tal6b-2 and Tal6b-6. Single letters denote amino acids at the 12th and 13th positions of individual repeats. An asterisk indicates a predicted missing 13th residue.
(D) Western blot analysis of TALE proteins in *Xoo* PH/ev, PH/tal6b, PH/tal6b-2, and PH/tal6b-6.
(E) Expression of *OsSWEET* genes in Kitaake rice leaves inoculated with *Xoo* strains. The expression levels of *OsSWEET11a*, *OsSWEET13*, and *OsSWEET14* were measured in rice leaves infiltrated with *Xoo* PH/ev and PH/tal6b. RNA was extracted from leaves 24 hpi and used for qRT–PCR with *OsSWEET11a*-, *OsSWEET13*-, and *OsSWEET14*-specific primers. The expression level of *Actin* was used as an internal control.
(F) Expression of *OsSWEET11a* in Kitaake rice leaves inoculated with *Xoo* PH strains containing the empty vector (PH/ev) and AvrXa27/TalAO-class TALEs (*pthXo1*, *tal6b/avrXa27A*, *tal17/avrXa27B*, *tal6c/avrXa27C*, and *avrXa27*).
(G) Individual RVDs in the CCR region of PthXo1 and Tal6b/AvrXa27A recognize individual nucleotides in the predicted EBE regions of the *OsSWEET11a* promoter. Single letters denote amino acids at the 12th and 13th positions of individual repeats, and the asterisk denotes a missing amino acid at the 13th position of a particular repeat. The scores show matches between DNA sequences in the *OsSWEET11a* EBE and amino acid residues in the RVDs of Tal6b/AvrXa27A and PthXo1 (positive control); scores were predicted with the TALE-NT program. Lower scores indicate higher binding affinity between the RVDs and the target sequence. EBE_{PthXo1} is shown in red font, and EBE_{Tal6b} is underlined. (+) refers to the forward strand, and (−) refers to the reverse strand. Values with the same lowercase letters do not differ significantly at $P < 0.05$ according to ANOVA.

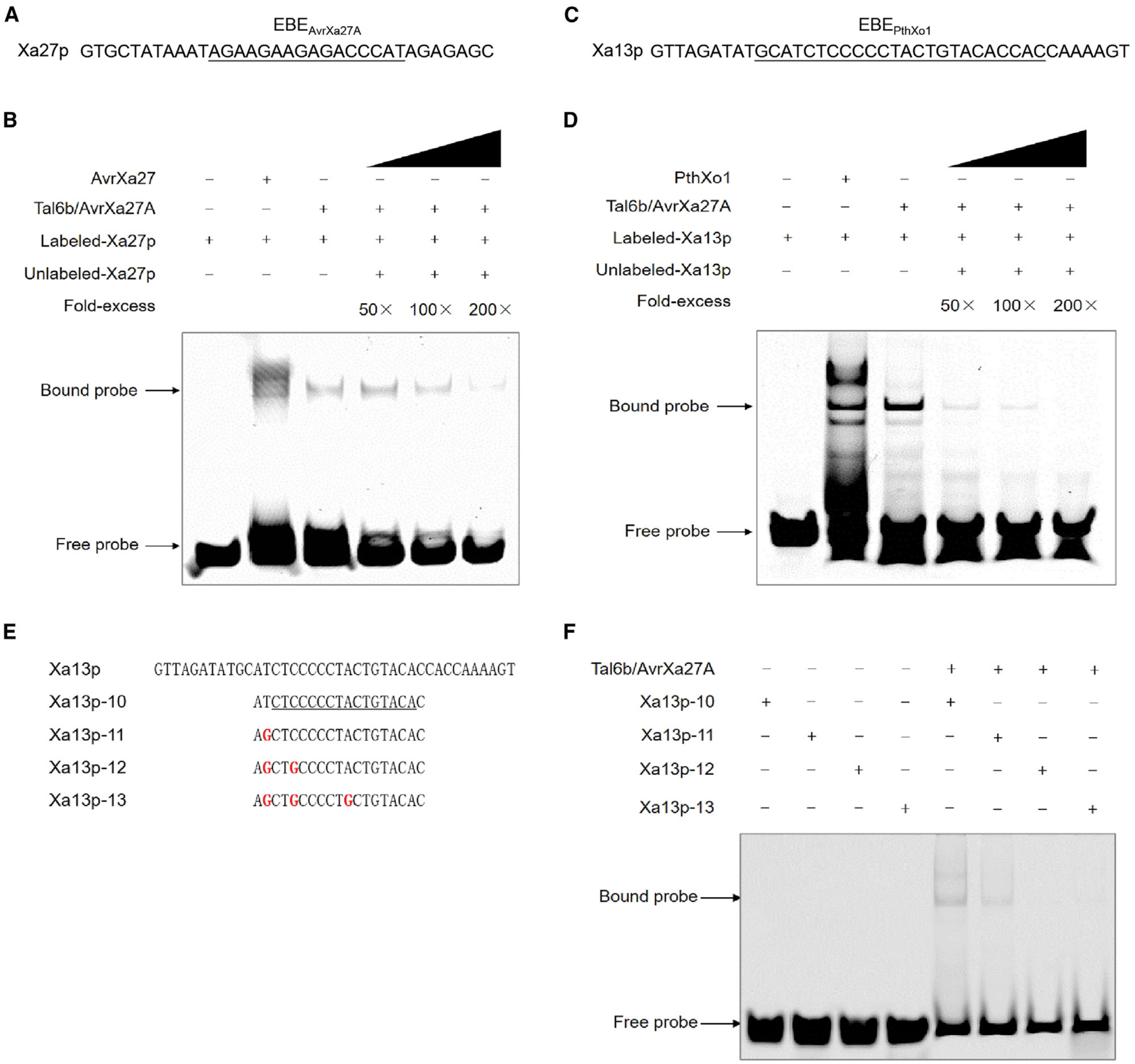

Figure 4. Tal6b/AvrXa27A binds to the EBEs of *Xa27* and *OsSWEET11a*.
(A) Nucleotide sequence of the *Xa27* promoter fragment (probe); the underscored nucleotides show the EBE recognized by Tal6b/AvrXa27A.
(B) His-tagged Tal6b/AvrXa27A fusion protein binds to the Xa27p probe derived from the *Xa27* promoter. Positions of the bound and free probe are indicated on the left.
(C) Nucleotide sequence of the *OsSWEET11a* promoter fragment (probe); EBE_{PthXo1} is underscored.
(D) His-tagged PthXo1 binds to the Xa13p probe of the *OsSWEET11a* promoter in gel-shift assays. Positions of the bound and free probe are indicated at the left.
(E) Alignment of Xa13p, Xa13p-10, Xa13p-11, Xa13p-12, and Xa13p-13 sequences. EBE_{Tal6b} is underlined. Mutations in the *OsSWEET11a* promoter sequence are indicated in red font.
(F) Binding of His-tagged Tal6b/AvrXa27A to EBE_{Tal6b} in the *OsSWEET11a* promoter.

mobility shift assays (EMSAs). The His-tagged fusion proteins His-Tal6b/AvrXa27A, His-AvrXa27, and His-PthXo1 were overproduced in *Escherichia coli* and purified. A 34-bp double-stranded DNA fragment containing $EBE_{AvrXa27A}$ (Xa27p) was used to test the interaction of Xa27 with His-Tal6b/AvrXa27A (Figure 4A). EMSA results indicated that Tal6b/AvrXa27A binds to $EBE_{AvrXa27A}$ (Figure 4B). Detection of labeled Xa27p was reduced by addition of unlabeled Xa27p (Figure 4B), demonstrating that this binding was specific. Furthermore, we also identified putative EBEs for the other two types of AvrXa27/TalAO-class TALEs in *Xa27* (Supplemental Figure 8) and verified them (Supplemental Figure 9).

Tal6b/AvrXa27A also bound to the 40-bp promoter fragment containing EBE_{PthXo1} (Xa13p) (Figure 4C and 4D). To determine the precise sequence recognized by Tal6b/AvrXa27A in the

OsSWEET11a promoter, nine additional Cy5-labeled fragments (Xa13p-1–Xa13p-9) were synthesized based on the EBE prediction scores obtained with the TALE-NT program (Figure 3G, Supplemental Figure 10A). Surprisingly, only Xa13p-5 and Xa13p-9 bound the His-Tal6b/AvrXa27A protein (Supplemental Figure 10B–10F). Because no recognition score was generated by TALE-NT (Figure 3G), a 16-bp putative EBE (−246 to −231 bp) in the *OsSWEET11a* promoter was manually aligned with the 15.5 RVDs of Tal6b/AvrXa27A. To investigate whether the 16-bp sequence is the cognate EBE_{Tal6b} of *OsSWEET11a*, 4 additional 19-bp probes (Xa13p-10–Xa13p-13) were synthesized (Figure 4E). Xa13p-10 carried the aforementioned 16-bp EBE, and Xa13p-11, Xa13p-12, and Xa13p-13 contained 1-, 2-, and 3-bp mutations, respectively, compared with Xa13p-10 (Figure 4E). Interactions of the Xa13p-10 and Xa13p-11 probes with the His-Tal6b/AvrXa27A protein were observed in gel shift assays; however, binding was not observed with Xa13p-12 or Xa13p-13 (Figure 4F). These results indicated that the EBE_{Tal6b} interacts with Tal6b/AvrXa27A at the *OsSWEET11a* promoter.

EMSAs confirmed that other members of the AvrXa27/TalAO-class TALEs, including AvrXa27, Tal17/AvrXa27B, and Tal6c/AvrXa27C, do not bind to EBE_{Tal6b} in the *OsSWEET11a* promoter (Supplemental Figure 11). Given that there are only two RVD differences between AvrXa27 and Tal6b/AvrXa27A (Figure 2A), we wondered whether these two RVDs affect the binding of AvrXa27 to EBEtal6b in the *OsSWEET11a* promoter. We therefore mutated 2 nt in the *OsSWEET11a* promoter (Supplemental Figure 12A) to match the two polymorphic RVDs in AvrXa27 according to the TALE code (Boch et al., 2009; Moscou and Bogdanove, 2009) and used the resulting probe (Xa13p-14) for EMSA. The results indicated that AvrXa27 was still unable to bind the mutated promoter fragment of *OsSWEET11a* (Supplemental Figure 12B).

OsSWEET11a promoter mutations confer Tal6b/AvrXa27A-dependent resistance to *Xoo*

We further investigated the requirement for the *OsSWEET11a* EBE_{Tal6b} in disease susceptibility in the presence of Tal6b/AvrXa27A by generating mutations in the *OsSWEET11a* EBE using CRISPR technology. Three homozygous rice lines (T_2 generation) were obtained in rice cv. Kitaake and named MS13K-14, MS13K-16, and MS13K-19. Rice line MS1K-14 carried a thymine insertion, whereas the other two lines carried 14- and 10-bp deletions, respectively (Figure 5A). The *Xoo* strains PH/ev, PH/avrXa7, PH/pthXo1, and PH/tal6b were used to evaluate EBE mutant lines for disease susceptibility. Based on lesion length, *Xoo* PH/ev was weakly virulent on Kitaake, MS13K-14, MS13K-16, and MS13K-19 rice (Figure 5B–5E). Inoculation with *Xoo* PH/pthXo1 and PH/tal6b resulted in long lesions on Kitaake but no disease lesions on MS13K-14, MS13K-16, and MS13K-19 (Figure 5B–5E), indicating that an intact EBE sequence in *OsSWEET11a* is required for Tal6b/AvrXa27A-mediated susceptibility.

DISCUSSION

In this study, we showed that Tal6b/AvrXa27A, an AvrXa27-like TALE in *Xoo*, functions as an avirulence factor to trigger *Xa27*-mediated resistance in rice. Tal6b/AvrXa27A was also shown to function as a virulence factor that targets the susceptibility gene *OsSWEET11a*. This new TALE targets *R* and *S* genes for BB by binding to the EBEs in the promoters of *Xa27* and *OsSWEET11a*, respectively.

Rice *Xa27* is an executor gene that is specifically activated by the cognate avirulence TALE AvrXa27; this TALE binds to the 16-bp $EBE_{AvrXa27}$ sequence present in the *Xa27* promoter (Gu et al., 2005; Römer et al., 2009). In this study, we analyzed the molecular diversity of *avrXa27* and identified four variants of AvrXa27/TalAO-class TALEs in 37 different *Xoo* strains (Table 1). In addition to the parental TALE, AvrXa27, *Xoo* strains may produce AvrXa27A, AvrXa27B, or AvrXa27C; these also trigger *Xa27*-mediated ETI (Supplemental Figures 4 and 5) and contain several RVDs that differ from those in AvrXa27 (Table 1). These four AvrXa27/TalAO-class TALEs were not present in the genomes of 13 Asian *Xoo* strains or accessible African strains (Supplemental Table 2). The Asian strain AH28 was originally isolated from the Anhui province of China (Xu et al., 2022; Supplemental Table 2) and lacks TALEs of the AvrXa27/TalAO class (Supplemental Table 2). *Xoo* AH28 was virulent on rice cv. 78-15, which contains *Xa27* (Supplemental Figure 3), suggesting that *Xoo* can evolve to overcome *Xa27*-mediated resistance by disposing of the corresponding *avr* gene. Similarly, rice cv. IRBB27, which encodes *Xa27*, was susceptible to five *Xoo* strains (Gu et al., 2004) that may have also lost *avrXa27*-like genes.

OsSWEET11a, a member of the clade III sugar transporters in rice, is a known *S* gene targeted by the major TALE PthXo1 (Yang et al., 2006; Streubel et al., 2013). Our data showed that Tal6b/AvrXa27A is a novel major TALE that also activates *OsSWEET11a* expression (Figure 3E and 3F). Tal6b/AvrXa27A targets an EBE in *OsSWEET11a* that overlaps with the EBE of PthXo1 and harbors a completely different RVD array compared with that in PthXo1 (Figures 3G, 4E, and 4F). Similarly, the promoters of *OsSWEET14* and *OsTFX1* are targeted by several unrelated TALEs (Sugio et al., 2007; Antony et al., 2010; Yu et al., 2011; Streubel et al., 2013; Tran et al., 2018); thus, *OsSWEET11a* represents a new case of functional convergence in which two completely unrelated TALEs from different strains have evolved to bind overlapping EBEs.

It is widely accepted that plants mount a defense response against pathogens by tricking them into inducing resistance via *E* genes, which are dominant *R* genes (Hutin et al., 2015a; Timilsina et al., 2020). Four *E* genes, *Xa27*, *Xa10*, *Xa23*, and *Xa7*, have been cloned from rice (Gu et al., 2005; Tian et al., 2014; Wang et al., 2015; Chen et al., 2021; Luo et al., 2021). *Xa7* is activated by the TALEs AvrXa7 and PthXo3, which also target the rice susceptibility gene *OsSWEET14* (Chen et al., 2021; Luo et al., 2021). The *Xa7* EBE mimics the *OsSWEET14* EBE, which protects *OsSWEET14* against exploitation by *Xoo* by triggering cell death (Luo et al., 2021). In this study, we identified another TALE, Tal6b/AvrXa27A, that targets *E* and *S* genes in rice (Figure 4). Unlike those of AvrXa7 and PthXo3, the EBE of *Xa27* for Tal6b/AvrXa27A ($EBE_{AvrXa27A}$) bears no obvious similarities to the corresponding EBE of *SWEET11a* (EBE_{Tal6b}) (Figure 4), implying that the *Xa27* EBE may not be a mimic of the *OsSWEET11a* EBE. Expression analysis showed that transcription of *Xa27* is higher than expression of *OsSWEET11a* in rice cv. 78-15 when induced

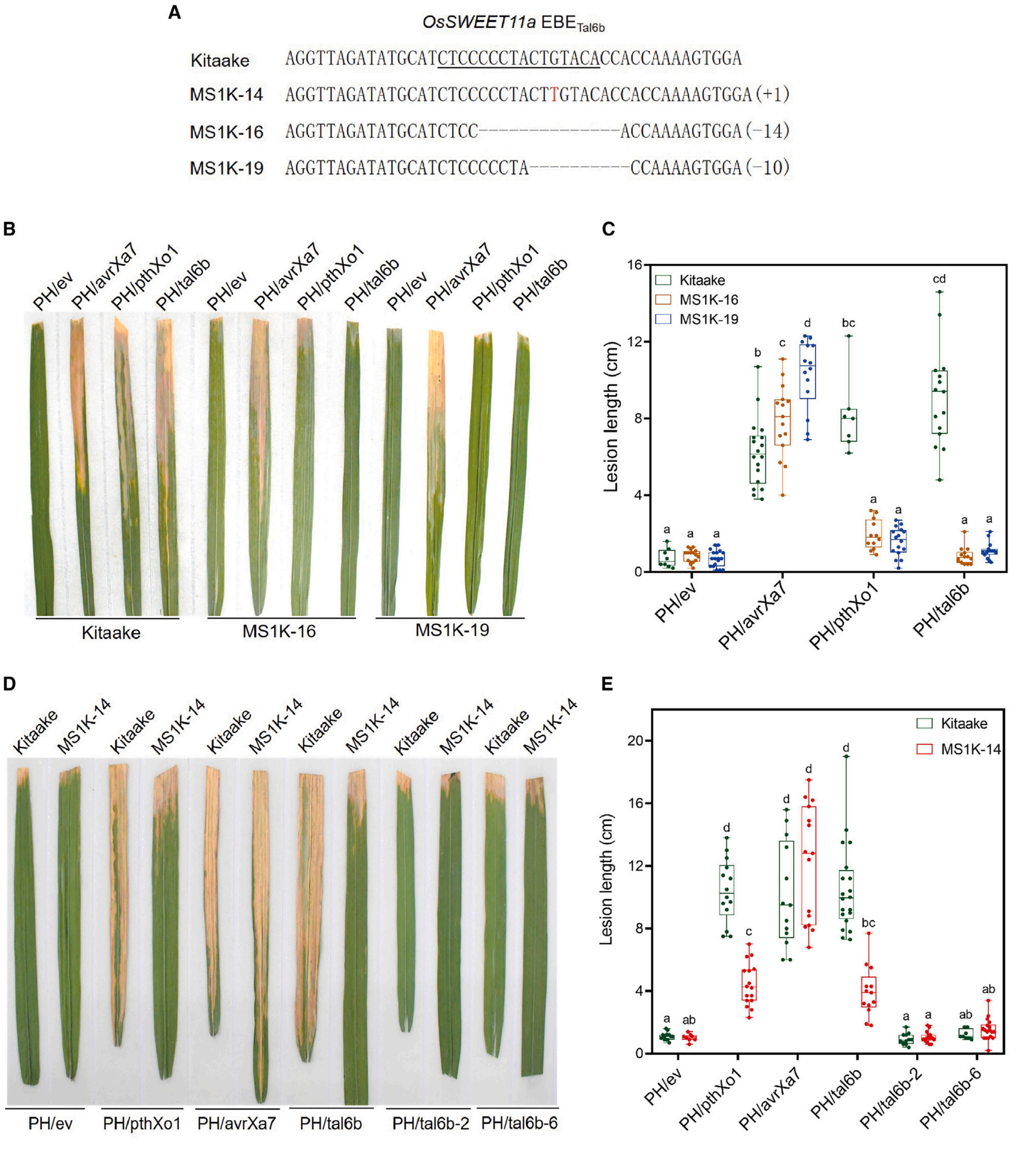

Figure 5. *OsSWEET11a* promoter mutations confer resistance to *Xoo*.

(A) DNA sequence alignment of *OsSWEET11a* EBE_{Tal6b} alleles in wild-type Kitaake rice and derived mutants.

(B) Disease symptoms on representative leaves of rice lines Kitaake, MS1K-16, and MS1K-19 after inoculation with *Xoo* strains PH/ev, PH/pthXo1, PH/avrXa7, and PH/tal6b.

(C) Boxplots of mean lesion lengths (cm) on Kitaake, MS1K-16, and MS1K-19.

(D) Disease symptoms on representative leaves of Kitaake and MS1K-14 rice after inoculation with *Xoo* strains PH/ev, PH/pthXo1, PH/avrXa7, PH/tal6b, PH/tal6b-2, and PH/tal6b-6.

(E) Boxplots of mean lesion lengths (cm) on Kitaake and MS1K-14 rice. Lesions were measured at 14 dpi. Values with identical lowercase letters do not differ significantly at $P < 0.05$ according to ANOVA.

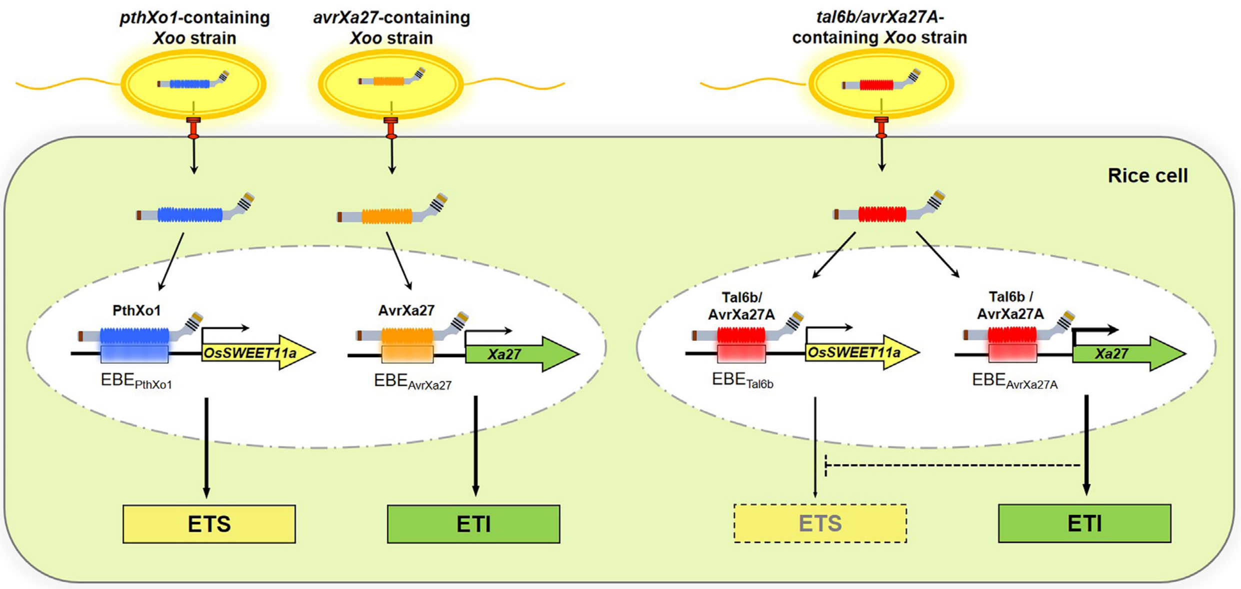

Figure 6. A working model of the role of Tal6b/AvrXa27A in the interaction between *Xoo* and rice.
pthXo1-containing *Xoo* strains employ the major TALE PthXo1 to specifically target the *S* gene *OsSWEET11a*, leading to ETS in rice. *avrXa27*-containing *Xoo* strains induce *Xa27*-mediated resistance via AvrXa27 in rice cultivars that harbor *Xa27*. Conversely, *tal6b/avrXa27A*-containing *Xoo* strains secrete Tal6b/AvrXa27A, which targets *OsSWEET11a* and *Xa27.* This results in *Xa27*-mediated ETI that suppresses *OsSWEET11a*-mediated ETS.

by Tal6b/AvrXa27A (Supplemental Figure 13). These results suggest that Tal6b/AvrXa27 has a greater capacity for inducing *Xa27* than *OsSWEET11a*, resulting in a disease-resistant phenotype. Because the co-evolution of AvrXa27/TalAO class TALE-*Xa27-OsSWEET11a* is not clear, further research is needed to confirm the hypothesis that *Xa27* evolved a promoter mimic that triggers a defense response and confounds the desired effects of the pathogen (Gu et al., 2005).

All four known types of AvrXa27/TalAO-class TALEs demonstrated binding to the respective EBEs in the *Xa27* promoter, triggering *Xa27*-mediated ETI, regardless of differences in their RVDs (Figures 2 and 5 and Supplemental Figures 4 and 9). However, among these TALEs, only Tal6b/AvrXa27A exhibited binding to EBE_{Tal6b} in the *OsSWEET11a* promoter and induced *OsSWEET11a*-mediated ETS (Figures 3 and 5 and Supplemental Figure 11). Tal6b/AvrXa27A shares only one to two RVD differences with other AvrXa27/TalAO-class TALEs (Table 1). The distinct functions displayed by Tal6b/AvrXa27A in activating both an *R* gene and an *S* gene indicate the critical role of these differences in determining specific target recognition and activation abilities. Interestingly, despite mutations of the nucleotides that correspond to the polymorphic RVDs between AvrXa27 and Tal6b/AvrXa27A, AvrXa27 still failed to bind to the mutated promoter fragment of *OsSWEET11a* (Supplemental Figure 12). This result suggests that observed differences in activity between AvrXa27 and Tal6b/AvrXa27A cannot be attributed solely to these specific nucleotide changes. Notably, the matches between the RVDs of Tal6b/AvrXa27A and EBE_{Tal6b} identified in our study do not strictly adhere to the TALE code, because only two RVD–nucleotide pairs exhibit the best match (Supplemental Figure 7). This finding implies the limitations of the current version of the TALE code. To gain a deeper understanding of these functional differences, further investigations, such as structural analyses and mutagenesis studies, are warranted to reveal the molecular basis for the unique properties of Tal6b/AvrXa27A. These studies will contribute to a comprehensive understanding of TALE-mediated host–pathogen interactions and the mechanisms that govern target recognition and activation.

Combining our data with those reported previously (Gu et al., 2005; Yang et al., 2006), we propose a working model for the roles of Tal6b/AvrXa27A in *Xoo* and rice (Figure 6). In this model, *Xoo* strains such as $PXO99^{A}$ utilize the major TALE PthXo1 to target the *S* gene *OsSWEET11a*, which leads to ETS and virulence in rice. *Xoo* strains that harbor *avrXa27* (e.g., strain PXO86) induce *Xa27*-mediated resistance via AvrXa27 in rice cultivars that harbor *Xa27*. Finally, *tal6b/avrXa27A*-containing *Xoo* strains (e.g., strain LN18) secrete the Tal6b/AvrXa27A effector, which induces ETS by targeting *OsSWEET11a* in rice lines that lack *Xa27*. However, in rice lines that contain *Xa27*, Tal6b/AvrXa27A is recognized by *Xa27* and activates a high level of *Xa27* expression. This results in *Xa27*-mediated immunity that suppresses *OsSWEET11a*-mediated susceptibility (Figure 6). However, the mechanistic basis of *Xa27*-mediated ETI, *OsSWEET11a*-mediated ETS, and their crosstalk remains unclear and warrants further research.

This study and our previous findings (Xu et al., 2019) indicate that *Xoo* LN18 encodes three major TALEs ($Tal5_{LN18}$, AvrXa7, and Tal6b/AvrXa27A), which target the *S* genes *OsSWEET13*, *OsSWEET14*, and *OsSWEET11a*, respectively. To our knowledge, this is the first example of a *Xoo* strain encoding three major TALEs that each target a known *SWEET* gene and may constitute a robust adaptation to overcome host loss-of-susceptibility recessive resistance due to *xa13*, *xa25*, and *xa41* (Yang et al., 2006; Hutin et al., 2015b; Zhou et al., 2015; Xu et al., 2019). Thus, *Xoo* LN18 may represent an emerging, highly virulent *Xoo* population that should be monitored by genome sequencing and TALome analysis. It is

important to mention that rice lines with three edited *OsSWEET* EBEs showed resistance to *Xoo* LN18 (Xu et al., 2019), demonstrating that engineering resistance by exploiting EBEs in *OsSWEET* genes may be a promising approach for dealing with this rapidly evolving pathogen.

METHODS

Plant materials, bacterial strains, and growth conditions

Rice cultivars IR24, Nipponbare, Kitaake, and 78-15 were grown in field plots and greenhouses at Shanghai Jiao Tong University (Shanghai, China). The bacterial strains used in this study are listed in Supplemental Table 1. *E. coli* strains were grown in Luria–Bertani medium at 37°C. *Xoo* strains were grown in nutrient broth (NB) or NB supplemented with 1.5% agar at 28°C. Antibiotics were used at the following concentrations when required: ampicillin (100 μg/ml), kanamycin (25 μg/ml), and spectinomycin (50 μg/ml).

Pathogen inoculation assays

Xoo strains were cultured in NB supplemented with appropriate antibiotics at 28°C for 20 h. Bacterial suspensions (optical density 600 [OD_{600}] = 0.8) were used to inoculate 2-month-old rice plants by the tip-cutting method. Disease symptoms were recorded at 14 dpi, and lesion lengths (cm) were measured. For observation of water soaking and HR, strains (OD_{600} = 0.6) were infiltrated into 2-week-old rice seedlings with needleless syringes, and symptoms were recorded 3 days after infiltration. Five leaves were inoculated with each *Xoo* strain, and experiments were repeated three times.

Southern blotting

For *tal* gene detection, *Xoo* genomic DNA was extracted, digested with *Bam*HI, separated on agarose gels, and transferred to membranes for blotting as reported previously (Xu et al., 2019). The probe was made from a digoxigenin-labeled DNA fragment that contained the repetitive region of *avrXa27*. Bacterial genomic DNA was isolated with the Axygen Bacterial Genomic DNA Miniprep Kit. Restriction endonucleases and DNA molecular weight markers were provided by TaKaRa Bio (Japan). Digoxigenin-labeled Southern blotting kits were purchased from Roche (Switzerland), and Immobilon-Ny^+ membranes were supplied by Millipore (USA).

tal gene cloning and plasmid construction

tal17 of *Xoo* HuN37 and *tal6c* of *Xoo* IXO221 were isolated as described previously (Xu et al., 2019). In brief, genomic DNA (2–3 μg) of HuN37 and IXO221 was digested with *Bam*HI (New England Biolabs, USA) and separated on 1.3% agarose gels. DNA fragments were excised, ligated into *Bam*HI-digested pBluescript II KS(−), and transferred into *E. coli* DH5α. The resulting plasmid library was screened for TALE-containing clones by *in situ* colony blot hybridization using the 3.2-kb *Sph*I fragment of *avrXa27* as a probe. Hybridizing colonies were further evaluated by PCR with the *tal*-specific primers TALN18-F and TALN18-R (Supplemental Table 3). Putative *tal*-containing clones were confirmed by restriction enzyme digestion and Sanger sequencing. *tal17* and *tal6c* were cloned into the pZW vector in frame with C-terminal FLAG tag epitopes, resulting in pZW-tal17 and pZW-tal6c (Supplemental Table 1). These plasmids were ligated into the broad-host-range vector pHM1 at the *Hin*dIII site, generating pHZW-tal17 and pHZW-tal6c (Supplemental Table 1), which were then transferred into a *tal*-free PH strain (derived from $PXO99^A$) (Ji et al., 2016) to obtain PH/tal17 and PH/tal6c (Supplemental Table 1).

To construct deletions in the CRR, pZW-tal6b was first completely digested with *Aat*II and then partially digested with *Msc*I; fragments from 250–1600 bp were inserted into *Aat*II-*Msc*I-digested pZW-tal6b. Single colonies were selected and sequenced to confirm the size of repeat regions, and this resulted in pZW-tal6b-2 and pZW-tal6b-6 (Supplemental Table 1). The plasmids were also inserted into pHM1 to generate pHZW-tal6b-2 and pHZW-tal6b-6 (Supplemental Table 1) and transferred into the PH strain to obtain PH/tal6b-2 and PH/tal6b-6 (Supplemental Table 1).

qRT–PCR

Total RNA was isolated from inoculated plants 24 h post-inoculation using RNAiso Plus reagent (TaKaRa Bio). Trace amounts of genomic DNA were removed with RNase-free DNase I (TransGen, China) prior to synthesis of cDNA. First-strand cDNA was diluted to a final volume of 20 μL, and SYBR Green-labeled PCR fragments were amplified using the *Xa27* gene-specific primers Xa27-qF and Xa27-qR (Supplemental Table 3) and the 7500 Real-Time PCR System (Applied Biosystems, USA). The rice *Actin* gene was used as an internal control (primers Actin-F and Actin-R; Supplemental Table 3). The comparative threshold ($2^{-\Delta\Delta Ct}$) method was used to calculate relative mRNA levels. qRT–PCR experiments were performed in triplicate.

Western blots

TALE proteins were detected in *Xoo* strains by immunoblotting as described previously (Xu et al., 2019). In brief, *Xoo* strains were cultured in NB to the logarithmic phase and harvested by centrifugation. Bacterial cells were washed twice, and the concentration was adjusted to OD_{600} = 2.0 with sterile distilled water. SDS loading buffer (5×) was added to the bacterial suspensions; these were then boiled in a water bath for 10 min, separated on SDS–PAGE gels, and transferred to polyvinylidene difluoride membranes for immunoblotting with anti-FLAG (TransGen) as the primary antibody. Goat anti-rabbit immunoglobulin G (TransGen) was used for detection of primary antibodies with the EasySee Western Kit supplied by TransGen.

GUS assay

GUS measurements were carried out as described previously (Haq et al., 2022). Promoter fragments of *OsSWEET11a* and *Xa27* were cloned into the binary GUS reporter construct pCAMBIA1381 using primers listed in Supplemental Table 3. The binary vector pHB was used to clone and express TALEs PthXo1 and Tal6b in *N. benthamiana*. The effector and reporter constructs (Supplemental Table 1) were transformed into *Agrobacterium* strain EHA105 by the freeze–thaw method and then co-expressed (OD_{600} = 1.0 for each strain) in 5- to 7-week-old *N. benthamiana* leaves via *Agrobacterium*-mediated transformation. Three leaf discs (1 cm diameter) were collected 48 h post inoculation, and GUS activity was measured using 4-methylumbelliferyl-β-glucuronide. Proteins were quantified using a Bradford Protein Quantification Kit (Yeasen, Shanghai).

EMSAs

avrXa27, *tal6b*, *tal17*, and *tal6c* were cloned into the pET-30a vector with a His tag to construct the plasmids pET30a-avrXa27, pET30a-tal6b/avrXa27A, pET30a-tal17/avrXa27B, and pET30a-tal6c/avrXa27C (Supplemental Table 1). After induction with isopropyl-β-D-thiogalactopyranoside (IPTG), the fusion proteins His-AvrXa27, His-Tal6b/AvrXa27A, His-AvrXa27B, and His-AvrXa27C were produced in *E. coli* BL21(DE3) cells containing pET30a-avrXa27, pET30a-tal6b/avrXa27A, pET30a-avrXa27B, and pET30a-avrXa27C, respectively. Ni-NTA HisBind Resin (Novagen, USA) was used to purify the proteins according to the manufacturer's manual. Purified His-AvrXa27, His-Tal6b/AvrXa27A, His-AvrXa27B, and His-AvrXa27C were mixed with Cy5-labeled *Xa27* or *OsSWEET11a* promoter fragments (probes synthesized by Shanghai DNA Bioscience) and loaded onto 4.5% nondenaturing polyacrylamide gels for electrophoretic separation. An Amersham Typhoon RGB Biomolecular Imager (Cytiva, Sweden) was used to scan the fluorescence patterns in the gels to detect the Cy5 fluorophore. Three independent experiments were performed with similar results.

Generation of transgenic constructs and transgenic plants

Kitaake rice was genetically modified with CRISPR–Cas9 technology as described previously (Xu et al., 2019). In brief, target sequences were selected within the promoter regions of *OsSWEET11a*, and single-guide RNAs (sgRNAs) were designed with the CRISPR MultiTargeter program (http://www.multicrispr.net/index.html) and then synthesized. BLAST searches (http://blast.ncbi.nlm.nih.gov/Blast.cgi) of the target sequences were performed against rice genome sequences to confirm target specificity. The sgRNA and Cas9 constructs were transferred into calli of rice cv. Kitaake by *Agrobacterium*-mediated transformation (Biorun, Wuhan, China). Genomic DNA was isolated from leaves of transgenic rice using the cetyltrimethyl ammonium bromide (CTAB) method and used for PCR amplification of the target regions with specific primers (Supplemental Table 3). The resulting amplicons were subjected to Sanger sequencing.

Data analysis

The newly isolated *tal* gene sequences from *Xoo* strains in this report were used as queries in BLAST searches against the NCBI database (https://www.ncbi.nlm.nih.gov/) to identify homologous sequences. *tal* gene sequences were aligned with BioEdit software (Alzohairy, 2011). The amino acid and RVD sequences of TALEs were analyzed using SnapGene software (www.snapgene.com). The EBEs recognized by AvrXa27/TalAO-class TALEs were predicted in the promoters of *Xa27* and *OsSWEET11a* using the TALgetter tool (Grau et al., 2013) and TALE-NT (Doyle et al., 2012).

SUPPLEMENTAL INFORMATION

Supplemental information is available at *Plant Communications Online*.

FUNDING

This work was supported by the National Natural Science Foundation of China (31830072 to G.Chen, 32102147 to Z.X., and 32202243 to X.X.), the China Postdoctoral Science Foundation (2020M681309 to Z.X. and 2021M702156 to X.X.), the Shanghai Postdoctoral Excellence Program (2020277 to Z.X. and 2021229 to X.X.), and the International Postdoctoral Exchange Fellowship Program (PC2021043).

AUTHOR CONTRIBUTIONS

Z.X., X.X., Y.L., L.L., Q.W., and Yijie Wang performed the experiments. Z.X. analyzed the data. Yong Wang, J.Y., and G.Cheng contributed materials. Z.X., B.Z., L.Z., and G.Chen planned and designed the research. Z.X. and G.Chen wrote an initial version of the manuscript, which was read and revised by all authors.

ACKNOWLEDGMENTS

We thank Dr. Lifang Ruan (Huazhong Agricultural University, China) for providing Xoo strain HuN37, Dr. Ramesh V. Sonti (CSIR-Centre for Cellular and Molecular Biology, India) for providing Xoo strain IXO221, Dr. Chaozu He (Hainan University, China) for providing rice cv. 78-15, and Dr. Bing Yang (University of Missouri, USA) for providing the plasmids of the CRISPR–Cas9 system. We are also grateful to Prof. Carol Bender (Oklahoma State University, USA) for her critical review of this manuscript. No conflict of interest is declared.

Received: February 5, 2023
Revised: July 12, 2023
Accepted: September 14, 2023
Published: September 20, 2023

REFERENCES

Alzohairy, A.M. (2011). Bioedit: an important software for molecular biology. GERF Bull. Biosci **2**:60–61.

Antony, G., Zhou, J., Huang, S., Li, T., Liu, B., White, F., and Yang, B. (2010). Rice *xa13* recessive resistance to bacterial blight is defeated by induction of the disease susceptibility gene Os-*11N3*. Plant Cell **22**:3864–3876.

Boch, J., Scholze, H., Schornack, S., Landgraf, A., Hahn, S., Kay, S., Lahaye, T., Nickstadt, A., and Bonas, U. (2009). Breaking the code of DNA binding specificity of TAL-type III effectors. Science **326**:1509–1512.

Boch, J., and Bonas, U. (2010). *Xanthomonas* AvrBs3 family-type III effectors: discovery and function. Annu. Rev. Phytopathol. **48**:419–436.

Boch, J., Bonas, U., and Lahaye, T. (2014). TAL effectors-pathogen strategies and plant resistance engineering. New Phytol. **204**:823–832.

Chen, X., Liu, P., Mei, L., He, X., Chen, L., Liu, H., Shen, S., Ji, Z., Zheng, X., Zhang, Y., et al. (2021). *Xa7*, a new executor R gene that confers durable and broad-spectrum resistance to bacterial blight disease in rice. Plant Comm. **2**:100143.

Doyle, E.L., Booher, N.J., Standage, D.S., et al. (2012). TAL Effector-Nucleotide Targeter (TALE-NT) 2.0: tools for TAL effector design and target prediction. Nucleic Acids Res. **40**:W117–W122.

Gu, K., Tian, D., Yang, F., et al. (2004). High-resolution genetic mapping of *Xa27(t)*, a new bacterial blight resistance gene in rice, *Oryza sativa* L. Theor. Appl. Genet. **108**:800–807.

Gu, K., Yang, B., Tian, D., Wu, L., Wang, D., Sreekala, C., Yang, F., Chu, Z., Wang, G.L., White, F.F., et al. (2005). *R* gene expression induced by a type-III effector triggers disease resistance in rice. Nature **435**:1122–1125.

Grau, J., Wolf, A., Reschke, M., Bonas, U., Posch, S., and Boch, J. (2013). Computational predictions provide insights into the biology of TAL effector target sites. PLoS Comput. Biol. **9**:e1002962.

Haq, F., Xu, X., Ma, W., Shah, S.M.A., Liu, L., Zhu, B., Zou, L., and Chen, G. (2022). A *Xanthomonas* transcription activator-like effector is trapped in nonhost plants for immunity. Plant Comm. **3**:100249.

Hutin, M., Pérez-Quintero, A.L., Lopez, C., and Szurek, B. (2015a). MorTAL Kombat: the story of defense against TAL effectors through loss-of-susceptibility. Front. Plant Sci. **6**:535.

Hutin, M., Sabot, F., Ghesquière, A., Koebnik, R., and Szurek, B. (2015b). A knowledge-based molecular screen uncovers a broad-spectrum *OsSWEET14* resistance allele to bacterial blight from wild rice. Plant J. **84**:694–703.

Ji, Z., Ji, C., Liu, B., Zou, L., Chen, G., and Yang, B. (2016). Interfering TAL effectors of *Xanthomonas oryzae* neutralize *R*-gene-mediated plant disease resistance. Nat. Commun. **7**:13435–13439.

Ji, Z., Guo, W., Chen, X., Wang, C., and Zhao, K. (2022). Plant executor genes. Int. J. Mol. Sci. **23**:1524.

Jiang, N., Yan, J., Liang, Y., Shi, Y., He, Z., Wu, Y., Zeng, Q., Liu, X., and Peng, J. (2020). Resistance genes and their interactions with bacterial blight/leaf streak pathogens (*Xanthomonas oryzae*) in rice (*Oryza sativa* L.)-an updated review. Rice **13**:3–12.

Jones, J.D.G., and Dangl, J.L. (2006). The plant immune system. Nature **444**:323–329.

Koseoglou, E., van der Wolf, J.M., Visser, R.G.F., and Bai, Y. (2022). Susceptibility reversed: modified plant susceptibility genes for resistance to bacteria. Trends Plant Sci. **27**:69–79.

Luo, D., Huguet-Tapia, J.C., Raborn, R.T., White, F.F., Brendel, V.P., and Yang, B. (2021). The *Xa7* resistance gene guards the rice susceptibility gene *SWEET14* against exploitation by the bacterial blight pathogen. Plant Comm. **2**:100164.

Mücke, S., Reschke, M., Erkes, A., et al. (2019). Transcriptional reprogramming of rice cells by *Xanthomonas oryzae* TALEs. Front. Plant Sci. **10**:162.

Moscou, M.J., and Bogdanove, A.J. (2009). A simple cipher governs DNA recognition by TAL effectors. Science **326**:1501.

Niño-Liu, D.O., Ronald, P.C., and Bogdanove, A.J. (2006). *Xanthomonas oryzae* pathovars: model pathogens of a model crop. Mol. Plant Pathol. **7**:303–324.

Oliva, R., Ji, C., Atienza-Grande, G., et al. (2019). Broad-spectrum resistance to bacterial blight in rice using genome editing. Nat. Biotechnol. **37**:1344–1350.

Römer, P., Recht, S., and Lahaye, T. (2009). A single plant resistance gene promoter engineered to recognize multiple TAL effectors from disparate pathogens. Proc. Natl. Acad. Sci. USA **106**:20526–20531.

Römer, P., Recht, S., Strauß, T., et al. (2010). Promoter elements of rice susceptibility genes are bound and activated by specific TAL effectors from the bacterial blight pathogen, *Xanthomonas oryzae* pv. *oryzae*. New Phytol. **187**:1048–1057.

Salzberg, S.L., Sommer, D.D., Schatz, M.C., et al. (2008). Genome sequence and rapid evolution of the rice pathogen *Xanthomonas oryzae* pv. *oryzae* PXO99^{A}. BMC Genom. **9**:204.

Streubel, J., Pesce, C., Hutin, M., Koebnik, R., Boch, J., and Szurek, B. (2013). Five phylogenetically close rice *SWEET* genes confer TAL effector-mediated susceptibility to *Xanthomonas oryzae* pv. *oryzae*. New Phytol. **200**:808–819.

Sugio, A., Yang, B., Zhu, T., and White, F.F. (2007). Two type III effector genes of *Xanthomonas oryzae* pv. *oryzae* control the induction of the host genes *OsTFIIA*γ*1* and *OsTFX1* during bacterial blight of rice. Proc. Natl. Acad. Sci. USA **104**:10720–10725.

Tian, D., Wang, J., Zeng, X., Gu, K., Qiu, C., Yang, X., Zhou, Z., Goh, M., Luo, Y., Murata-Hori, M., et al. (2014). The rice TAL effector-dependent resistance protein XA10 triggers cell death and calcium depletion in the endoplasmic reticulum. Plant Cell **26**:497–515.

Tran, T.T., Pérez-Quintero, A.L., Wonni, I., et al. (2018). Functional analysis of African *Xanthomonas oryzae* pv. *oryzae* TALomes reveals a new susceptibility gene in bacterial leaf blight of rice. PLoS Pathog. **14**:e1007092.

Timilsina, S., Potnis, N., Newberry, E.A., et al. (2020). *Xanthomonas* diversity, virulence and plant–pathogen interactions. Nat. Rev. Microbiol. **18**:415–427.

Wang, C., Zhang, X., Fan, Y., Gao, Y., Zhu, Q., Zheng, C., Qin, T., Li, Y., Che, J., Zhang, M., et al. (2015). XA23 is an executor R protein and confers broad-spectrum disease resistance in rice. Mol. Plant **8**:290–302.

White, F.F., and Yang, B. (2009). Host and pathogen factors controlling the rice-*Xanthomonas oryzae* interaction. Plant Physiol. **150**:1677–1686.

Wu, L.B., Eom, J.S., Isoda, R., et al. (2022). *OsSWEET11b*, a potential sixth leaf blight susceptibility gene involved in sugar transport-dependent male fertility. New Phytol. **234**:975–989.

Wu, L., Goh, M.L., Sreekala, C., and Yin, Z. (2008). XA27 depends on an amino-terminal signal-anchor-like sequence to localize to the apoplast for resistance to *Xanthomonas oryzae* pv *oryzae*. Plant Physiol. **148**:1497–1509.

Xing, J., Zhang, D., Yin, F., et al. (2021). Identification and fine-mapping of a new bacterial blight resistance gene, *Xa47(t)*, in G252, an introgression line of Yuanjiang common wild rice (*Oryza rufipogon*). Plant Dis. **105**:4106–4112.

Xu, Z., Xu, X., Gong, Q., Li, Z., Li, Y., Wang, S., Yang, Y., Ma, W., Liu, L., Zhu, B., et al. (2019). Engineering broad-spectrum bacterial blight resistance by simultaneously disrupting variable TALE-binding elements of multiple susceptibility genes in rice. Mol. Plant **12**:1434–1446.

Xu, Z., Wang, S., Liu, L., et al. (2020). Genome resource of a hypervirulent strain LN4 of *Xanthomonas oryzae* pv. *oryzae* causing bacterial blight of rice. Plant Dis. **104**:2764–2767.

Xu, Z., Xu, X., Wang, Y., et al. (2022). A varied AvrXa23-like TALE enables the bacterial blight pathogen to avoid being trapped by *Xa23* resistance gene in rice. J. Adv. Res. **42**:263–272.

Xu, Z., Zou, L., Ma, W., Cai, L., Yang, Y., and Chen, G. (2017). Action modes of transcription activator-like effectors (TALEs) of *Xanthomonas* in plants. J. Integr. Agric. **16**:2736–2745.

Yang, B., and White, F.F. (2004). Diverse members of the AvrBs3/PthA family of type III effectors are major virulence determinants in bacterial blight disease of rice. Mol. Plant Microbe Interact **17**:1192–1200.

Yang, B., Sugio, A., and White, F.F. (2006). *Os8N3* is a host disease-susceptibility gene for bacterial blight of rice. Proc. Natl. Acad. Sci. USA **103**:10503–10508.

Yu, Y., Streubel, J., Balzergue, S., et al. (2011). Colonization of rice leaf blades by an African strain of *Xanthomonas oryzae* pv. *oryzae* depends on a new TAL effector that induces the rice Nodulin-3 *Os11N3* gene. Mol. Plant Microbe Interact **24**:1102–1113.

Zhang, J., Yin, Z., and White, F. (2015). TAL effectors and the executor *R* genes. Front. Plant Sci. **6**:641.

Zhang, B., Han, X., Yuan, W., and Zhang, H. (2022). TALEs as double-edged swords in plant–pathogen interactions: Progress, challenges, and perspectives. Plant Comm. **3**:100318.

Zhao, K., and Zhang, Q. (2021). A climate-resilient *R* gene in rice traps two pathogen effectors for broad and durable resistance to bacterial blight. Mol. Plant **14**:366–368.

Zhou, J., Peng, Z., Long, J., et al. (2015). Gene targeting by the TAL effector PthXo2 reveals cryptic resistance gene for bacterial blight of rice. Plant J. **82**:632–643.

Zhou, J.M., and Zhang, Y. (2020). Plant immunity: danger perception and signaling. Cell **181**:978–989.

Plant Communications, Volume *5*

Supplemental information

Tal6b/AvrXa27A, a hidden TALE targeting the susceptibility gene *OsSWEET11a* and the resistance gene *Xa27* in rice

Zhengyin Xu, Xiameng Xu, Ying Li, Linlin Liu, Qi Wang, Yijie Wang, Yong Wang, Jiali Yan, Guanyun Cheng, Lifang Zou, Bo Zhu, and Gongyou Chen

Supplemental Information

Tal6b/AvrXa27A, a Hidden TALE Targeting both the Susceptibility Gene *OsSWEET11a* and the Resistance Gene *Xa27* in Rice

Zhengyin Xu[1†], Xiameng Xu[1†], Ying Li[1], Linlin Liu[1], Qi Wang[1], Yijie Wang[1], Yong Wang[1], Jiali Yan[1], Guanyun Cheng[1], Lifang Zou[1], Bo Zhu[1], and Gongyou Chen[1,2*]

[1]Shanghai Collaborative Innovation Center of Agri-Seeds, School of Agriculture and Biology, Shanghai Jiao Tong University, Shanghai, 200240, China

[2]State Key Laboratory of Microbial Metabolism, Shanghai Jiao Tong University, Shanghai, 200240, China

[*] Correspondence: Gongyou Chen (gyouchen@sjtu.edu.cn).

[†] These authors contributed equally to this work.

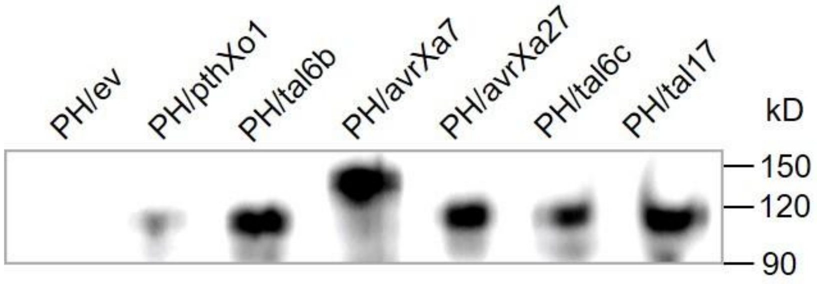

Figure S1 Western blot analysis of TALE proteins in *Xoo* PH derivatives, PH/ev, PH/pthXo1, PH/tal6b, PH/avrXa7, PH/avrXa27, PH/tal6c and PH/tal17. TALE proteins were detected using anti-FLAG as the primary antibody.

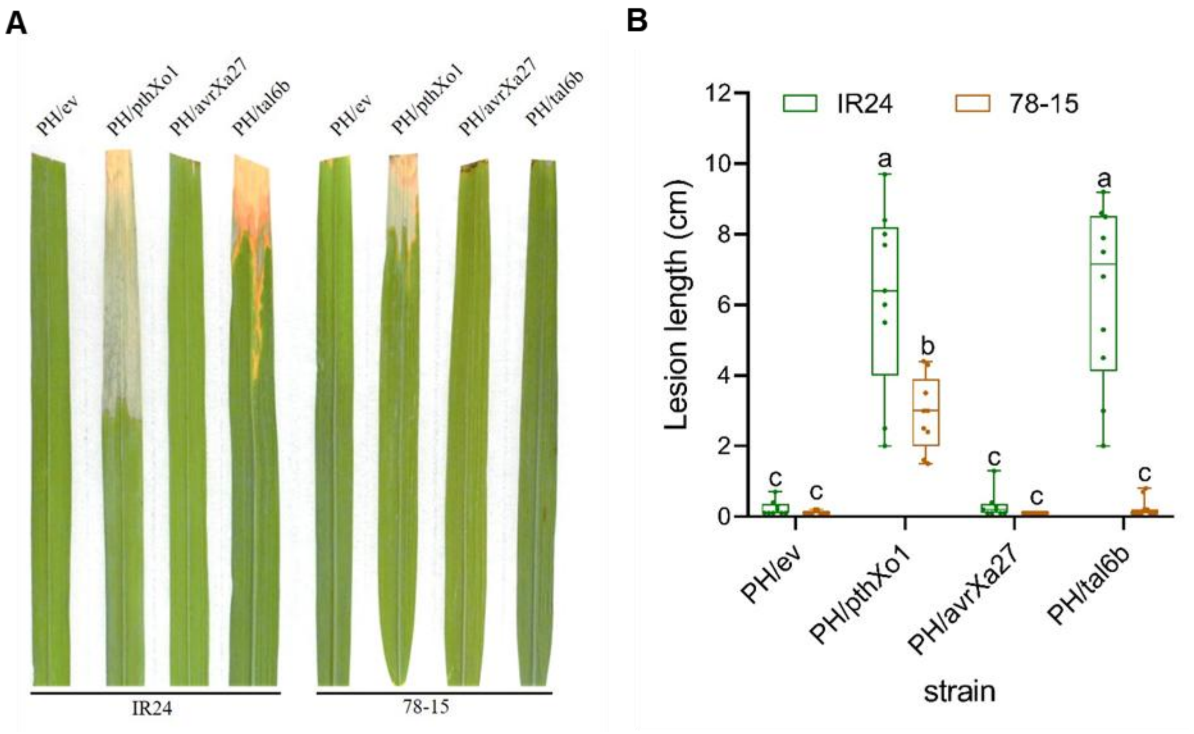

Figure S2 Disease symptoms on rice cultivars IR24 and 78-15. (A) Symptoms on rice leaves inoculated with *Xoo* PH derivatives containing the empty vector (ev), *pthXo1*, *avrXa27*, and *tal6b*. The images were taken at 14 days post-inoculation (dpi). (B) Box plots of mean disease lesion lengths (cm) on cvs. IR24 and 78-15. Lesions were measured 14 dpi; dots denote individual observations from at least five inoculated leaves, and whiskers display the first and third quartiles, split by the median. Values with the same lowercase letters do not differ significantly at $P < 0.05$ based on ANOVA.

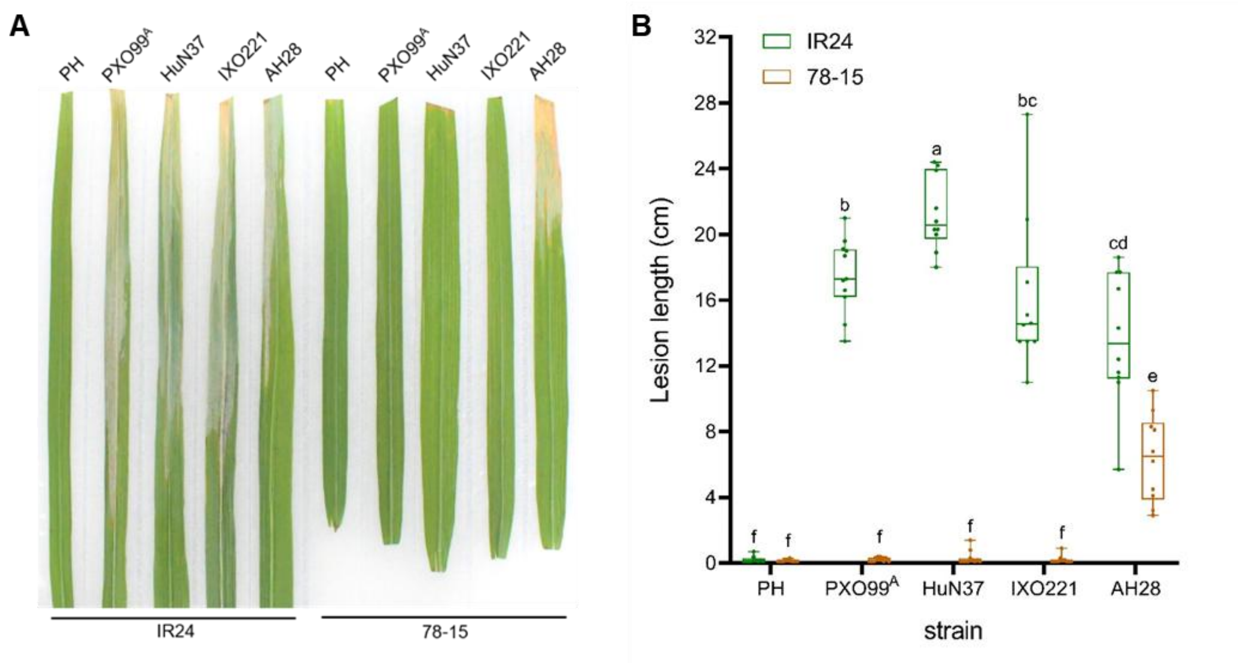

Figure S3 Symptoms on rice cultivars IR24 and 78-15 after inoculation with *Xoo* strains containing AvrXa27 orthologues. (A) Symptoms on rice leaves inoculated with *Xoo* PH (*tal*-free strain), PXO99^{A} (encodes *avrXa27*), HuN37 (*avrXa27B*), IXO221(*avrXa27C*), and AH28. (B) Box plots of mean disease lesion lengths (cm) on cvs. IR24 and 78-15. Values with the same lowercase letters do not differ significantly at $P < 0.05$ based on ANOVA.

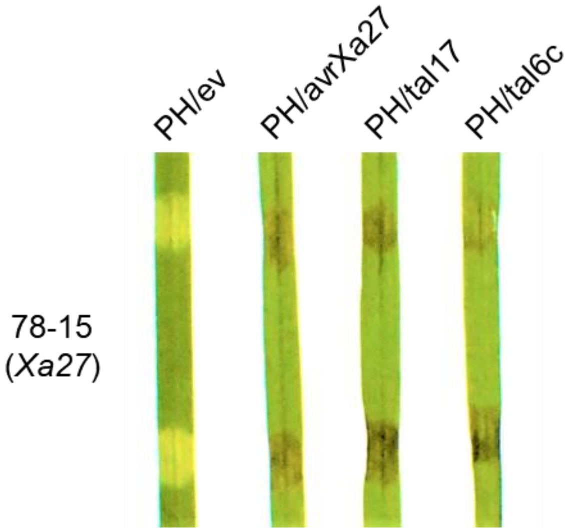

Figure S4 Rice leaves of cv. 78-15 (*Xa27*) inoculated with *Xoo* PH derivatives, PH/ev, PH/avrXa27, PH/tal17, and PH/tal6c.

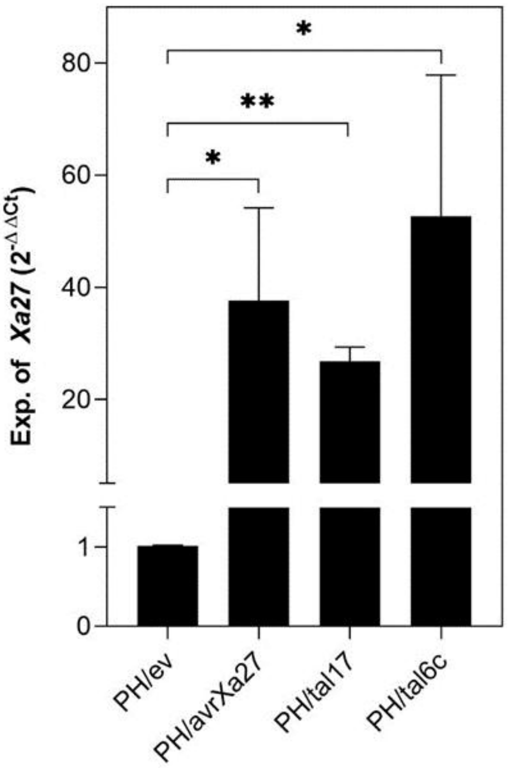

Figure S5 Expression of *Xa27* in 78-15 rice leaves inoculated *Xoo* PH derivatives, PH/ev, PH/avrXa27, PH/tal17, and PH/tal6c. Asterisks represent significant difference determined by Student's t-test (*P < 0.05, **P < 0.01).

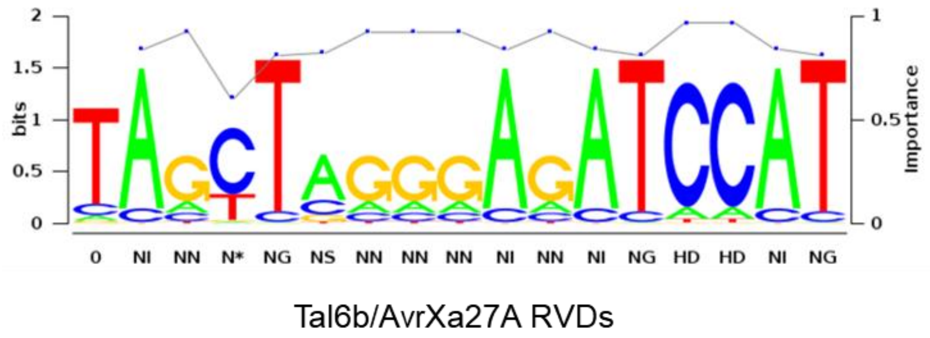

Figure S6 The predicted theoretical EBE of Tal6b/AvrXa27A RVDs. The Tal6b/AvrXa27A RVDs and their associated nucleotides are shown. The logo was produced using TALgetter (Galaxy v.1.1 http://galaxy.informatik.uni-halle.de/).

	0	1	2	3	4	5	6	7	8	9	10	11	12	13	14	15	16	17	Score
Xa27	T	A	G	A	A	G	A	A	G	A	G	A	C	C	C	A	T	A	
AvrXa27		NI	NN	N*	NG	NS	NN	NN	NN	NI	NN	NI	N*	HD	HD	NI	NG	NG	15.64
AvrXa27A		NI	NN	N*	NG	NS	NN	NN	NN	NI	NN	NI	NG	HD	HD	NI	NG		14.99
AvrXa27B		NI	NN	N*	NG	NS	NN	NN	NN	NN	NN	NI	NG	HD	HD	NI	NG		15.85
AvrXa27C		NI	NN	N*	NG	NS	NN	NN	NN	NI	NN	NI	NG	HD	HD	NI	HG	N*	17.62

Figure S7 AvrXa27/TalAO class TALEs are predicted to target EBEs in *Xa27*. EBE prediction scores were generated by TALE-NT, and lower scores indicate higher binding affinity between the RVDs and the target sequence.

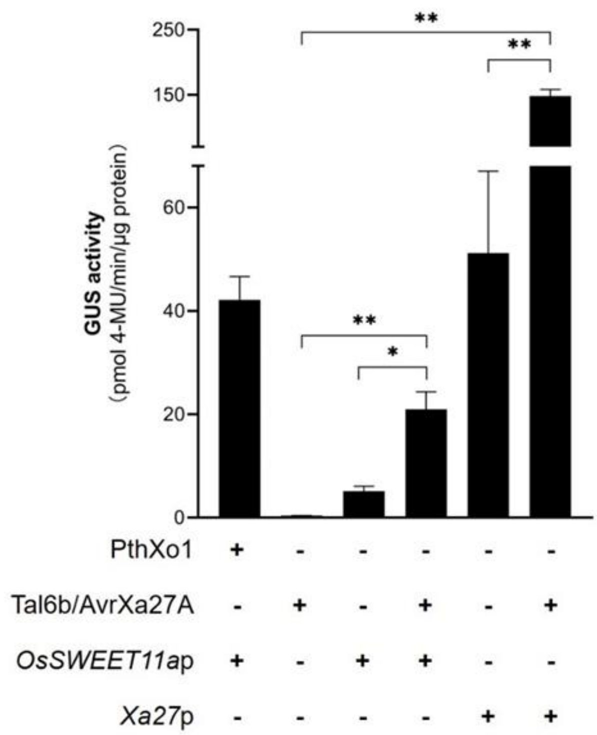

Figure S8 GUS assays show Tal6b/AvrXa27A inducibility of the *OsSWEET11a* (*OsSWEET11a*p) and *Xa27* promoter (*Xa27*p) in *Nicotiana benthamiana*. The TALE PthXo1 and the *OsSWEET11a* promoter were used as a positive control. Samples were collected at 48 hpi, and GUS activity was calculated. Error bars indicate means ±SD (n = 3). Asterisks represent significant difference determined by Student's t-test (*P < 0.05, **P < 0.01). 4-MU, 4-methyl-umbelliferone.

A

$EBE_{AvrXa27}$

Xa27p GTGCTATAAAT<u>AGAAGAAGAGACCCATA</u>GAGAGC

B

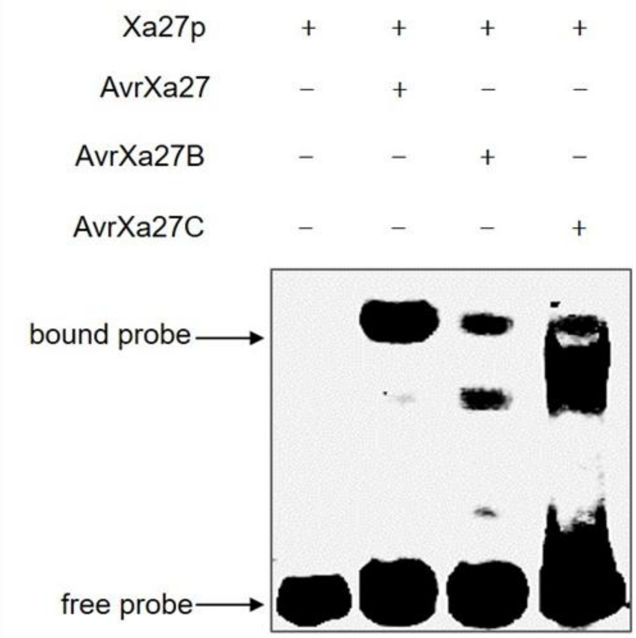

Figure S9 AvrXa27/TalAO class TALEs bind the EBEs of *Xa27*. (**A**) Nucleotide sequence of the *Xa27* promoter fragment (probe); $EBE_{AvrXa27}$ is underscored. (**B**) EMSA with His-AvrXa27, His-Tal17/AvrXa27B, and His-Tal6c/AvrXa27C fusion proteins and a Cy5-labeled *Xa27* promoter fragment (Xa27p). Positions of the bound and free probe are indicated on the left.

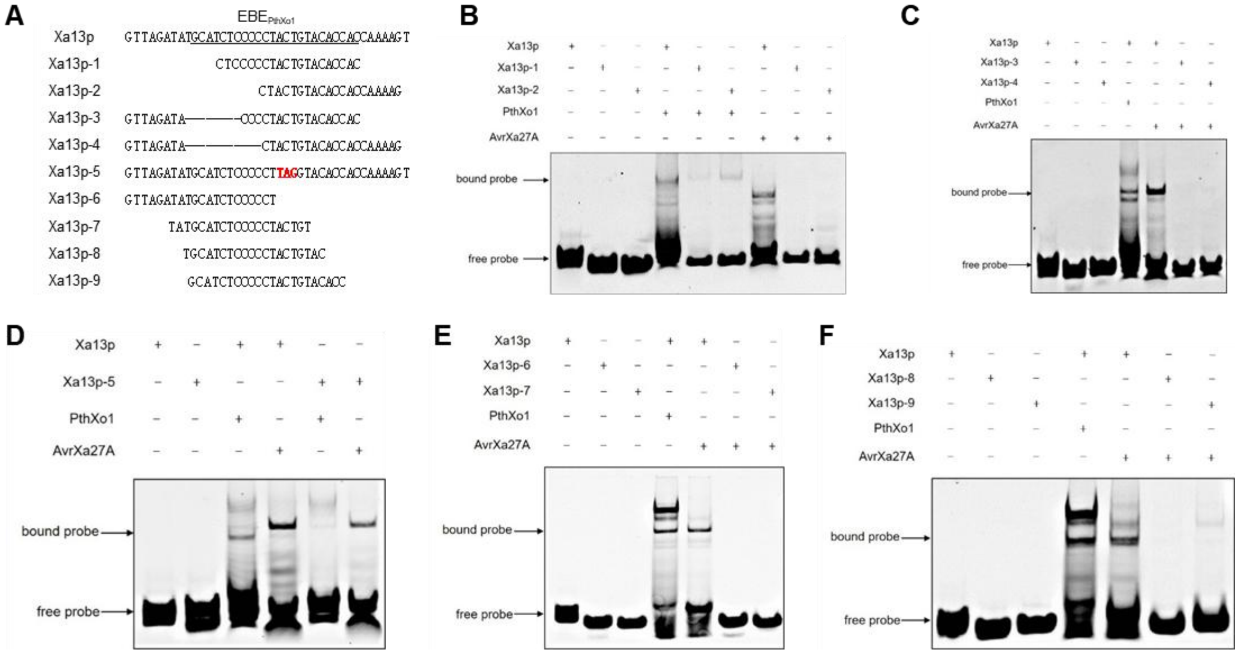

Figure S10. Tal6b/AvrXa27A does not bind to the six predicted EBEs of the *OsSWEET11a* promoter in gel shift assays. (**A**) Alignment of Xa13p, Xa13p-1, Xa13p-2, Xa13p-3, Xa13p-4, Xa13p-5, Xa13p-6, Xa13p-7, Xa13p-8, and Xa13p-9 sequences; EBE_{PthXo1} is underscored. Mutations in the *OsSWEET11a* promoter sequence are indicated in red font. (**B-F**) His-tagged PthXo1 and Tal6b/AvrXa27A interact with ten probes of the *OsSWEET11a* promoter in gel shift assays. Positions of the bound and free probe are indicated at the left.

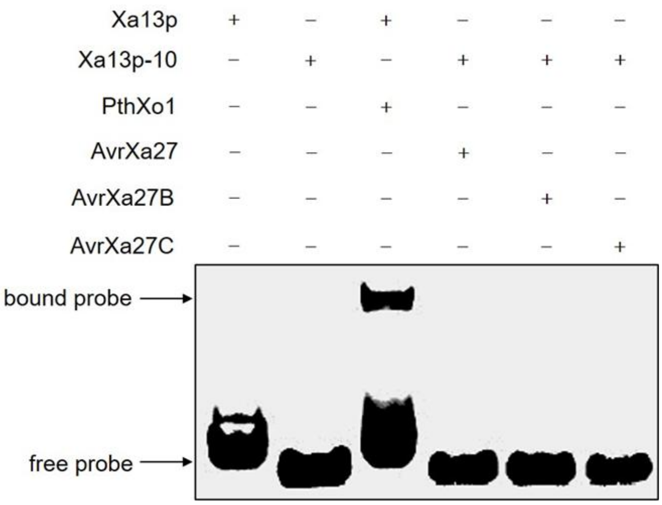

Figure S11 Other types of AvrXa27/TalAO class TALEs do not bind to the EBE_{Tal6b} of *OsSWEET11a*. EMSA with His-AvrXa27, His-Tal17/AvrXa27B, and His-Tal6c/AvrXa27C fusion proteins and a Cy5-labeled *OsSWEET11a* promoter fragment (Xa13p-10). Positions of the bound and free probe are indicated on the left.

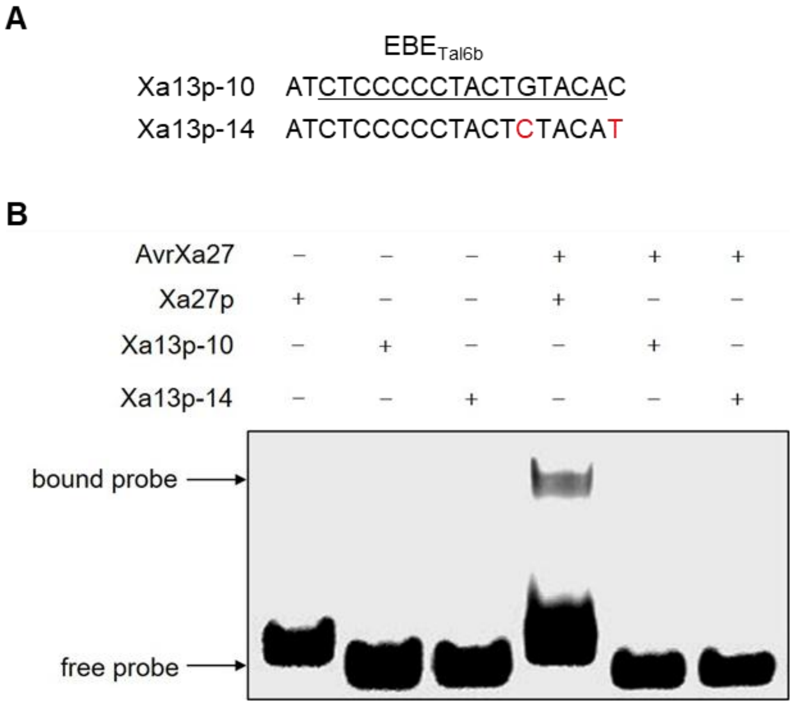

Figure S12 AvrXa27 does not bind to the mutated EBE_{Tal6b} of *OsSWEET11a*. (**A**) Alignment of Xa13p-10 and Xa13p-14 sequences. The EBE_{Tal6b} is underlined. Mutations in the *OsSWEET11a* promoter sequence are indicated in red font. (**B**) EMSA with His-AvrXa27 fusion protein and Xa13p-10 or Xa13p-14. Positions of the bound and free probe are indicated on the left.

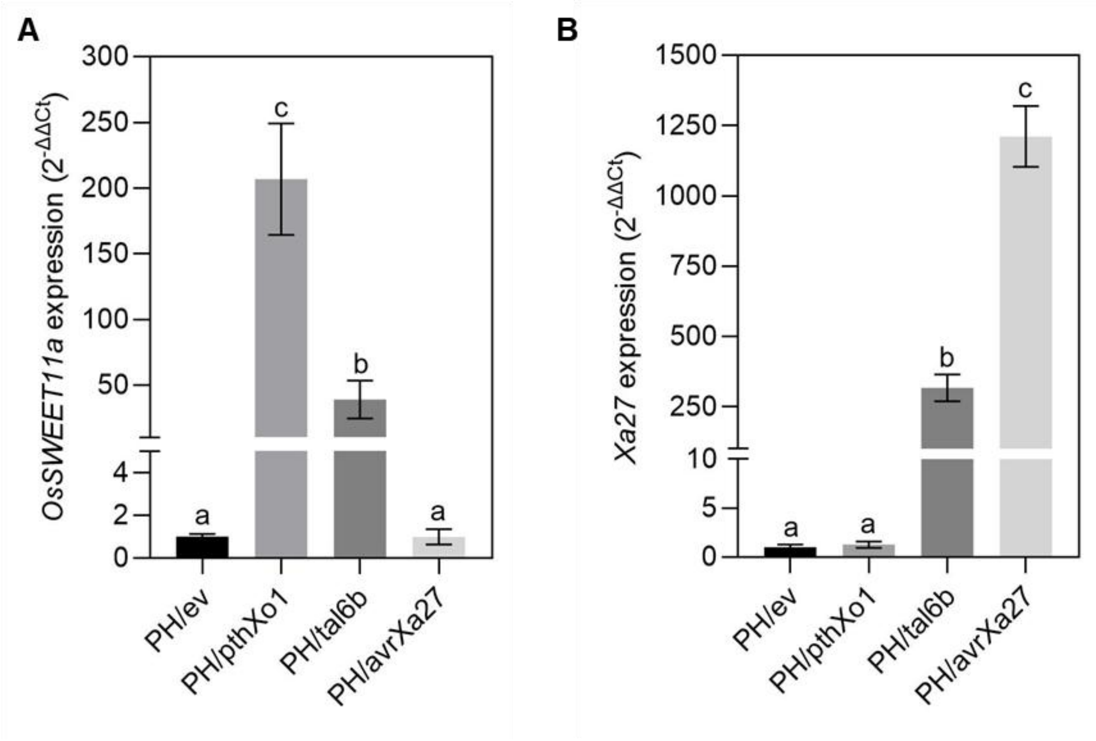

Figure S13. Expression of *OsSWEET11a* and *Xa27* in 78-15 rice leaves inoculated with *Xoo* strains. The expression of *OsSWEET11a* **(A)** and *Xa27* **(B)** was measured in rice leaves infiltrated with *Xoo* PH/ev, PH/pthXo1, PH/tal6b or PH/avrXa27. RNA was extracted from leaves 24 hpi and used for qRT-PCR with *OsSWEET11a* and *Xa27*-specific primers. The expression level of *Actin* was used as an internal control. Values with the same lowercase letters do not differ significantly at $P < 0.05$ based on ANOVA.

>*tal6b* (3312 bp)

ATGGATCCCATTCGTTCGCGCACGCCAAGTCCTGCCCGCGAGCTTCTGCCCGGACCCCAACCGGATAGGGTT
CAGCCGACTGCAGATCGGGGGggggCTCCGCCTGCTGGCGGCCCCcTGGATGGCTTGCCCGCTCGGCGGACGA
TGTCCCGGACCCGGCTGCCATCTCCCCcTGCGCCCTCGCCTGCGTTCTCGGCGGGCAGCTTCAACGATCTGCT
CCGTCAGTTCGATCCGTCGCTTCTTGATACATCGCTTCTTGATTCGATGCCTGCCGTCGGCACGCCGCATACA
GCGGCTGCCCCAGCAGAGTGGGATGAGGTGCAATCGGGTCTGCGTGCAGCCGATGACCCGCCACCCACCGT
GCGTGTCGCTGTCACTGCCGCGCGGCCGCCGCGCGCAAAGCCGGCCCCGCGACGGCGTGCGGCGCAACCC
TCCGACGCTTCGCCGGCCGCGCAGGTGGATCTACGCACGCTCGGCTACAGTCAGCAGCAGCAAGAGAAGAT
CAAACCGAAGGTGCGTTCGACAGTGGCGCAGCACCACGAGGCACTGGTGGGCCATGGTTTTACACACGCGC
ACATCGTTGCGCTCAGCCAACACCCGGCAGCGTTAGGGACCGTCGCTGTCAAGTATCAGCACATAATCACGG
CGTTGCCAGAGGCGACACACGAAGACATCGTTGGCGTCGGCAAACAGTGGTCCGGCGCACGCGCCCTGGA
GGCCTTGCTCACGAAGGCGGGGgAGTTGAGAGGTCCGCCGTTACAGTTGGACACAGGCCAACTTCTCAAGA
TTGCAAAACGTGGCGGCGTGACCGCAGTGGAGGCAGTGCATGCATCGCGCAATGCACTGACGGGTGCCCCc
cTGAACCTGACCCCGGACCAAGTGGTGGCCATCGCCAGCAATATTGGCGGCAACCAGGCGCTGGAGACGGT
GCAGCGGCTGTTGCCGGTGCTGTGCCAGGACCATGGCCTGACCCCGGACCAAGTGGTGGCCATCGCCAaCA
ATAACGGCGGCAAGCAGGCGCTGGAGACGGTGCAGCGGCTGTTGCCGGTGCTGTGCCAGGCCCATGGCCTG
ACCCCGGACCAAGTGGTGGCCATCGCCAGCAATGGCGGCAAGCAGGCGCTGGAGACGGTGCAGCGGCTGT
TGCCGGTGCTGTGCCAGGCCCATGGCCTGACCCCGGACCAGGTCGTGGCCATCGCCAGCAATGGCGGCGGC
AAGCAGGCGCTGGAGACGGTGCAGCGGCTGTTGCCGGTGCTGTGCCAGGCCCATGGCCTGACCCCGGCCCA
GGTGGTGGCCATCGCCAGCAATAGTGGCGGCAAGCAGGCGCTGGAGACGGTGCAGCGGCTGTTGCCGGTG
CTGTGCCAGGACCATGGCCTGACCCCGGCCCAAGTGGTGGCCATCGCCAACAATAACGGCGGCAAGCAGGC
GCTGGAGACGGTGCAGCGGCTGTTTCCGGTGCTGTGCCAGGACCATGGCCTGACCCCGGACCAGGTGGTGA
CCATCGCCAACAATAACGGCGGCAAGCAGGCGCTGGAGACGGTGCAGCGGCTGTTGCCGGTGCTGTGCCAG
GCCCATGGCTTGATCCCGGACCAGGTGGTGGCCATCGCCAACAATAACGGCGGCAAGCAGGCGCTGGAGAC
GGTGCAGCGGCTGTTGCCGGTGCTGTGCCAGGCCCATGGCCTGACCCCGGCCCAAGTGGTGGCCATCGCCA
GCAATATTGGCGGCAAGCAGGCGCTGGAGACGGTGCAGCGGCTGTTGCCGGTGCTGTGCCAGGCCCATGGC
CTGACCCCGGCCCAAGTGGTGGCCATCGCCAACAATAACGGCGGCAAGCAGGCGCTGGAGACGGTGCAGC
GGCTGTTGCCGGTGCTGTGCCAGGACCATGGCCTGACCCCGGATCAAGTGGTGGCCATCGCCAGCAATATTG
GCGGCAAGCAGGCGCTGGAGACGGTGCAGCGCCTGTTGCCGGTGCTGTGCCAGGCCCATGGCCTGACCCCG
GACCAGGTCGTGGCCATCGCCAGCAATGGCGGCGGCAAGCAGGCGCTGGAGACGGTGCAGCGGCTGTTGC
CGGTGCTGTGCCAGGACCATGGCCTGACCCcGGACCAGGTCGTGGCCATCGCCGGCCACGATGGCGGCAAG
CAGGCGCTGGAGACGGTGCAGCGGCTGTTGCCGGTGCTGTGCCAGGACCATGGCCTGACCCCGGACCAGGT
CGTGGCCATCGCCAGCCACGATGGCGGCAAGCAGGCGCTGGAGACGGTGCAGCGGCTGTTGCCGGTGCTGT
GCCAGGACCATGGCCTGACCCTGGACCAGGTGGTGGCCATCGCCAGCAATATTGGCGGCAAGCAGGCGCTG
GAGACGGTGCAGCGGCTGTTGCCGGTGCTGTGCCAGGACCATGGCCTGACCCCGGACCAGGTCGTGGCCAT
CGCCAGCAATGGCGGCGGCAAGCAGGCGCTGGAGAGCATTGTTGCCCAGTTATCTCGCCCTGATCCGGCGTT
GGCCGCGTTGACCAACGACCACCTCGTCGCCTTGGCCTGCCTCGGCGGACGTCCTGCCCTGGATGCAGTGA
AAAaGGGATTGCCGCACGCGCCGGAATTGATCAGAAGAATCAATCGCCGTATTCCCGAACGCACGTCCCATC
GCGTTGCCGACTACGCGCAAGTGGTTCGCGTGCTGGAGTTTTtCCAGTGCCACTCCCACCCAGCGTACGCAT
TTGATGAGGCCATGACGCAGTTCGGGATGAGCAGGAACGGGTTGGTACAGCTCTTTCGCAGAGTGGGCGTC

ACCGAACTCGAAGCCCGCGGTGGAACGCTCCCCccAGCCTCGCAGCGTTGGGACCGTATCCTCCAGGCATCA
GGGATGAAAaGGGCCAAACCGTCCCCTACTTCAGCTCAAACACCGGATCAGGCGTCTTTGCATGCATTCGCC
GATTCGCTGGAGCGTGACCTTGATGCGCCTAGCCCAATGCACGAGGGAGATCAGACaGGGGCAAGCAGCCG
TAAACGGTCCCGATCGGATCGTGCTGTCACCGGCCCCTCCGCACAGCAATCTTTCGAGGTGCGCGTTCCCGA
ACAGCGCGATGCGCTGCATTTGCCCCTCAGCTGGAGGGTAAAACGCCCGCGTACCAGGATCGGGGgCGGCC
TCCCGGATCCTGGTACGCCCATCGCTGCCGACCTGGCAGCGTCCAGCACCGTGATGTGGGAACAAGATGCG
GCCCCcTTCGCAGGGGCAGCGGATGATTTCCcGGCATTCAACGAAGAGGAGCTCGCATGGTTGATGGAGCTA
TTGCCTCAGTCAGGCTCAGTCGGAGGGACGATCTGA

>*tal1b* (3312 bp)
ATGGATCCCATTCGTTCGCGCACGCCAAGTCCTGCCCGCGAGCTTCTGCCCGGACCCCAACCGGATAGGGTT
CAGCCGACTGCAGATCGGGGGGGGGCTCCGCCTGCTGGCGGCCCCCTGGATGGCTTGCCCGCTCGGCGGAC
GATGTCCCGGACCCGGCTGCCATCTCCCCCTGCGCCCTCGCCTGCGTTCTCGGCGGGCAGCTTCAACGATCT
GCTCCGTCAGTTCGATCCGTCGCTTCTTGATACATCGCTTCTTGATTCGATGCCTGCCGTCGGCACGCCGCAT
ACAGCGGCTGCCCCAGCAGAGTGGGATGAGGTGCAATCGGGTCTGCGTGCAGCCGATGACCCGCCACCCAC
CGTGCGTGTCGCTGTCACTGCCGCGCGGCCGCCGCGCGCAAAGCCGGCCCCGCGACGGCGTGCGGCGCAA
CCCTCCGACGCTTCGCCGGCCGCGCAGGTGGATCTACGCACGCTCGGCTACAGTCAGCAGCAGCAAGAGAA
GATCAAACCGAAGGTGCGTTCGACAGTGGCGCAGCACCACGAGGCACTGGTGGGCCATGGTTTTACACACG
CGCACATCGTTGCGCTCAGCCAACACCCGGCAGCGTTAGGGACCGTCGCTGTCAAGTATCAGCACATAATCA
CGGCGTTGCCAGAGGCGACACACGAAGACATCGTTGGCGTCGGCAAACAGTGGTCCGGCGCACGCGCCCT
GGAGGCCTTGCTCACGAAGGCGGGGGAGTTGAGAGGTCCGCCGTTACAGTTGGACACAGGCCAACTTCTC
AAGATTGCAAAACGTGGCGGCGTGACCGCAGTGGAGGCAGTGCATGCATCGCGCAATGCACTGACGGGTGC
CCCCCTGAACCTGACCCCGGACCAAGTGGTGGCCATCGCCAGCAATATTGGCGGCAACCAGGCGCTGGAGA
CGGTGCAGCGGCTGTTGCCGGTGCTGTGCCAGGACCATGGCCTGACCCCGGACCAAGTGGTGGCCATCGCC
AACAATAACGGCGGCAAGCAGGCGCTGGAGACGGTGCAGCGGCTGTTGCCGGTGCTGTGCCAGGCCCATG
GCCTGACCCCGGACCAAGTGGTGGCCATCGCCAGCAATGGCGGCAAGCAGGCGCTGGAGACGGTGCAGCG
GCTGTTGCCGGTGCTGTGCCAGGCCCATGGCCTGACCCCGGACCAGGTCGTGGCCATCGCCAGCAATGGCG
GCGGCAAGCAGGCGCTGGAGACGGTGCAGCGGCTGTTGCCGGTGCTGTGCCAGGCCCATGGCCTGACCCC
GGCCCAGGTGGTGGCCATCGCCAGCAATAGTGGCGGCAAGCAGGCGCTGGAGACGGTGCAGCGGCTGTTG
CCGGTGCTGTGCCAGGACCATGGCCTGACCCCGGCCCAAGTGGTGGCCATCGCCAACAATAACGGCGGCAA
GCAGGCGCTGGAGACGGTGCAGCGGCTGTTTCCGGTGCTGTGCCAGGACCATGGCCTGACCCCGGACCAGG
TGGTGACCATCGCCAACAATAACGGCGGCAAGCAGGCGCTGGAGACGGTGCAGCGGCTGTTGCCGGTGCTG
TGCCAGGCCCATGGCTTGATCCCGGACCAGGTGGTGGCCATCGCCAACAATAACGGCGGCAAGCAGGCGCT
GGAGACGGTGCAGCGGCTGTTGCCGGTGCTGTGCCAGGCCCATGGCCTGACCCCGGCCCAAGTGGTGGCCA
TCGCCAGCAATATTGGCGGCAAGCAGGCGCTGGAGACGGTGCAGCGGCTGTTGCCGGTGCTGTGCCGGGCC
CATGGCCTGACCCCGGCCCAAGTGGTGGCCATCGCCAACAATAACGGCGGCAAGCAGGCGCTGGAGACGGT
GCAGCGGCTGTTGCCGGTGCTGTGCCAGGACCATGGCCTGACCCCGGATCAAGTGGTGGCCATCGCCAGCA
ATATTGGCGGCAAGCAGGCGCTGGAGACGGTGCAGCGCCTGTTGCCGGTGCTGTGCCAGGCCCATGGCCTG
ACCCCGGACCAGGTCGTGGCCATCGCCAGCAATGGCGGCGGCAAGCAGGCGCTGGAGACGGTGCAGCGGC
TGTTGCCGGTGCTGTGCCAGGACCATGGCCTGACCCCGGACCAGGTCGTGGCCATCGCCGGCCACGATGGC

GGCAAGCAGGCGCTGGAGACGGTGCAGCGGCTGTTGCCGGTGCTGTGCCAGGACCATGGCCTGACCCCGG
ACCAGGTCGTGGCCATCGCCAGCCACGATGGCGGCAAGCAGGCGCTGGAGACGGTGCAGCGGCTGTTGCC
GGTGCTGTGCCAGGACCATGGCCTGACCCTGGACCAGGTGGTGGCCATCGCCAGCAATATTGGCGGCAAGC
AGGCGCTGGAGACGGTGCAGCGGCTGTTGCCGGTGCTGTGCCAGGACCATGGCCTGACCCCGGACCAGGTC
GTGGCCATCGCCAGCAATGGCGGCGGCAAGCAGGCGCTGGAGAGCATTGTTGCCCAGTTATCTCGCCCTGAT
CCGGCGTTGGCCGCGTTGACCAACGACCACCTCGTCGCCTTGGCCTGCCTCGGCGGACGTCCTGCCCTGGAT
GCAGTGAAAAAGGGATTGCCGCACGCGCCGGAATTGATCAGAAGAATCAATCGCCGTATTCCCGAACGCAC
GTCCCATCGCGTTGCCGACTACGCGCAAGTGGTTCGCGTGCTGGAGTTTTTCCAGTGCCACTCCCACCCAGC
GTACGCATTTGATGAGGCCATGACGCAGTTCGGGATGAGCAGGAACGGGTTGGTACAGCTCTTTCGCAGAGT
GGGCGTCACCGAACTCGAAGCCCGCGGTGGAACGCTCCCCCCAGCCTCGCAGCGTTGGGACCGTATCCTCC
AGGCATCAGGGATGAAAAGGGCCAAACCGTCCCCTACTTCAGCTCAAACACCGGATCAGGCGTCTTTGCAT
GCATTCGCCGATTCGCTGGAGCGTGACCTTGATGCGCCTAGCCCAATGCACGAGGGAGATCAGACAGGGGC
AAGCAGCCGTAAACGGTCCCGATCGGATCGTGCTGTCACCGGCCCCTCCGCACAGCAATCTTTCGAGGTGC
GCGTTCCCGAACAGCGCGATGCGCTGCATTTGCCCCTCAGCTGGAGGGTAAAACGCCCGCGTACCAGGATC
GGGGGCGGCCTCCCGGATCCTGGTACGCCCATCGCTGCCGACCTGGCAGCGTCCAGCACCGTGATGTGGGA
ACAAGATGCGGCCCCCTTCGCAGGGGCAGCGGATGATTTCCCGGCATTCAACGAAGAGGAGCTCGCATGGT
TGATGGAGCTATTGCCTCAGTCAGGCTCAGTCGGAGGGACGATCTGA

Figure S14. Nucleotide sequences of the *tal* genes tal6b from LN18 and *tal1b* from LN4.

Table S1 Strains and plasmids used in this study.

Designation	Description	Reference/Source
**Bacterial Strains**		
Escherichia coli		
DH5α	F^-endA1, *thi-1*, *recA1*, Φ80, *lacZ*, ΔM15	TransGen Biotech
BL21(DE3)	F^- *ompT*, *hsdS20*, *gal*	TransGen Biotech
X. oryzae pv. *oryzae*		
PXO99^A	Wild-type, Philippine race 6; harbors *avrXa27*	Hopkins et al., 1992
LN18	Wild-type, Chinese isolate, harbors *tal6b* (*avrXa27A*)	This lab
LN4	Wild-type, Chinese isolate, harbors *tal1b* (*avrXa27A*)	This lab
AH28	Wild-type, Chinese isolate	This lab
HuN37	Wild-type, Chinese isolate; harbors *tal17* (*avrXa27B*)	Lifang Ruan, Huazhong Agricultural University,
IXO221	Wild-type, Indian isolate; harbors *tal6c* (*avrXa27C*)	This lab
PH	Derived from PXO99^A, *tal*-free strain	Ji et al., 2016
PH/ev	PH with empty vector pHM1	This lab
PH/avrXa27	PH with pHZW-avrXa27	This lab
PH/pthXo1	PH with pHZW-pthXo1	This lab
PH/avrXa7	PH with pHZW-avrXa7	This lab
PH/tal6b	PH with pHZW-tal6b; *tal6b* is also known as *avrXa27A*	This lab
PH/tal17	PH with pHZW-tal17; *tal17* is also known as *avrXa27B*	This study
PH/tal6c	PH with pHZW-tal6c; tal6c is also known as *avrXa27C*	This study
PH/tal6b-2	PH with pHZW-tal6b-2 (mutated variant of *tal6b*)	This study
PH/tal6b-6	PH with pHZW-tal6b-6 (mutated variant of *tal6b*)	This study

**Plasmids**		
pHM1	Broad-spectrum cosmid vector, Sp^{r}	Hopkins et al., 1992
pZW-avrXa7	*avrXa7* in pBluescript II KS+, contains a FLAG epitope immediately downstream of the second *Sph*I site in the C-terminus of AvrXa7, Ap^{r}	Yang *et al.*, 2000
pHZW-avrXa7	pHM1 fused with pZW-avrXa7 at *Hin*dIII site; contains *lacZ* promoter upstream of *avrXa7*, Ap^{r}, Sp^{r}	Yang *et al.*, 2000
pZW-avrXa27	*Sph*I fragment of *avrXa27* in pZW-avrXa7; contains a FLAG epitope immediately downstream of the second *Sph*I site in the C-terminus of AvrXa27,Ap^{r}	Ma et al., 2018
pHZW-avrXa27	pHM1 fused with pZW-avrXa27 at *Hin*dIII, *lacZ* promoter upstream of *avrXa27*, Ap^{r}, Sp^{r}	Ma et al., 2018
pHZW-pthXo1	pHM1 fused with pZW-pthXo1 at *Hin*dIII site; *lacZ* promoter upstream of *pthXo1*, which originated from *Xoo* PXO99^{A}; Ap^{r}, Sp^{r}	Ma et al., 2018
pZW-tal6b	*Sph*I fragment of *tal6b* in pZW-avrXa7; contains a FLAG epitope immediately downstream of the second *Sph*I site in the C-terminus of *tal6b*, which originated	This lab
pHZW-tal6b	pHM1 fused with pZW-tal6b at *Hin*dIII, *lacZ* promoter upstream of *tal6b*, Ap^{r}, Sp^{r}	This lab
pZW-tal17	*Sph*I fragment of *tal17* in pZW-avrXa7; contains a FLAG epitope immediately downstream of the second *Sph*I site in the C-terminus of *tal17*, which originated	This study
pHZW-tal17	pHM1 fused with pZW-tal17 at *Hin*dIII, *lacZ* promoter upstream of *tal17*, Apr, Spr	This study
pZW-tal6c	*Sph*I fragment of *tal6c* in pZW-avrXa7; contains a FLAG epitope immediately downstream of the second *Sph*I site in the C-terminus of *tal6c*, which originated	This study
pHZW-tal6c	pHM1 fused with pZW-tal6c at *Hin*dIII, contains *lacZ* promoter upstream of *tal6c*, Ap^{r}, Sp^{r}	This study
pZW-tal6b-2	*Sph*I fragment of *tal6b-2* in pZW-avrXa7; contains a FLAG epitope immediately downstream of the second *Sph*I site in the C-terminus of *tal6b-2*, which is a	This study
pHZW-tal6b-2	pHM1 fused with pZW-tal6b-2 at HindIII, contains *lacZ* promoter upstream of *tal6b-2*, Ap^{r}, Sp^{r}	This study

pZW-tal6b-6	*Sph*I fragment of *tal6b-6* in pZW-avrXa7; contains a FLAG epitope immediately downstream of the second *Sph*I site in the C-terminus of *tal6b-6*, which is a	This study
pHZW-tal6b-6	pHM1 fused with pZW-tal6b-6 at *Hin*dIII, contains *lacZ* promoter upstream of *tal6b-6*, Ap^{r}, Sp^{r}	This study
pET30a	pBR322 origin, F1 origin, *lacI*, His-Tag, S-Tag, Km^{r}	Novagen
pET30a-avrXa27	pET30a containing *avrXa27* with N-terminal His-tag, Km^{r}	This study
pET30a-tal6b	pET30a containing *tal6b* with N-terminal His-tag, Km^{r}	This study
pET30a-avrXa27B	pET30a containing *avrXa27B* with N-terminal His-tag, Km^{r}	This study
pET30a-avrXa27C	pET30a containing *avrXa27C* with N-terminal His-tag, Km^{r}	This study
pET30a-pthXo1	pET30a containing *pthXo1* with N-terminal His-tag, Km^{r}	This lab
pHB	Binary vector, double 35S promoter, 3×N-terminal FLAG tag, Km^{r}	Mao et al., 2005
pCAMBIA1381	Binary vector containing promoterless *gusA*; used for GUS assays, Km^{r}	This lab
pHB-pthXo1	*pthXo1* cloned with N-terminal flag-tag in pHB, Km^{r}	This lab
pHB-tal6b	*Tal6b* cloned with N-terminal flag-tag in pHB, Km^{r}	This study
OsSWEET11ap∷GUS	97bp promoter region of *OsSWEET11a* cloned upstream of *gusA* in pCAMBIA1381	This study
Xa27p∷GUS	160bp promoter region of *Xa27* cloned upstream of *gusA* in pCAMBIA1381	This study

Table S2 ***Xoo*** **strains used for detection of AvrXa27/TalAO** ***tal*** **genes.**

**Strain**	**origin**	**Accession number**	**Presence (+) and absence (-) of AvrXa27/TalAO *tal* genes**
AH28	Anhui, China	NZ_CP074076	-
HuN37	Hunan, China	NZ_CP031456	+
JL25	Jilin, China	NZ_CP031457	-
JL28	Jilin, China	NZ_CP031458	-
JL33	Jilin, China	NZ_CP031459	+
LN18	Liaoning, China	CP045238	+
LN4	Liaoning, China	CP045452	+
ScYc-b	Sichuan, China	NZ_CP018087	-
XF89b	Taiwan, China	NZ_CP011532	+
XM9	Taiwan, China	NZ_CP020334	+
YC11	Jiangsu, China	NZ_CP031464	+
YN24	Yunan, China	CP018089	-
BXO1	India	CP033201	+
ICMP3125	India	NZ_CP031697	+
ITCCBB0002	India	NZ_CP046148	-
IX-280	India	NZ_CP019226	+
IXO1088	India	NZ_CP040687	-
IXO704	India	NZ_CP040604	-
JP01	Japan	NZ_CP031460	+
JW11089	South Korea	NZ_CP033193	+
K1	South Korea	NZ_CP049205	+
K2	South Korea	NZ_CP050113	+
K3	South Korea	NZ_CP050114	+
K3a	South Korea	NZ_CP050115	-
KACC 10331	South Korea	NC_006834	-
KXO85	South Korea	NZ_CP033197	+
MAFF 311018	Japan	NC_007705	+
NX0260	Nepal	NZ_CP033192	-
PXO142	Philippines	NZ_CP031698	+
PXO145	Philippines	NZ_CP013961	+
PXO211	Philippines	NZ_CP013674	+
PXO236	Philippines	NZ_CP013675	+
PXO282	Philippines	NZ_CP013676	+
PXO364	Philippines	NZ_CP033191	-
PXO404	Philippines	NZ_CP033190	+
PXO421	Philippines	NZ_CP033189	+
PXO513	Philippines	NZ_CP033188	+
PXO524	Philippines	NZ_CP013677	+

PXO563	Philippines	NZ_CP013678	+
PXO602	Philippines	NZ_CP013679	+
PXO61	Philippines	NZ_CP033187	+
PXO71	Philippines	NZ_CP013670	+
PXO79	Philippines	NZ_CP031462	-
PXO83	Philippines	NZ_CP012947	+
PXO86	Philippines	NZ_CP007166	+
PXO99[A]	Philippines	NC_010717	+
SK2-3	Thailand	NZ_CP019515	+
AXO1947	Cameroon	NZ_CP013666	-
BAI3	Burkina Faso	NZ_CP025610	-
CFBP1948	Cameroon	NZ_CP033185	-
CFBP1949	Mali	NZ_CP033184	-
CFBP1951	Mali	NZ_CP033183	-
CFBP1952	Mali	NZ_CP033182	-
CFBP7319	Burkina Faso	NZ_CP033181	-
CFBP7320	Burkina Faso	NZ_CP033186	-
CFBP7322	Burkina Faso	NZ_CP033179	-
CFBP7323	Niger	NZ_CP033178	-
CFBP7324	Niger	NZ_CP033177	-
CFBP7337	Mali	NZ_CP033175	-
CFBP7340	Niger	NZ_CP033174	-
CFBP8172	Benin	NZ_CP033173	-
CIX2374	Senegal	NZ_CP036377	-
Dak16	Tanzania	NZ_CP033172	-
MAI1	Mali	NZ_CP025609	-
MAI106	Mali	NZ_CP019089	-
MAI129	Mali	NZ_CP019090	-
MAI134	Mali	NZ_CP019091	-
MAI145	Mali	NZ_CP019092	-
MAI68	Mali	NZ_CP019085	-
MAI73	Mali	NZ_CP019086	-
MAI95	Mali	NZ_CP019087	-
MAI99	Mali	NZ_CP019088	-
T19	Tanzania	NZ_CP033171	-
Ug11	Uganda	NZ_CP033170	-
BAI23	Burkina Faso	NZ_CP036256	-
CFBP7321	Burkina Faso	NZ_CP033180	-
CFBP7325	Mali	NZ_CP033176	-
CIX298	Burkina Faso	NZ_CP036378	-
CIAT	Colombia	NZ_CP033194	+
AUST2013	Australia	NZ_CP033196	+

Table S3 Primers used in this study

Primer name	Sequence (5′-3′)	Purpose
Actin-F	GTTCCTGCTGTTTGTTCTGTTG	Amplifies *Actin* as a 137-bp fragment; used for qRT-PCR
Actin-R	ATCTCACGCATTACCCTACCTT	
OsSWEET11a-qF	AGTCGACGGGAGGGTACAG	Amplifies *OsSWEET11* as a 111-bp fragment; used for qRT-PCR
OsSWEET11a-qR	TGATGGTCAGCAGCGGC	
OsSWEET13-qF	GCCTGTCCCTGCAGCATC	Amplifies *OsSWEET13* as a 122-bp fragment; used for qRT-PCR
OsSWEET13-qR	CTCCGTCGACTTGCTCTTGTAG	
OsSWEET14-qF	TCTACGCCCCCAAGAAGGCCA	Amplifies *OsSWEET14* as a 136-bp fragment; used for qRT-PCR
OsSWEET14-qR	ACCCAACCAAGAACCACGATGC	
Xa27-qF	CTCGCCATGCTGTCGCTCGT	Amplifies *Xa27* as a 101-bp fragment; used for qRT-PCR
Xa27-qR	TAGAGAGACCAGAGACCACCAAGCA	
TALN18-F	GCCGATGACCCGCCACCCAC	Amplifies the N-terminal region of *tal* genes; used for PCR
TALN18-R	TGTCTTCGTGTGTCGCCTCT	
Xa27p-F	GTGCTATAAATAGAAGAAGAGACCCATAGAGA GC	Amplifies probe Xa27p; used for EMSA
Xa27p-R	GCTCTCTATGGGTCTCTTCTTCTATTTATAGCAC	
Xa13p-F	GTTAGATATGCATCTCCCCCTACTGTACACCAC CAAAAGT	Synthesis of probe Xa13p for EMSA
Xa13p-R	ACTTTTGGTGGTGTACAGTAGGGGGAGATGCA TATCTAAC	
Xa13p-1F	CTCCCCCTACTGTACACCAC	Synthesis of probe Xa13p-1 for EMSA
Xa13p-1R	GTGGTGTACAGTAGGGGGAG	
Xa13p-2F	CTACTGTACACCACCAAAAG	Synthesis of probe Xa13p-2 for EMSA
Xa13p-2R	CTTTTGGTGGTGTACAGTAG	
Xa13p-3F	GTTAGATACCCCTACTGTACACCAC	Synthesis of probe Xa13p-3 for EMSA
Xa13p-3R	GTGGTGTACAGTAGGGGTATCTAAC	
Xa13p-4F	GTTAGATACTACTGTACACCACCAAAAG	Synthesis of probe

Xa13p-4R	CTTTTGGTGGTGTACAGTAGTATCTAAC	Xa13p-4 for EMSA
Xa13p-5F	GTTAGATATGCATCTCCCCCTtagGTACACCACCAAAAGT	Synthesis of probe Xa13p-5 for EMSA
Xa13p-5R	ACTTTTGGTGGTGTACctaAGGGGGAGATGCATATCTAAC	
Xa13p-6F	GTTAGATATGCATCTCCCCCT	Synthesis of probe Xa13p-6 for EMSA
Xa13p-6R	AGGGGGAGATGCATATCTAAC	
Xa13p-7F	ACAGTAGGGGGAGATGCATA	Synthesis of probe Xa13p-7 for EMSA
Xa13p-7R	TATGCATCTCCCCCTACTGT	
Xa13p-8F	TGCATCTCCCCCTACTGTAC	Synthesis of probe Xa13p-8 for EMSA
Xa13p-8R	GTACAGTAGGGGGAGATGCA	
Xa13p-9F	GCATCTCCCCCTACTGTACACC	Synthesis of probe Xa13p-9 for EMSA
Xa13p-9R	GGTGTACAGTAGGGGGAGATGC	
Xa13p-10F	ATCTCCCCCTACTGTACAC	Synthesis of probe Xa13p-10 for EMSA
Xa13p-10R	GTGTACAGTAGGGGGAGAT	
Xa13p-11F	AGCTCCCCCTACTGTACAC	Synthesis of probe Xa13p-11 for EMSA
Xa13p-11R	GTGTACAGTAGGGGGAGCT	
Xa13p-12F	AGCTGCCCCTACTGTACAC	Synthesis of probe Xa13p-12 for EMSA
Xa13p-12R	GTGTACAGTAGGGGCAGCT	
Xa13p-13F	AGCTGCCCCTGCTGTACAC	Synthesis of probe Xa13p-13 for EMSA
Xa13p-13R	GTGTACAGCAGGGGCAGCT	
Xa13p-14F	ATCTCCCCCTACTCTACAT	Synthesis of probe Xa13p-14 for EMSA
Xa13p-14R	ATGTAGAGTAGGGGGAGAT	
Xa27p-F1	cagagtgaaaaagaaGGATCCAAACACCACCTAAGGTT	Amplifies the promoter region of *Xa27* for cloning in pCAMBIA1381
Xa27p-R1	ggtggactcctcttaaagcttTGGAGGCAGCTTCTTGGGT	

Supplemental References

Hopkins, C. M., White, F. F., Choi, S. H., Guo, A. and Leach, J. E. (1992) Identification of a family of avirulence genes from *Xanthomonas oryzae* pv. *oryzae*. Molecular Plant-Microbe Interaction 5, 451-459.

Ji, Z., Ji, C., Liu, B., Zou, L., Chen, G. and Yang, B. (2016) Interfering TAL effectors of *Xanthomonas oryzae* neutralize R-gene-mediated plant disease resistance. Nature Communications, 7, 13435.

Ma, W., Zou, L., Zhiyuan, J.I., Xiameng, X.U., Zhengyin, X.U., Yang, Y., Alfano, J.R., and Chen, G. (2018). Xanthomonas oryzae pv. oryzae TALE proteins recruit OsTFIIA γ 1 to compensate for the absence of OsTFIIA γ 5 in bacterial blight in rice. Molecular Plant Pathology 19, 2248 – 2262.

Yang, B., Zhu, W., Johnson, L. B. and White, F. F. (2000) The virulence factor AvrXa7 of *Xanthomonas oryzae* pv. *oryzae* is a type III secretion pathway-dependent nuclear-localized double-stranded DNA-binding protein. Proceedings of the National Academy of Sciences of the United States of America, 97, 9807-9812.

Mao, J., Zhang, Y., Sang, Y., Li, Q., and Yang, H. (2005). A role for *Arabidopsis* cryptochromes and COP1 in the regulation of stomatal opening. Proc. Natl. Acad. Sci. USA 102:12270-12275.
